# Unusual Calixarenes Incorporating Chromene and Benzofuran Moieties Obtained via Propargyl Claisen Rearrangement

**DOI:** 10.1021/acs.orglett.1c03643

**Published:** 2021-11-15

**Authors:** Annunziata Soriente, Mariantonietta D’Acunto, Carmen Talotta, Carmine Gaeta, Paolo Della Sala, Margherita De Rosa, Silvano Geremia, Neal Hickey, Antonio Rescifina, Placido Neri

**Affiliations:** † Dipartimento di Chimica e Biologia “A. Zambelli”, Università di Salerno, Via Giovanni Paolo II 132, I-84084 Fisciano, Salerno, Italy; ‡ Centro di Eccellenza in Biocristallografia, Dipartimento di Scienze Chimiche e Farmaceutiche, Università di Trieste, Via L. Giorgieri 1, I-34127 Trieste, Italy; § Dipartimento di Scienze del Farmaco e della Salute, Università di Catania, Viale Andrea Doria 6, I-95125 Catania, Italy

## Abstract

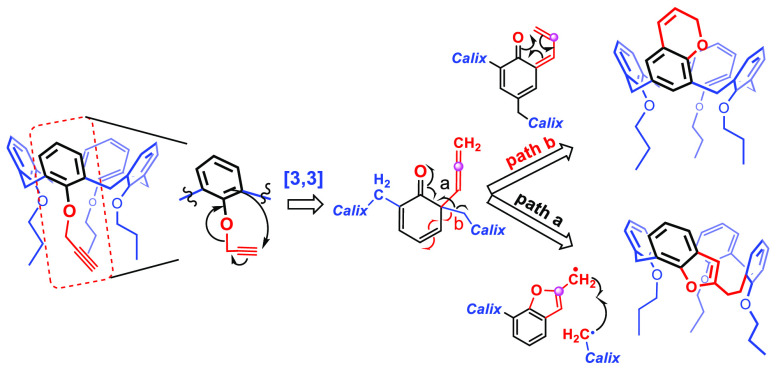

Monopropargyloxy-tripropoxy-calix[4]arene **1** was subjected to a propargyl Claisen rearrangement to give unusual calix[3]arene[1]chromene and homocalix[3]arene[1]benzofuran macrocycles. Quantum mechanical density functional theory calculations indicated that an initial [3,3] sigmatropic reaction affords a highly reactive allene intermediate, stabilized by two main diradical pathways leading to six- and five-membered oxygenated rings. In the presence of a *n*-butylammonium guest, calix[3]arene[1]chromane **6** forms two stereoisomeric complexes stabilized by ^+^N–H···O and cation···π interactions.

A wide variety of calixarene-based supramolecular hosts continue to be obtained even today by innovative chemical modifications of the parent macrocycles.^1^ In this way, surprising supramolecular properties are continually discovered for appropriately modified calixarene derivatives.^1^ The most common modification sites are at the lower and upper rims^2a^ (OH groups and *para* positions,^2a^ respectively),^1^ as well as the *meta* positions^2b^ and the methylene bridges^3^ of the calixarene skeleton. One of the earliest approaches to modifying the calixarene upper rim was the “Claisen rearrangement route” devised by Gutsche,^4^ in which allyl groups at the lower rim (i.e., allyl ethers) are transferred at the upper rim by thermal rearrangement. An exciting extension of this route could be obtained by using propargyl groups in place of the allyl ones.^5^ It is well documented^5^ that six- and five-membered oxygenated rings are obtained in the propargyl Claisen rearrangements when the *ortho* positions are free. Calix[4]arene derivatives show a peculiar three-dimensional bowl-shaped structure in which the *ortho* positions are occupied by methylene-bridging groups. Consequently, propargyl Claisen rearrangements starting by calix[4]arene derivatives could give very interesting derivatives with structures that are challenging to predict.

Intrigued by these considerations, we decided to investigate this propargyl Claisen reaction using the monopropargyl ether of tripropylated *p*-H-calix[4]arene **1** (Supporting Information and Scheme 1) as an appropriate model compound. Thus, **1** was subjected to heating in refluxing diethylaniline (215 °C) for 2 h. Chromatography of the crude product allowed the isolation of **2–4** in 48%, 15%, and 10% yields, respectively (Scheme 1).

**Scheme 1:**
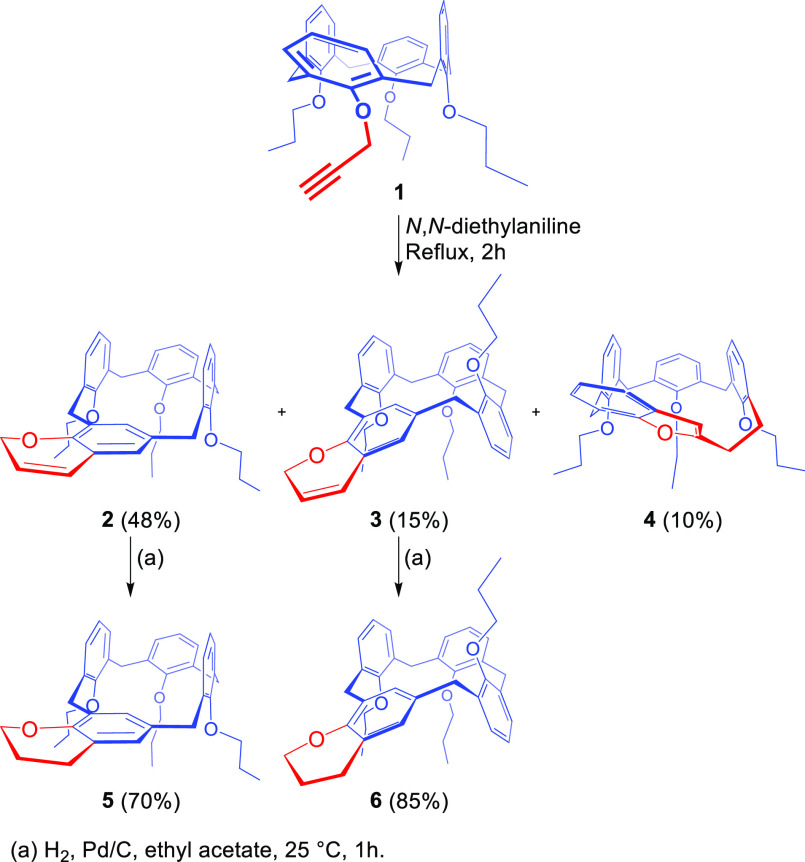
Propargyl Claisen Rearrangement Performed on Propargyloxy-Tripropoxy-Calix[4]arene **1**

The absence of the typical alkyne methine signal in the ^1^H NMR spectrum of each isolated compound (Supporting Information) was clear evidence that the postulated double sigmatropic migration at the *para* position did not occur, while the presence of oxygenated rings was at first glance confirmed by signals in the range of 4–6 ppm. One-dimensional (1D) and two-dimensional (2D) NMR data and MS analysis (Supporting Information) agree with the calix[3]arene[1]chromene structure of **2** in Scheme 1. In particular, **2** showed an entire spin system (Figure 1a) at 4.85 ppm (2H, red signal), 5.81 ppm (1H, blue signal), and 6.50 ppm (1H, green signal) in the COSY spectrum, strongly pointing to a chromene moiety. This was confirmed by two aromatic signals at 5.57 and 6.73 ppm, which were unexpectedly *meta*-coupled (*J* = 1.6 Hz) to each other. Another relevant feature of the ^1^H NMR spectrum of **2** was the loss of the symmetry element present in the starting **1**, which led to triplicate sets of signals (often accidentally isochronous) corresponding to three non-equivalent (PrO)ArCH_2_ moieties of **2**. It was clear that the propargylated aromatic ring of **1** was rearranged to a chromene moiety by a sigmatropic reaction, which also involved the migration of the adjacent ArCH_2_ linkage to the original *para* position. The bridging ArCH_2_Ar protons of **2** give rise to four AX systems with a Δδ of 0.5–1.1 ppm typical of a cone conformation,^6^ indicating that this shape was maintained during the transposition mentioned above.

**Figure 1:**
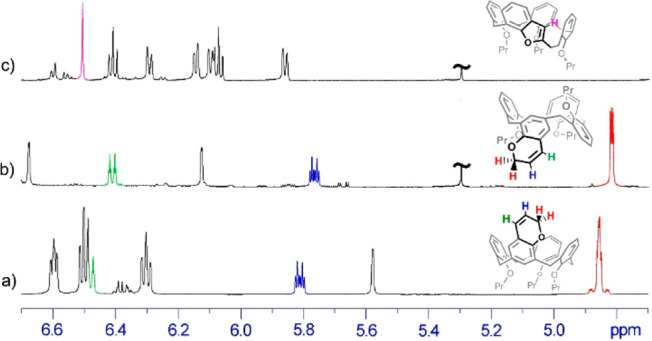
Significative regions of the ^1^H NMR spectra (600 MHz, CDCl_3_) of (a) **2**, (b) **3**, and (c) **4**.

The ^1^H NMR spectrum of **3** clearly indicated its stereoisomeric nature with respect to **2**. In fact, the chromene ring was confirmed by a spin system (Figure 1b) at 4.82 ppm (2H, red signal), 5.77 ppm (1H, blue signal), and 6.42 ppm (1H, green signal) and by two *meta*-coupled ArH features at 6.13 and 6.69 ppm (Figure 1b).

The most evident difference was a more pronounced differentiation in the chemical shifts of the triplicate sets of signals corresponding to the three non-equivalent (OPr)ArCH_2_ moieties. This, for example, led the three CH_3_ signals of OPr groups to resonate at 0.49, 0.67, and 0.93 ppm, indicating a strong shielding by aromatic moieties for two of them. Also, a clear diagnostic difference was also detected in the COSY and HSQC spectra of **3**. The bridging ArCH_2_Ar groups of **3** give rise to an AX system at 3.28/4.38 ppm (Δδ = 1.1) that correlates in the HSQC spectrum with a carbon resonance at 30.5 ppm, attributable to a methylene group between *syn*-oriented aromatic rings.^6^ An AB system at 3.92/3.98 ppm (Δδ = 0.06) was detected in the COSY spectrum of **3** that correlates with a methylene carbon at 38.3 ppm between *anti*-oriented rings,^7^ and an AB system at 3.36/3.85 ppm (Δδ = 0.49) that shows a ^1^*J* with a ^13^C signal at 34.7 ppm attributable to a ArCH_2_Ar group between *anti*-oriented rings.^7^ Finally, an AB system at 3.72/3.81 ppm (Δδ = 0.09) was present that correlates in the HSQC at 28.3 ppm, clearly indicative of a *syn* relationship between the pertinent rings.^6,7^ In conclusion, the presence of two *anti*-oriented and two *syn*-oriented ArCH_2_Ar groups is compatible with a calix[4]arene backbone of **3** in a 1,2-alternate conformation (Scheme 1). DFT-optimized structures at the B3LYP/6-31G(d,p) level of theory show an energy difference of 1.02 kcal/mol between cone **2** and 1,2-alternate **3** conformers (Supporting Information). In addition, the DFT-optimized structure of **2** exhibits the chromene ring in the outward orientation almost coplanar with the mean plane of methylene bridges.

Calix-chromenes **2** and **3** easily undergo degradation due to the presence of the unsaturated chromene ring. Therefore, we decided to prepare more stable analogues by hydrogenating the double bond (Scheme 1). Thus, the treatment of **2** and **3** with H_2_ and Pd/C easily afforded the corresponding chromane derivatives **5** and **6** in good yields (Scheme 1; see the Supporting Information). In the solid state, **6** adopts a 1,2-alternate conformation (Figure 2), in which the chromane moiety (A) and an adjacent Ar-OPr ring (B) show an inverted conformation with respect to the other two (C and D) Ar-OPr rings (Figure 2).

**Figure 2:**
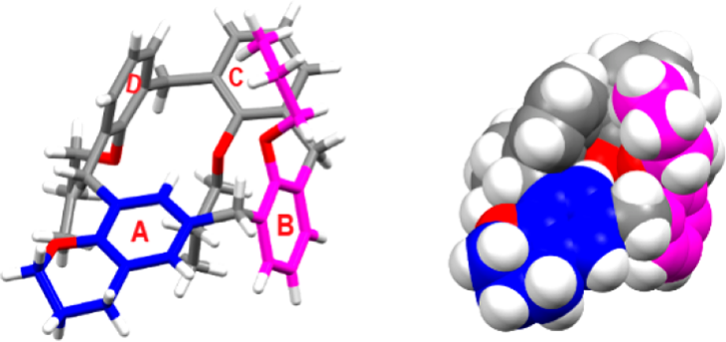
X-ray crystal structure of **6** showing its 1,2-alternate conformation in which rings A and B are inverted with respect to the other two (C and D).

The overall conformation of the calixarene can be described by the four dihedral angles between the mean plane of each Ar ring and the mean plane defined by the methylene-bridging groups.^8^ The mean planes of the phenyl rings of chromane and its aromatic facing group (A and C) make large outward dihedral angles (161° and −140°, respectively) (Figure 2). Aryl ring B is inclined outward (dihedral angle of 111°), while aryl ring D is inclined slightly inward (dihedral angle of −84°).

The presence of an H atom at the *endo* position of the chromane moiety allows this very large dihedral angle of 161°, suggesting the possibility of facile inversion of its conformation, from positive to negative angles, and therefore between the 1,2-alternate and partial-cone conformation of the calixarene.

With regard to derivative **4** (Scheme 1), its ^1^H NMR spectrum (Figure 1c) evidenced a different signal pattern with respect to the ^1^H NMR spectra of **2** and **3** (Figure 1a,b). In fact, the chromene signals were absent, while four resonances (1H each) appeared in the range of 2.5–3.5 ppm, pointing to the presence of an ethylene -CH_2_CH_2_- bridge moiety. In addition, an aromatic signal was seen at 6.51 ppm (1H, magenta signal), suggesting the formation of a fused furan ring (Figure 1c). The combined use of 2D COSY and HSQC spectra (Supporting Information) allowed joining of the two moieties leading to structure **4** in which a benzofuran system is linked to an Ar(OPr) unit through a -CH_2_CH_2_- bridge. In detail, three AX systems with a Δδ of 1.0–1.2 ppm for the bridging ArCH_2_Ar protons of **4** confirmed the presence of an unaltered cone portion for the remaining three Ar(OPr) units. All of the remaining NMR (^13^C and HSQC) and MS data were in full agreement with the homocalix[3]arene[1]benzofuran structure of **4**.^9^

To understand how the rearranged structures of **2–4** can be formed from **1**, we decided to perform a QM DFT study (Figure 3 and Supporting Information) using the Gaussian-16 suite of programs. In the literature, it is proposed that an aryl propargyl Claisen rearrangement usually proceeds through a first [3,3] sigmatropic migration, which leads to an allene intermediate.^9^ Due to the entity of the system (C_40_H_44_O_4_), all calculations were performed at the B3LYP level of theory employing the 6-31+G* basis set (Supporting Information). This combination, benchmarked versus several other DFT methods for the aryl propargyl ether Claisen rearrangement, gives the better performance in terms of CPU time and calculated activation energy.^9,10^ In some aryl Claisen rearrangement cases, if the *ortho* positions are occupied, a second rearrangement occurs to the *para* position after the first one.^11^ In our instance, none of the obtained products derives from this kind of reaction; instead, it is the benzyl group that rearranges to the *para* position. Therefore, the observed products could be obtained through a concerted suprafacial [1,3] sigmatropic transposition with inversion of the configuration or through a diradical mechanism.^11^ To obtain direct evidence for a diradical path, we conducted a radical trap reaction with TEMPO. When the Claisen reaction in Scheme 1 was performed in the presence of an excess of TEMPO, no hint of products **2–4** was detected in the reaction mixture. These results corroborated the radical mechanism proposed by DFT calculations. Our proposed pathway (Figure 3) was derived by studying both ionic and radical mechanisms, which can both be satisfactorily studied at the B3LYP/6-31+G* level of theory.^9b^ All calculated energies involved are reassumed in Table S2 and pictorially shown in Figure 3. The first step is the classic concerted [3,3] Claisen rearrangement, which is even more demanding in terms of Gibbs free energy of activation [32.86 kcal/mol (Table S2, entry 2)]. The successive concerted [3,3] or 1,3 sigmatropic rearrangements to the *para* position of the allene or *o*-benzyl moieties involves a Δ*G*^#^ of 34.24 or 64.49 kcal/mol, respectively; on the contrary, the homolytic cleavage of the *o*-C–C benzyl bond results in a Δ*G*^#^ of 23.66 kcal/mol (Table S1, entry 4). We were not able to find a concerted diradical TS for the Int1 to Int3b path. This is in accord with a stepwise radical mechanism and the formation of compound **4**, which cannot take place through a concerted reaction mechanism. The analysis of Table S2 and Figure 3 shows that the reaction begins with a typical Claisen rearrangement and continues through a radical mechanism that governs the ratio of the product through transition states TS3a and TS3b passing from intermediates Int2a and Int2b, in the triplet state, that are in equilibrium with each other. The optimization of Int2a and Int2b as singlet states was unfruitful. Finally, the inversion of the steps from Int2a to **4** gives an activation energy of 22.66 kcal/mol for the first TS, ruling out this pathway.

**Figure 3:**
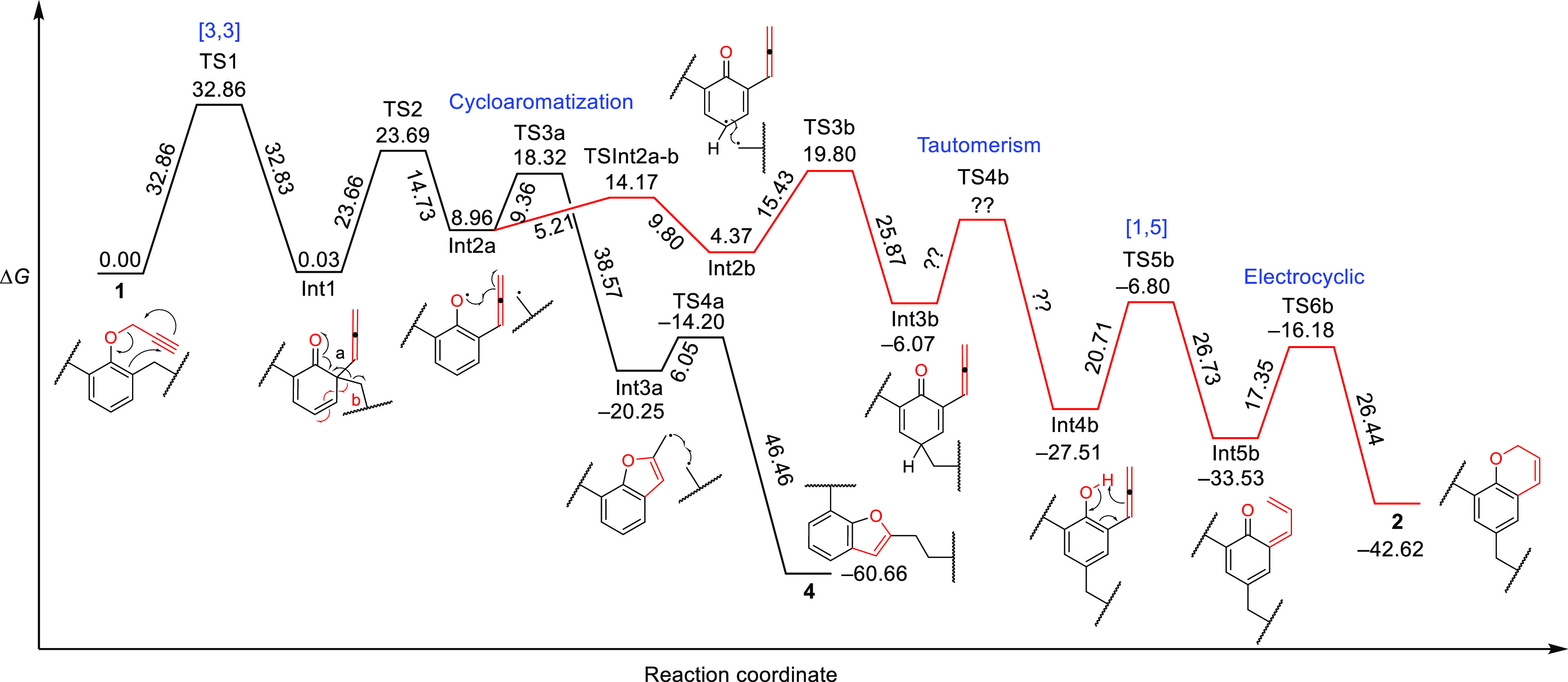
Proposed mechanism of the Claisen rearrangement performed on propargyloxy-tripropoxy-calix[4]arene **1** and its reaction coordinate diagram.

At this point, we decided to perform preliminary tests to study the complexation properties of calix-chromane **6**. The addition of *n*-butylammonium guest (as barfate salt) to a CD_2_Cl_2_ solution of **6** (in equimolar ratio) clearly evidenced significant changes in its ^1^H NMR spectrum at 298 K (Supporting Information) indicative of the formation of complexes.^12^ 1D and 2D NMR analysis of this mixture evidenced the presence of two complexes in a 60/40 ratio (Supporting Information). In detail, three methylene-bridged AX systems and one AB system were present at 3.53/4.63, 3.24/4.32, 3.21/4.25, and 3.56/3.70 ppm that represent the most abundant complex. In addition, AX/AB systems were present at 3.40/4.14, 3.18/4.02, 3.15/4.00, and 3.33/3.75 ppm attributable to the less abundant complex (Supporting Information). Accordingly with the 1,2-alternate structure of **6** (Figure 2), the cationic guest could be nested on one side of macrocycle **6** between the pair of *syn*-oriented propoxy chains [*syn*-OPr (Figure 4a)], as well as on the opposite side between the *syn*-oriented aromatic rings [*syn*-Ar (Figure 4d)]. DFT calculations at the B3LYP/6-31G(d,p) level of theory indicated that the *syn*-OPr stereoisomer (Figure 4a) is more stable than the *syn*-Ar one by 0.18 kcal/mol. Consequently, a Boltzmann population at 298 K was calculated to be 58% and 42% for the *syn*-OPr and *syn*-Ar complexes, respectively. The DFT-optimized structure of the most stable *syn*-OPr complex (Figure 4a) shows two ^+^N–H···O H-bonds with a mean distance of 2.82 Å and a mean ^+^N–H···O angle of 160° (Figure 4b); in addition, a cation···π interaction (Figure 4c) was detected between the ammonium group of the guest and an aromatic ring of the host with a ^+^N···π^centroid^ distance of 3.37 Å.

**Figure 4:**
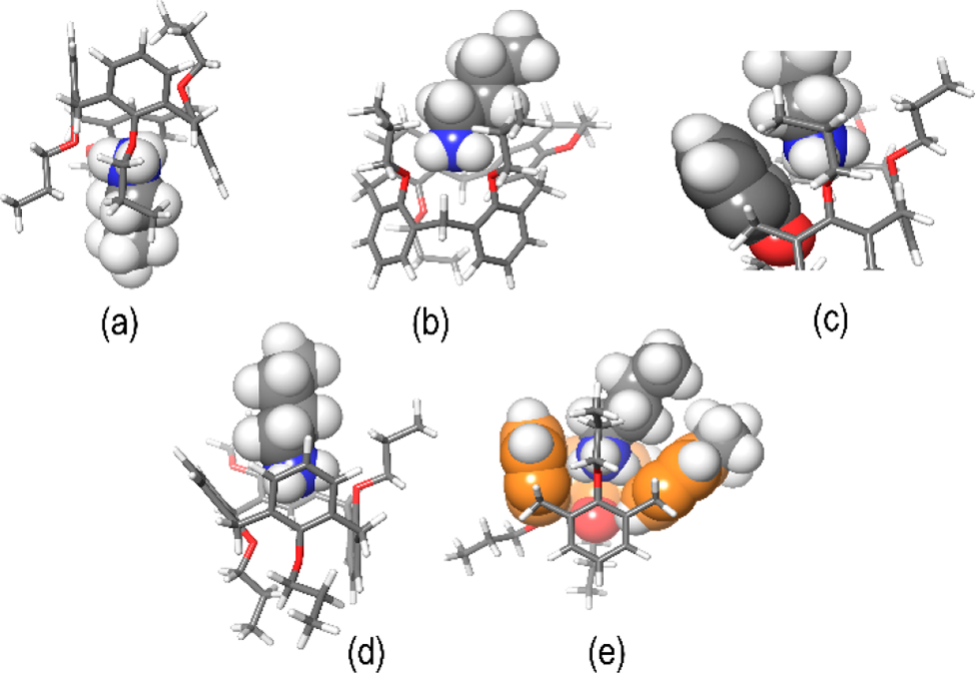
DFT-optimized structures of two stereoisomeric complexes, (a–c) *syn*-OPr and (d and e) *syn*-Ar *n*BuNH_3_^+^@**6**.

On the contrary, the DFT-optimized structure of the *syn*-Ar complex in Figure 4d showed a single H-bonding interaction between the ammonium guest and calix-chromane host **6** (Figure 4d) with a ^+^N–H···O distance of 2.83 Å and a mean ^+^N–H···O angle of 158°. Finally, three cation···π interactions^12^ (Figure 4e) were detected between the ammonium group of the guest and the aromatic rings of the host with a ^+^N···π^centroid^ distance of 3.46 Å.

In conclusion, we have described an example of thermal propargyl Claisen rearrangement starting with monopropargyl-calixarene **1**. The reaction affords unusual calix[3]arene[1]chromene and homocalix[3]arene[1]benzofuran macrocycles due to the molecular rearrangements involving the skeletal ArCH_2_ moiety in addition to the propargyl group. QM DFT calculations indicated that an initial [3,3] sigmatropic reaction affords a highly reactive allene intermediate, which is then stabilized by two main diradical stepwise pathways leading to six- and five-membered oxygenated rings. In the presence of a *n*-butylammonium guest, calix[3]arene[1]chromane **6** forms two stereoisomeric complexes stabilized by ^+^N–H···O H-bonding and cation···π interactions. The calix[3]arene[1]chromene and homocalix[3]arene[1]benzofuran macrocycles described here could pave the way for the synthesis of novel hosts with interesting supramolecular properties.

## Supporting Information Available

Detailed synthetic procedures, 1D and 2D NMR spectra, complexation studies, details of DFT calculations, Cartesian coordinates, X-ray data, and tables of crystal data (PDF)

## Accession Codes

CCDC 1979551 contains the supplementary crystallographic data for this paper. These data can be obtained free of charge via www.ccdc.cam.ac.uk/data_request/cif, or by emailing data_request@ccdc.cam.ac.uk, or by contacting The Cambridge Crystallographic Data Centre, 12 Union Road, Cambridge CB2 1EZ, UK; fax: +44 1223 336033.
