# Supplementary material for: Unusual Calixarenes Incorporating Chromene and Benzofuran Moieties Obtained via Propargyl Claisen Rearrangement
Source: Org Lett. 2021 Nov 15;23(23):9283–7. doi: 10.1021/acs.orglett.1c03643 (PMC8650102; doi:10.1021/acs.orglett.1c03643)
Supplement: Supplementary file 1 — ol1c03643_si_001.pdf [file ol1c03643_si_001.pdf]

Supporting Information

Unusual Calixarenes Incorporating Chromene and Benzofuran Moieties Obtained via Propargyl Claisen Rearrangement

Annunziata Soriente,[,†] Mariantonietta D'Acunto,[†] Carmen Talotta,[†] Carmine Gaeta,[†] Paolo Della Sala,[†] Margherita De Rosa,[†] Silvano Geremia,[‡] Neal Hickey,[‡] Antonio Rescifina,[*,§] and Placido Neri[*,†]*

[†]Dipartimento di Chimica e Biologia "A. Zambelli", Università di Salerno, Via Giovanni Paolo II 132, I-84084 Fisciano (Salerno), Italy
[‡]Centro di Eccellenza in Biocristallografia, Dipartimento di Scienze Chimiche e Farmaceutiche, Università di Trieste, Via L. Giorgieri 1, I-34127 Trieste, Italy
[§]Dipartimento di Scienze del Farmaco e della Salute, Università di Catania, Viale Andrea Doria 6, I-95125 Catania, Italy,

Table of Contents

General Experimental Details S1
The propargyl Claisen rearrangement .... SErrore. Il segnalibro non è definito.
Synthesis of derivative **1** S3
Synthesis of derivatives **2**, **3** and **4** S3
Synthesis of derivative **5** S6
Synthesis of derivative **6** S7
^{1}H and ^{13}C NMR spectra of derivative **1** S8
^{1}H, ^{13}C NMR and 2D NMR spectra of derivative **2** S13
^{1}H, ^{13}C NMR and 2D NMR spectra of derivative **3** S13
^{1}H, ^{13}C NMR and 2D NMR spectra of derivative **4** S16
^{1}H, ^{13}C NMR and 2D NMR spectra of derivative **5** S19
^{1}H, ^{13}C NMR and 2D NMR spectra of derivative **6** S22
Computational details: mechanism investigation S25
Cartesian coordinates of all investigated structures S27
Determination of the crystal structures of **3** S66
DFT calculation S67
Complexation Studies S80
References: S83

General Experimental Details

All chemicals were reagent grade and were used without further purification. Solvents were purchased from Aldrich.[1] Reaction temperatures were measured externally; reactions were monitored by ^{1}H NMR and by TLC on Merck silica gel plates (0.25 mm) and visualized by UV light. Flash chromatography was performed on Merck silica gel (60, 40-63 μm). NMR spectra were recorded on Bruker Avance-600 spectrometer [600.13 MHz (^{1}H) and 150.03 MHz (^{13}C)]; chemical shifts are reported relative to the residual solvent peak ($CHCl_3$: *δ* 7.26, $CDCl_3$: *δ* 77.16). NMR spectra were recorded on a Bruker Avance-600 spectrometer [600 (^{1}H) and 150 MHz (^{13}C)], Bruker Avance-400 spectrometer [400 (^{1}H) and 100 MHz (^{13}C)], and Bruker Avance-300 spectrometer [300 (^{1}H) and 75 MHz (^{13}C)]. Chemical shifts are reported relative to the residual solvent peak ($CHCl_3$: δ 7.26, $CDCl_3$: δ 77.16). Derivative 25,26,27-tripropoxycalix[4]arene **7** was prepared according to the known procedure.[2] Standard pulse programs, provided by the manufacturer, were used for 2D NMR experiments. Structural assignments were made with additional information from correlation spectroscopy (COSY) and heteronuclear single-quantum correlation spectroscopy (HSQC), experiments.

The Propargyl Claisen Rearrangement Performed on Propargyloxy-Tripropoxy-Calix[4]arene 1

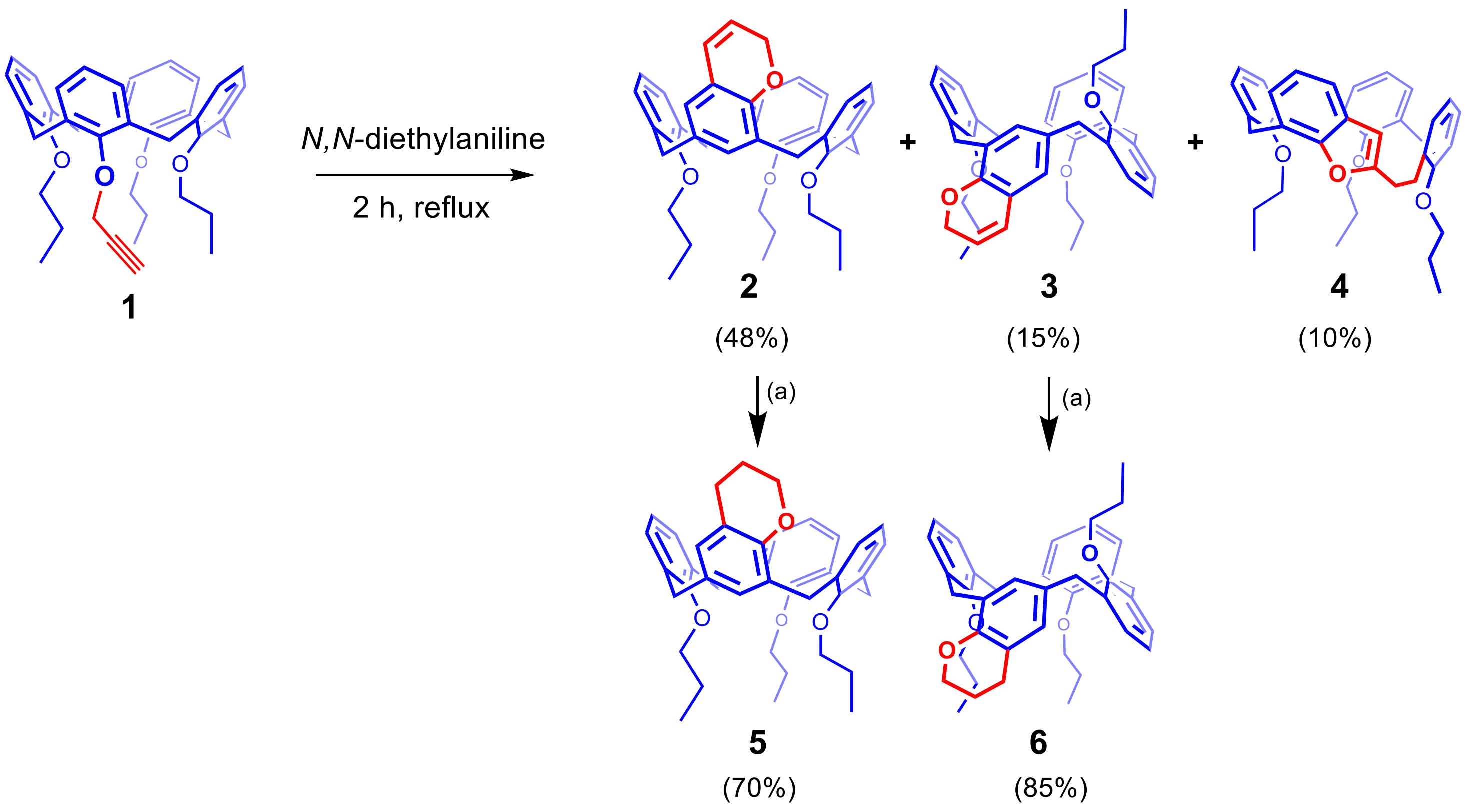

Chart S1. (a) H_2, Pd/C, ethyl acetate, 25 °C, 1 h.

Synthesis of derivative 1

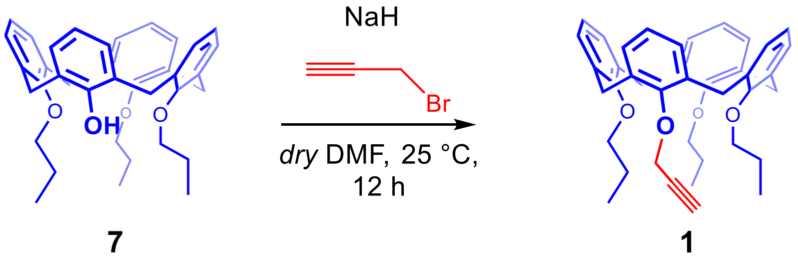

A suspension of 25,26,27-tripropoxycalix[4]arene **7**[2] (1.8 g, 3.0 mmol), NaH (1.12 g, 8.1 mmol), and propargyl bromide (0.8 ml, 7.5 mmol) in dry DMF (100 mL) was stirred for 12 h at room temperature. After evaporation of the solvent the mixture was taken up in CH_2Cl_2 (100 mL) and washed with 1 N HCl (3 X 50 mL) and brine (50 mL). The organic layer was dried with $MgSO_4$, evaporated, and purified by flash chromatography (Petroleum Ether/CH_2Cl_2 7/3); Rf = 0.3 (Petroleum Ether/CH_2Cl_2 85/15). Derivative **1** was obtain as a white solid: 1.36 g (2.31 mmol, 77%); **^{1}H NMR** (600 MHz, $CDCl_3$, 298 K): 7.03 (t, *J* = 7.2 Hz, ArH, 4H), 6.92-6.85 (overlapped, ArH, 2H), 6.34-6.26 (overlapped, ArH, 6H), 4.92 (d, *J* = 2.5 Hz,-OCH_2, 2H), 4.54 (d, $ArCH_2Ar$, *J* = 13.6 Hz, 2H), 4.47 (d, $ArCH_2Ar$, *J* = 13.0 Hz, 2H), 4.02 (t, OCH_2, *J* = 7.6 Hz, 2H), 3.73 (t, -OCH_2, *J* = 6.7 Hz, 4H), 3.19 (d, $ArCH_2Ar$, *J* = 13.6 Hz, 2H), 3.17 (d, $ArCH_2Ar$, *J* = 13.0 Hz, 2H), 2.36 (t, C≡CH, *J* = 2.5 Hz, 1H), 1.99 (m, -OCH_2 CH_2 CH_3, 2H), 1.92 (m, -OCH_2 CH_2 CH_3, 4H), 1.08 (t, *J* = 7.4 Hz,-OCH_2 CH_2 CH_3, 6H), 0.96 (t, *J* = 6.7 Hz, OCH_2 CH_2 CH_3, 3H),**^{13}C NMR** (150 MHz, CDCl3, 298 K): δ 157.81, 155.8, 155.4, 137.8, 136.8, 133.9, 133.6, 129.0, 128.8, 127.8, 127.7, 123.2, 122.3, 122.2, 81.1, 74.6, 59.7, 31.6, 31.1, 23.7, 23.5, 11.0, 10.3. **Anal. Calcd** for $C_{40}H_{44}O_4$: C, 81.60; H, 7.53. Found: C, 81. 68; H, 7.46.

Synthesis of derivatives 2, 3 and 4

A solution of 250 mg (0.42 mmol) of **1** in 5 mL of *N,N*-diethylaniline in an atmosphere of nitrogen, was refluxed for 2.5 h (oil bath). The reaction was cooled to 0 °C and quenched with H_2O (50 mL) and HCl 37 % (50 mL). The mixture was filtered under vacuum and purified by flash chromatography (Petroleum Ether/CH_2Cl_2: 9/1).

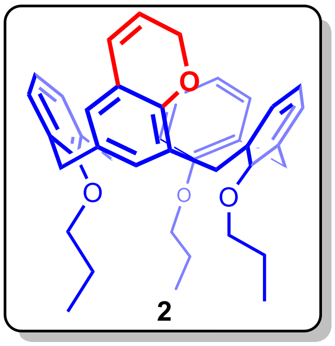

2

Derivative **2**. 120 mg, 0.20 mmol, yield 48 %; Rf = 0.35 (Petroleum Ether/CH_2Cl_2 7/3). **^{1}H NMR** (600 MHz, $CDCl_3$, 298 K): δ 7.20 (d, ArH, *J*= 7.4 Hz, 2H), 7.00 (t, ArH, *J*=7.4 Hz, 1H), 6.73 (d, ArH, *J* = 1.6 Hz, 1H), 6.61 (overlapped, ArH, 2H), 6.47-6.50 (overlapped, ArH + HC=CH, 3H), 6.30 (t, ArH, *J*= 8.1 Hz, 2H), 5.81 (m, HC=CH, 1H), 5.57 (d, ArH, *J* = 1.6 Hz, 1H), 4.85 (br s, OC*H*2CH=CH-, 2H), 4.30 (overlapped, $ArCH_2Ar$, 2H), 4.11 (d, $ArCH_2Ar$, *J*=15.0 Hz, 1H), 4.07 (d, $ArCH_2Ar$, *J*=15.7 Hz, 1H), 3.62-3.65 (overlapped, OC*H*2 CH_2 CH_3, 4H), 3.47-3.58 (overlapped, $ArCH_2Ar$ + OC*H*2 CH_2 CH_3, 4H), 3.19 (d, $ArCH_2Ar$, *J*=13.7 Hz, 2H), 1.77-1.80 (overlapped, OCH_2 C*H*2 CH_3, 4H), 1.60-1.66 (overlapped, OCH_2 C*H*2 C*H*3, 2H), 1.03-1.07 (overlapped, OCH_2 CH_2 C*H*3, 6H), 0.66 (t, OCH_2 CH_2 C*H*3, *J* = 7.5 Hz, 3H). **^{13}C NMR** (150 MHz, $CDCl_3$, 298 K): δ 157.4, 156.0, 155.9, 149.4, 137.8, 134.9, 134.8, 132.1, 132.1, 130.1, 129.5, 129.3, 129.2, 128.7, 128.5, 128.2, 128.1, 125.6, 124.4, 122.9, 122.7, 121.5, 120.9, 76.6, 76.5, 75.7, 65.6, 34.3, 30.9, 30.8, 27.5, 23.8, 23.7, 21.5, 11.0, 10.9, 9.5. **HRMS** (MALDI) m/z $[M]^+$ calcd for $C_{40}H_{44}O_4$: 588.3240. found: 588.3215.

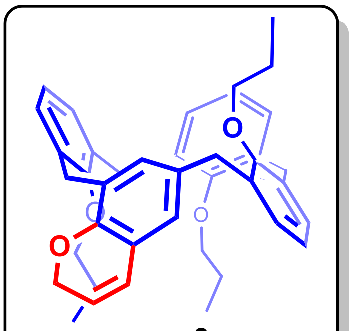

3

Derivative **3.** 37 mg, 0.06 mmol, yield 15 %; Rf = 0.21 (Petroleum Ether/CH_2Cl_2 7/3). **^{1}H NMR** (600 MHz, $CDCl_3$, 298 K): δ 7.16-7.10 (overlapped, ArH, 3H), 6.99-6.97 (overlapped, ArH, 2H), 6.92-6.90 (overlapped, ArH, 3H), 6.79 (dd, ArH, J_1=J_2 = 7.2 Hz, 1H), 6.69 (d, ArH, *J* = 2.1 Hz, 1H), 6.42 (dt, OCH_2CH=C*H*, J_1 = 9.7 Hz, J_2 = 1.8 Hz, 1H), 6.13 (d, ArH, *J* = 2.1 Hz, 1H), 5.77 (dt, OCH_2C*H*=CH, J_1=9.7 Hz, J_2 = 3.5 Hz, 1H), 4.82 (dd, OC*H*2CH=CH-, J_1 = 3.5 Hz, J_2 = 1.8 Hz, 2H), 4.38 and 3.28 (AX, $ArCH_2Ar$, *J* = 12.6 Hz, 2H), 3.98 and 3.92 (AB, $ArCH_2Ar$, *J* = 16.0 Hz, 2H), 3.85 and 3.36 (AB, $ArCH_2Ar$, *J* = 14.9 Hz, 2H), 3.80-3.77 (m, OCH_2, 1H, overlapped to $ArCH_2Ar$ signals), 3.81 and 3.72 (AB, $ArCH_2Ar$, *J*=14.7 Hz, 2H), 3.65-3.47 (overlapped, OC*H*2, 3H), 3.22-3.18 (m, OC*H*2 CH_2 CH_3, 1H), 2.55-2.52 (m, OC*H*2 CH_2 CH_3, 1H), 1.70 (m, OCH_2 C*H*2 CH_3, 2H), 1.59 (m, OCH_2 C*H*2 CH_3, 1H), 0.93-1.06 (overlapped, OCH_2 CH_2 C*H*3 + OCH_2 C*H*2 CH_3, 6H), 0.67 (t, OCH_2 CH_2 C*H*3, *J*=7.2 Hz, 3H), 0.49 (t, OCH_2 CH_2 C*H*3, *J*=7.4 Hz, 3H), **^{13}C NMR** (150 MHz, $CDCl_3$, 298 K): δ 156.8, 156.4,

156.0, 135.7, 134.8, 134.8, 133.8, 133.8, 133.6, 130.5, 129.8, 129.6, 129.5, 129.3, 128.8, 125.5, 124.2, 123.6, 123.0, 122.9, 121.5, 76.5, 74.9, 73.3, 65.6, 38.3, 34.7, 30.5, 29.8, 28.3, 23.6, 23.1, 22.3, 10.8, 10.6, 9.8. **Anal. Calcd** for $C_{40}H_{44}O_4$: C, 81.60; H, 7.53. Found: C, 81.69; H, 7.44.

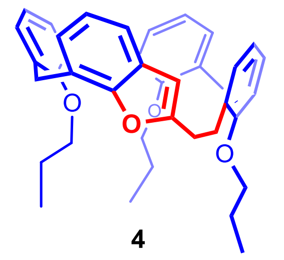

4

Derivative **4.** 25 mg, 0.04 mmol, yield 10 %; Rf = 0.4 (Petroleum Ether/CH_2Cl_2 7/3). **^{1}H NMR** (600 MHz, $CDCl_3$, 298 K): δ 7.42 (d, ArH, *J*=7.8 Hz, 1H), 7.24 (d, ArH, *J*=7.5 Hz, 1H), 7.16 (overlapped, ArH, 2H), 7.08 (d, ArH, *J*=7.3 Hz, 1H), 7.01 (t, ArH, *J*=7.2 Hz, 1H), 6.51 (s, C=CH, 1H), 6.41 (t, ArH, *J*=7.6 Hz, 1H), 6.30 (d, ArH, *J*=7.5, 1H), 6.15 (d, ArH, *J*=7.4 Hz, 1H), 6.06-6.11 (overlapped, ArH, 2H), 5.86 (d, ArH, *J*=7.6 Hz, 1H), 4.65 (d, ArCH$_2$Ar, *J*=15.9 Hz, 1H), 4.45 (d, ArCH$_2$Ar, *J*=13.7 Hz, 1H), 4.36 (d, ArCH$_2$Ar, *J*=14.8 Hz, 1H), 3.58-3.85 (overlapped, ArCH$_2$Ar + OC*H*$_2$ CH$_2$ CH$_3$, 7H), 3.22-3.29 (overlapped, CH$_2$ + ArCH$_2$Ar, 3H), 3.13 (m, CH$_2$, 1H), 2.79 (m,CH$_2$, 1H), 2.44 (m,CH$_2$, 1H), 1.82-1.89 (overlapped, OCH$_2$ C*H*$_2$ CH$_3$, 6H), 1.12 (overlapped, OCH$_2$ CH$_2$ C*H*$_3$, 6H), 0.96 (t, OCH$_2$ CH$_2$ C*H*$_3$, *J*=7.6 Hz, 3H). **^{13}C NMR** (150 MHz, $CDCl_3$, 298 K): δ 160.7, 157.6, 155.0, 154.4, 152.9, 137.8, 136.4, 135.3, 135.1, 134.4, 132.2, 130.1, 129.8, 129.4, 127.5, 127.3, 126.9, 125. 6, 125.2, 123.5, 123.1, 122.8, 122.6, 122.2, 118.4, 102.2, 75.2, 74.9, 74.5, 32.5, 31.2, 30.8, 29.9, 29.4, 23.9, 23.89, 23.0, 11.1, 10.9, 10.4. **Anal. Calcd** for $C_{40}H_{44}O_4$: C, 81.60; H, 7.53. Found: C, 81.70; H, 7.44.

Synthesis of derivative 5

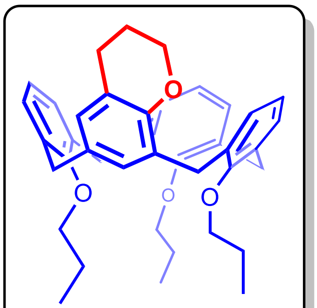

5

A solution of **2** (80 mg, 0.14 mmol) in ethyl acetate (2 mL) was added to Pd/C (20 mg) and stirred for 1 h under hydrogen atmosphere at room temperature. Then the catalyst was removed through a short plug of celite and the solvent was evaporated to give a light brown row. The crude product was purified through chromatographic column on silica gel (petroleum ether/ CH_2Cl_2= 9:1, v/v) to give the pure product **5** (58 mg, 70 %) as a white solid; Rf = 0.3 (Petroleum Ether/CH_2Cl_2 7/3). **^{1}H NMR** (600 MHz, $CDCl_3$, 298 K): δ 7.20 (d, ArH, *J*=7.3 Hz, 2H), 7.00 (t, ArH, *J*=7.5 Hz, 1H), 6.80 (s, ArH, 1H), 6.59 (d, ArH, *J*=7.4 Hz, 2H), 6.48-6.51 (overlapped, ArH, 2H), 6.30 (overlapped, ArH, 2H), 5.51 (s, ArH, 1H), 4.26-4.33 (overlapped, OC*H*$_2$ CH$_2$ CH$_3$+ArCH$_2$Ar, 4H), 4.15 (d, ArCH$_2$Ar, *J*=15.2 Hz, 1H), 4.03 (d, ArCH$_2$Ar, *J*=15.5 Hz, 1H), 3.54-3.65 (overlapped, OC*H*$_2$ CH$_2$ +ArCH$_2$Ar, 7H), 3.49 (d, ArCH$_2$Ar, *J*=15.1 Hz, 1H), 3.18-3.21 (overlapped, ArCH$_2$Ar, 2H), 2.85 (t, CH$_2$ C*H*$_2$, *J*=6.5 Hz, 2H), 2.06 (broad, CH$_2$ C*H*$_2$, 2H), 1.67-1.80 (overlapped, OCH$_2$ C*H*$_2$ CH$_3$, 6H), 1.03-1.07 (overlapped, OCH$_2$ CH$_2$ C*H*$_3$, 6H), 0.68 (t, OCH$_2$ CH$_2$ C*H*$_3$, *J*=7.5 Hz, 3H). **^{13}C NMR** (150 MHz, $CDCl_3$, 298 K): δ 157.4, 156.0, 156.0, 150.3, 137.9, 134.7, 134.7, 133.5, 132.5, 132.3, 130.4,129.2, 129.1, 128.8, 128.5, 128.1, 128.0, 128.0, 127.2, 122.8, 122.6, 120.0,76.6, 75.8, 66.7, 34.1, 30.9, 30.8, 27.5, 27.0, 25.32, 23.8, 23.7, 22.9, 21.4, 11.0, 10.9, 9.5. **Anal. Calcd** for $C_{40}H_{46}O_4$: C, 81.32; H, 7.85. Found: C, 81.41; H, 7.75.

Synthesis of derivative 6

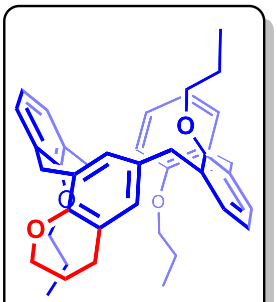

6

A solution of **5** (50 mg, 0.08 mmol) in ethyl acetate (1 ml) was added to Pd/C (20 mg) and stirred for 1 h under hydrogen atmosphere at room temperature. Then the catalyst was removed through a short plug of celite and the solvent was evaporated to give a light brown row. The crude product was purified through chromatographic column on silica gel (petroleum ether/ CH_2Cl_2= 9:1, v/v) to give the pure product **6** (40 mg, 85 %) as a white solid; Rf = 0.19 (Petroleum Ether/CH_2Cl_2 7/3).**^{1}H NMR** (600 MHz, $CDCl_3$, 298 K): δ 7.19 (d, ArH, *J*=7.6 Hz, 1H), 7.11 (t, ArH, *J*=6.5 Hz, 2H), 6.88-6.99 (overlapped, ArH, 5H), 6.79 (t, ArH, *J*=7.2 Hz, 1H), 6.75 (s, ArH, 1H), 6.05 (s, ArH, 1H), 4.41 (d, $ArCH_2Ar$, *J*=12.6 Hz, 1H), 4.23 (t, OCH_2, *J*=4.8 Hz, 2H), 4.00 (d, $ArCH_2Ar$, *J*=16.4 Hz, 1H), 3.95 (d, $ArCH_2Ar$, *J*=16.6 Hz, 1H), 3.73-3.87 (overlapped, OCH_2 + $ArCH_2Ar$, 4H), 3.52-3.60 (overlapped, OCH_2, 2H), 3.44-3.48 (m, OCH_2, 1H), 3.29-3.33 (overlapped, $ArCH_2Ar$, 2H), 3.24 (m, OCH_2, 1H), 2.79 (m, CH_2, 2H), 2.60 (m, OCH_2, 1H), 2.01 (m, CH_2, 2H), 1.71(m, OCH_2 C*H*$_2$ CH_3, 2H), 1.59 (m, OCH_2 C*H*$_2$ CH_3,, 1H), 0.94-1.05 (overlapped, OCH_2 CH_2 C*H*$_3$ + OCH_2 C*H*$_2$ CH_3, 6H), 0.65 (t, OCH_2 CH_2 C*H*$_3$, *J*=7.6 Hz, 3H), 0.50 (t, OCH_2 CH_2 C*H*$_3$, *J*=7.3 Hz, 3H). **^{13}C NMR** (150 MHz, $CDCl_3$, 298 K): δ 156.8, 156.2, 156.1, 150.4, 135.9, 134.5, 134.3, 134.0, 133.6, 133.5, 130.7, 129.7, 129.5, 129.4, 129.1, 128.8, 128.7, 128.3, 127.0, 123.5, 123.0, 122.8, 120.3, 76.6, 74.7, 73.2, 66.6, 38.4, 34.7, 30.5, 28.4, 25.2, 23.59, 23.0, 22.8, 22.1, 10.7, 10.6, 9.7.

Anal. Calcd for $C_{40}H_{46}O_4$: C, 81.32; H, 7.85. Found: C, 81.40; H, 7.76.

^{1}H and ^{13}C NMR spectra of derivative 1

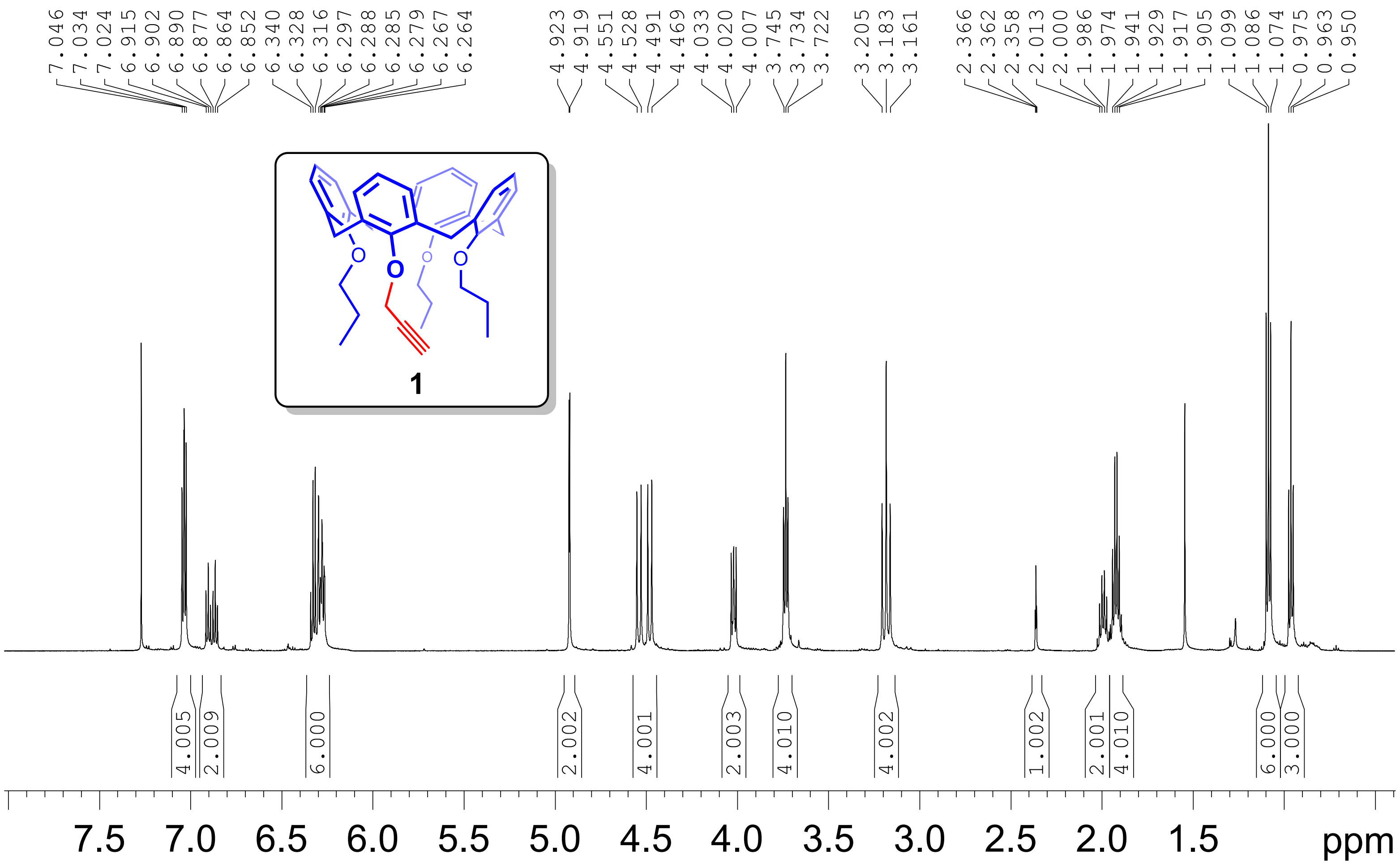

Figure S1. ^{1}H NMR spectrum of derivative **1** (600 MHz, $CDCl_3$, 298 K).

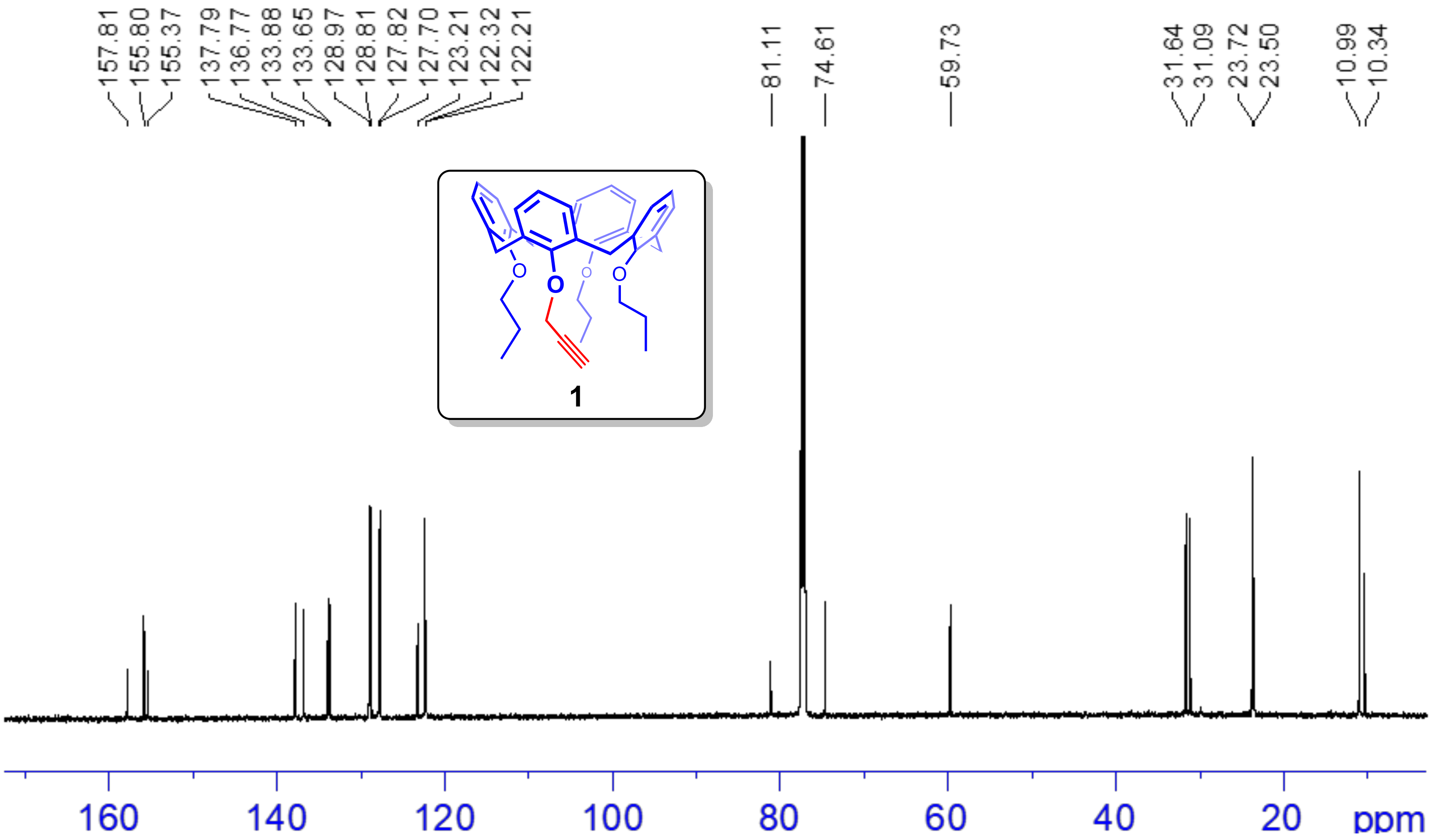

Figure S2. ^{13}C NMR spectrum of derivative **1** (150 MHz, $CDCl_3$, 298 K).

^{1}H, ^{13}C NMR and 2D NMR spectra of derivative 2

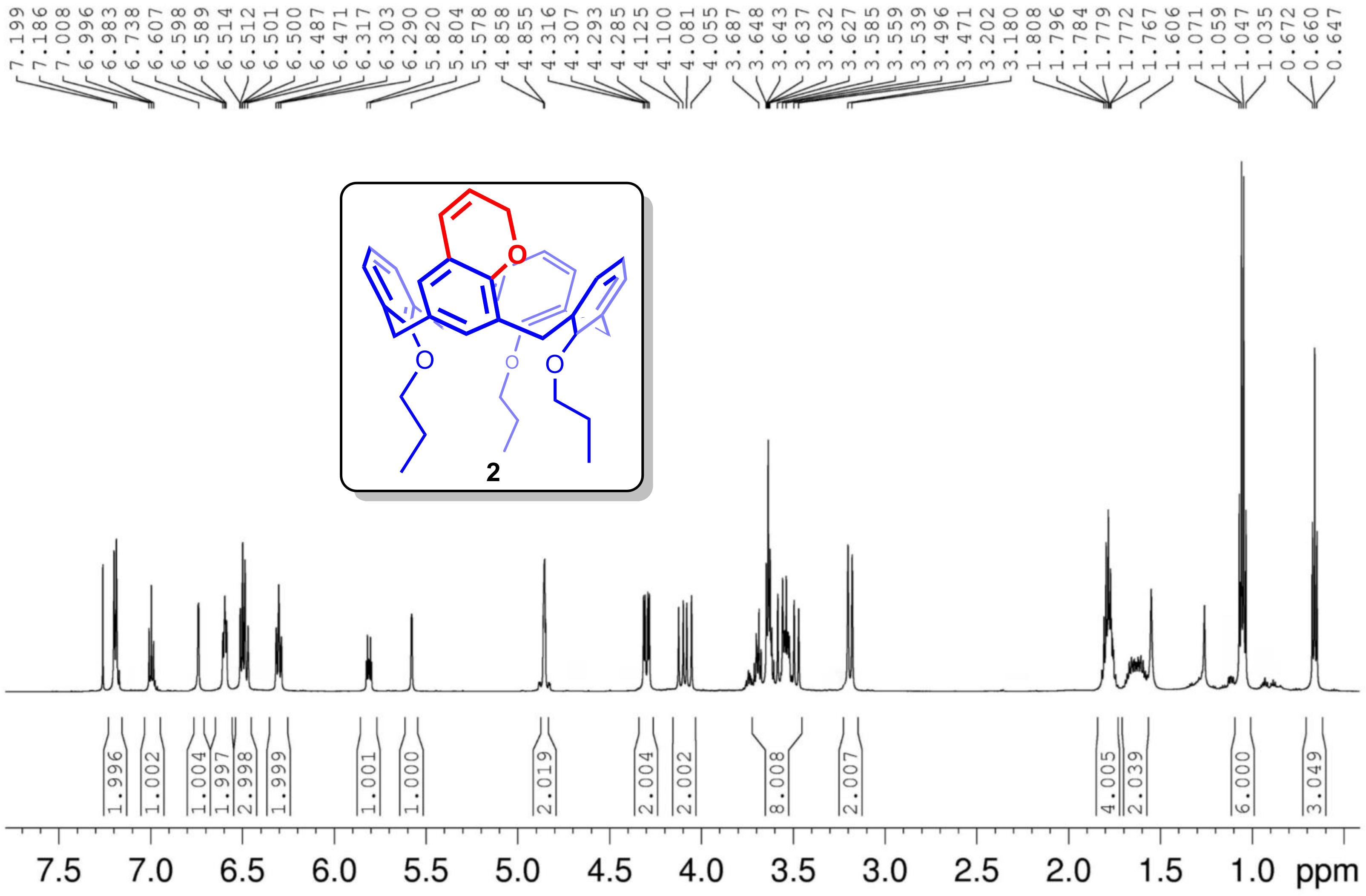

Figure S3. ^{1}H NMR spectrum of **2** ($CDCl_3$, 600 MHz, 298 K).

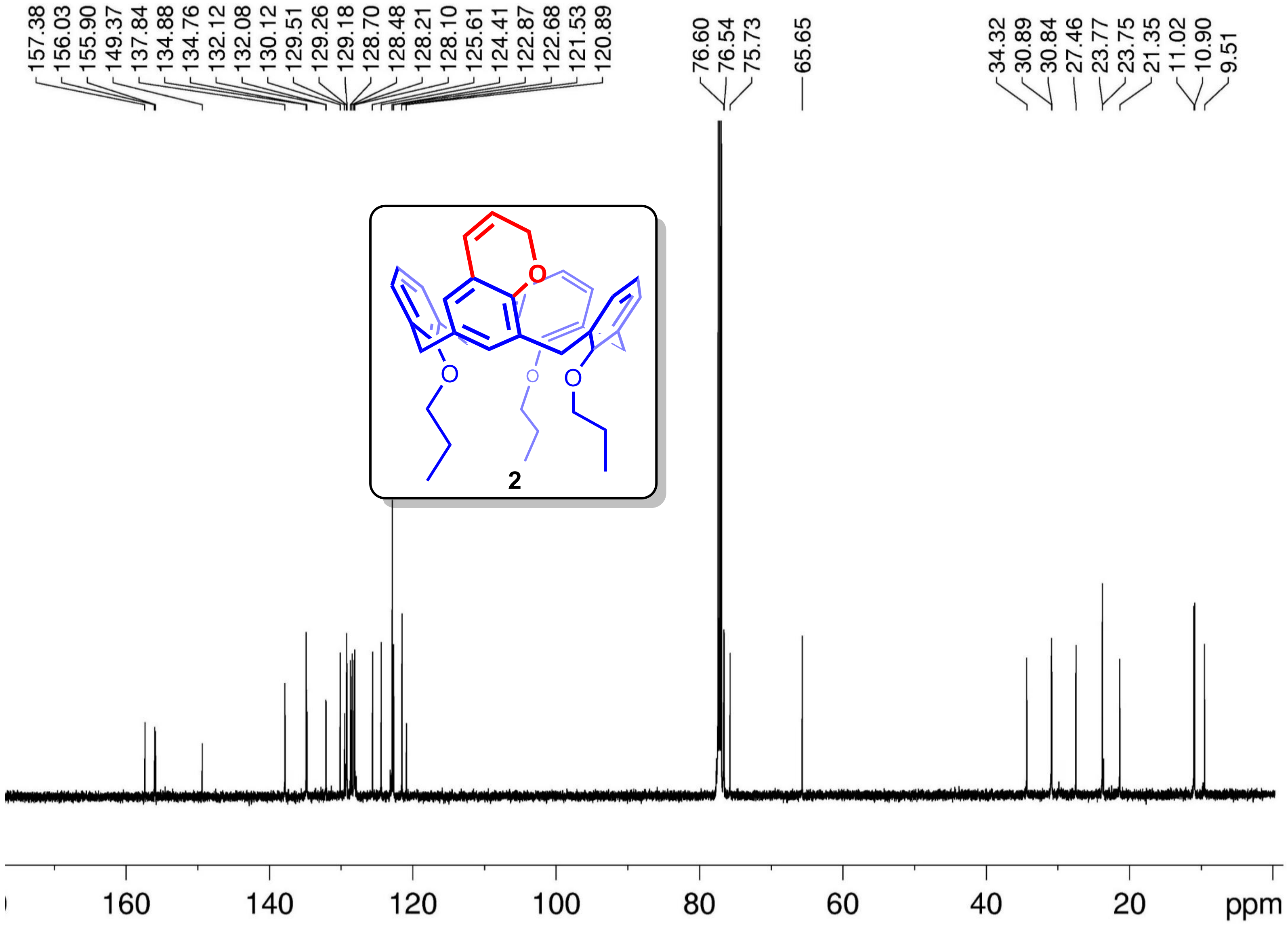

Figure S4. ^{13}C NMR spectrum of **2** ($CDCl_3$, 150 MHz, 298 K).

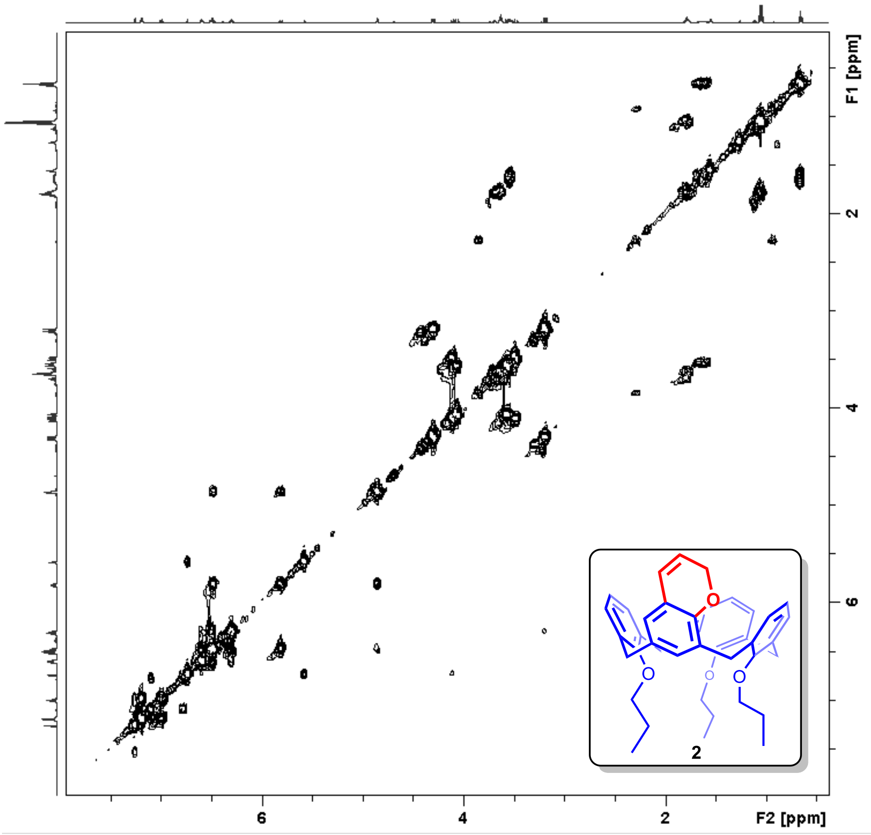

Figure S5. DQF COSY spectrum of **2** ($CDCl_3$, 600 MHz, 298 K).

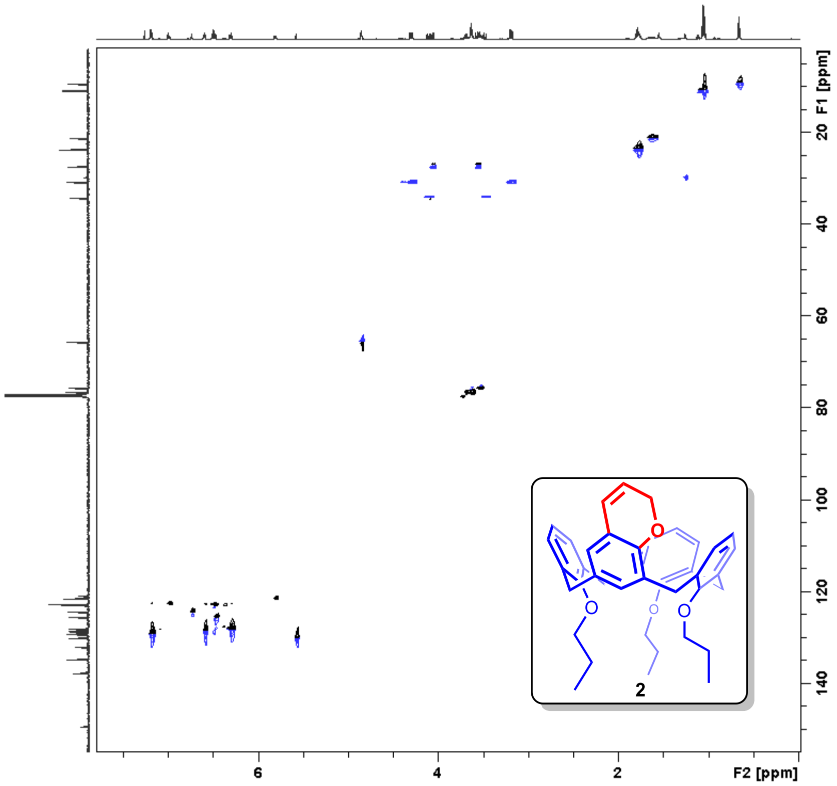

Figure S6. 2D-HSQC spectrum of **2** ($CDCl_3$, 600 MHz, 298 K).

^{1}H, ^{13}C NMR and 2D NMR spectra of derivative 3

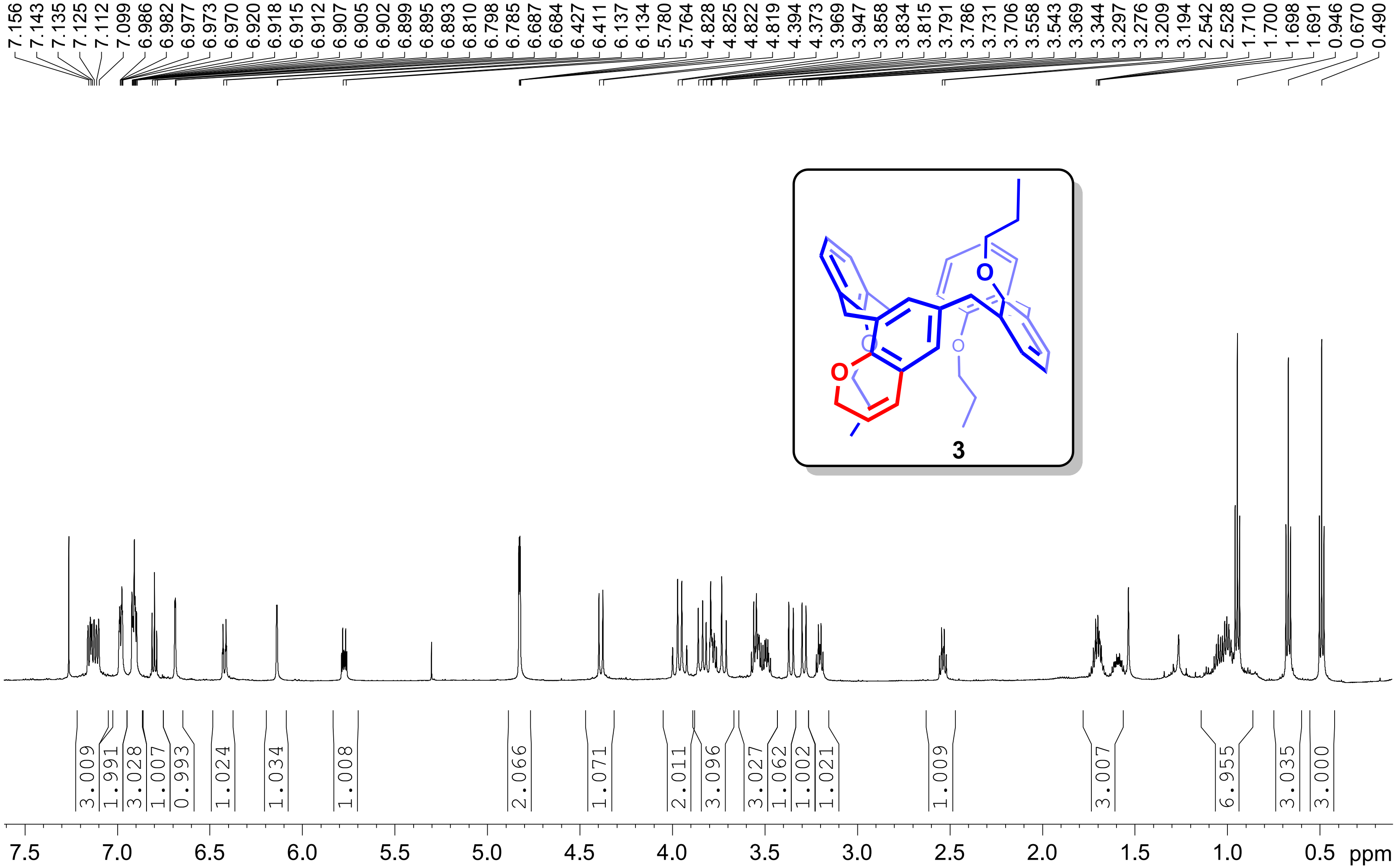

Figure S7. ^{1}H NMR spectrum of **3** ($CDCl_3$, 600 MHz, 298 K)

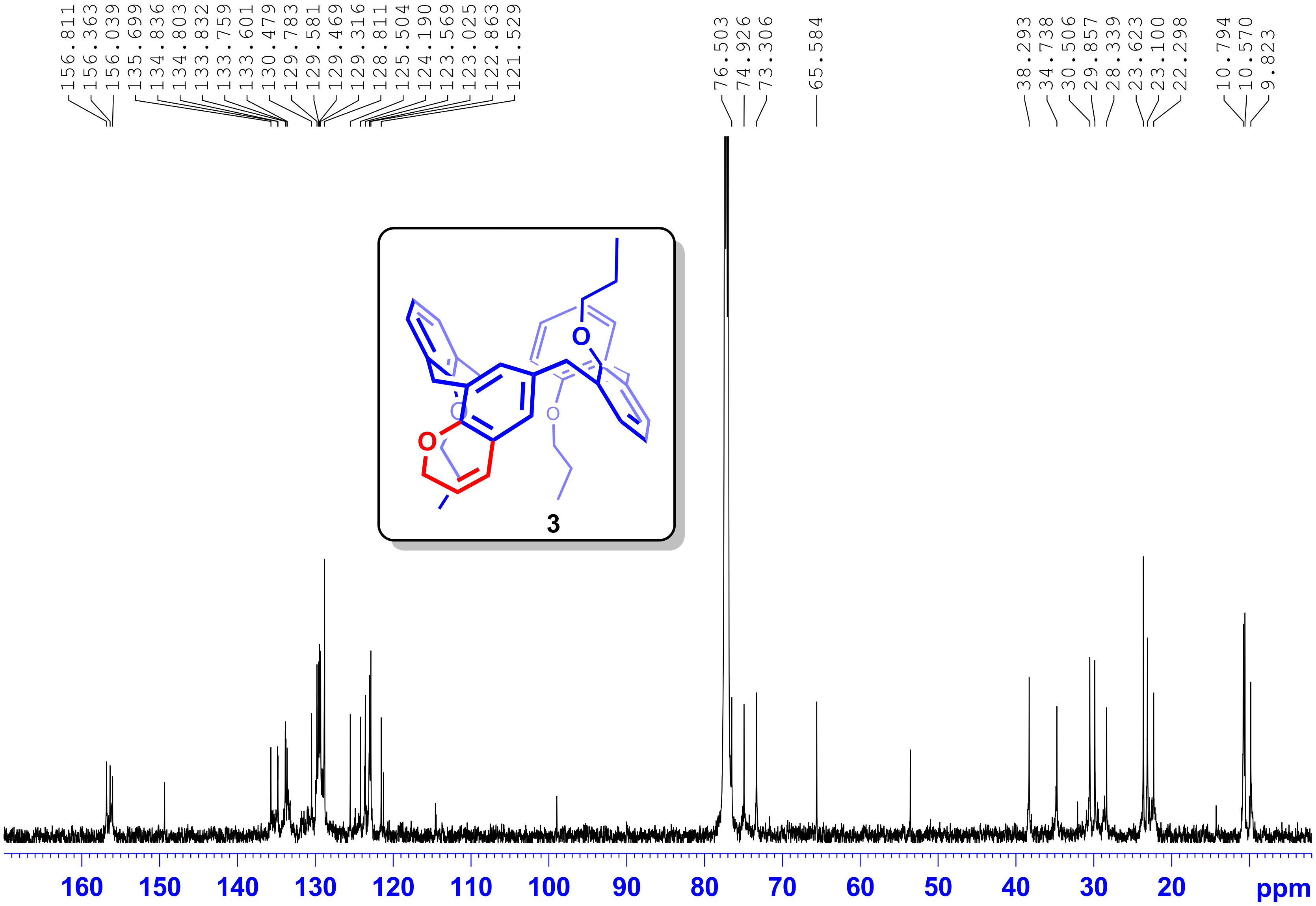

Figure S8. ^{13}C NMR spectrum of **3** ($CDCl_3$, 150 MHz, 298 K).

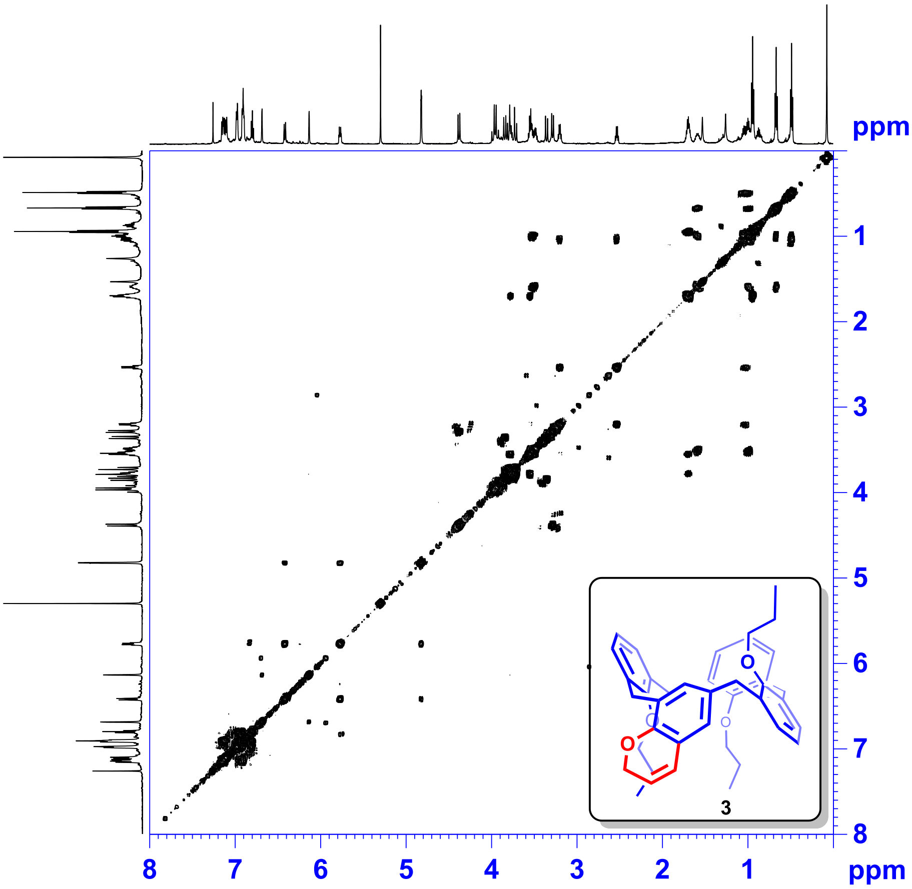

Figure S9. DQF COSY spectrum of **3** ($CDCl_3$, 600 MHz, 298 K).

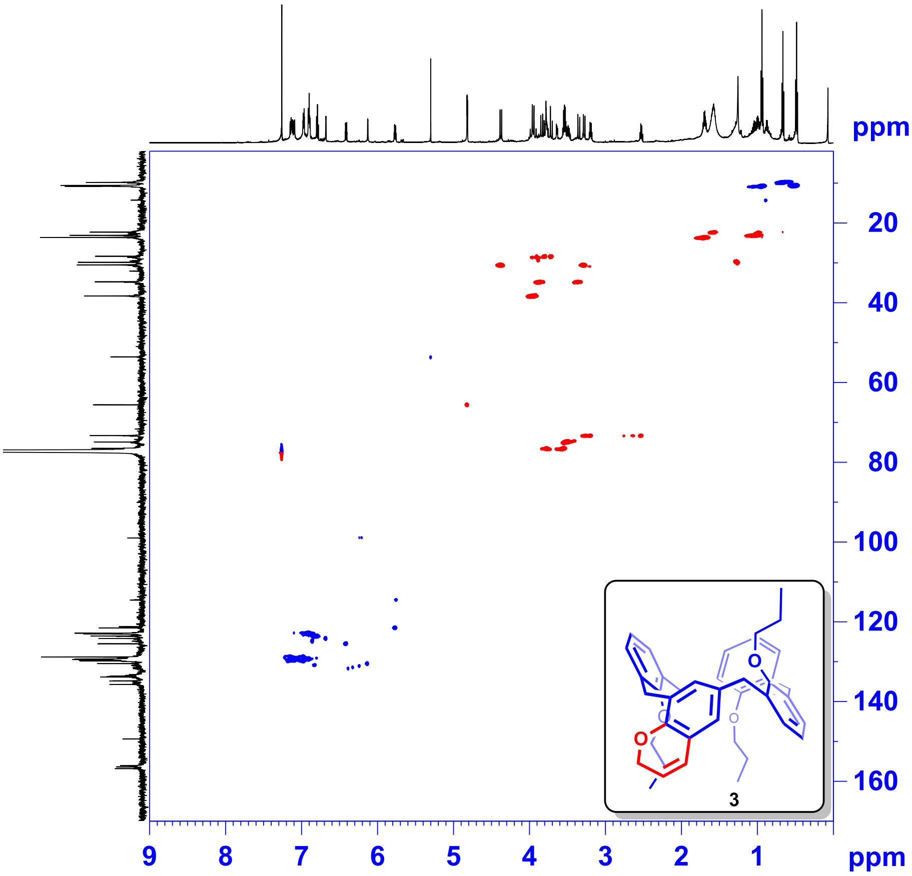

Figure S10. 2D-HSQC spectrum of **3** ($CDCl_3$, 600 MHz, 298 K).

^{1}H, ^{13}C NMR and 2D NMR spectra of derivative 4

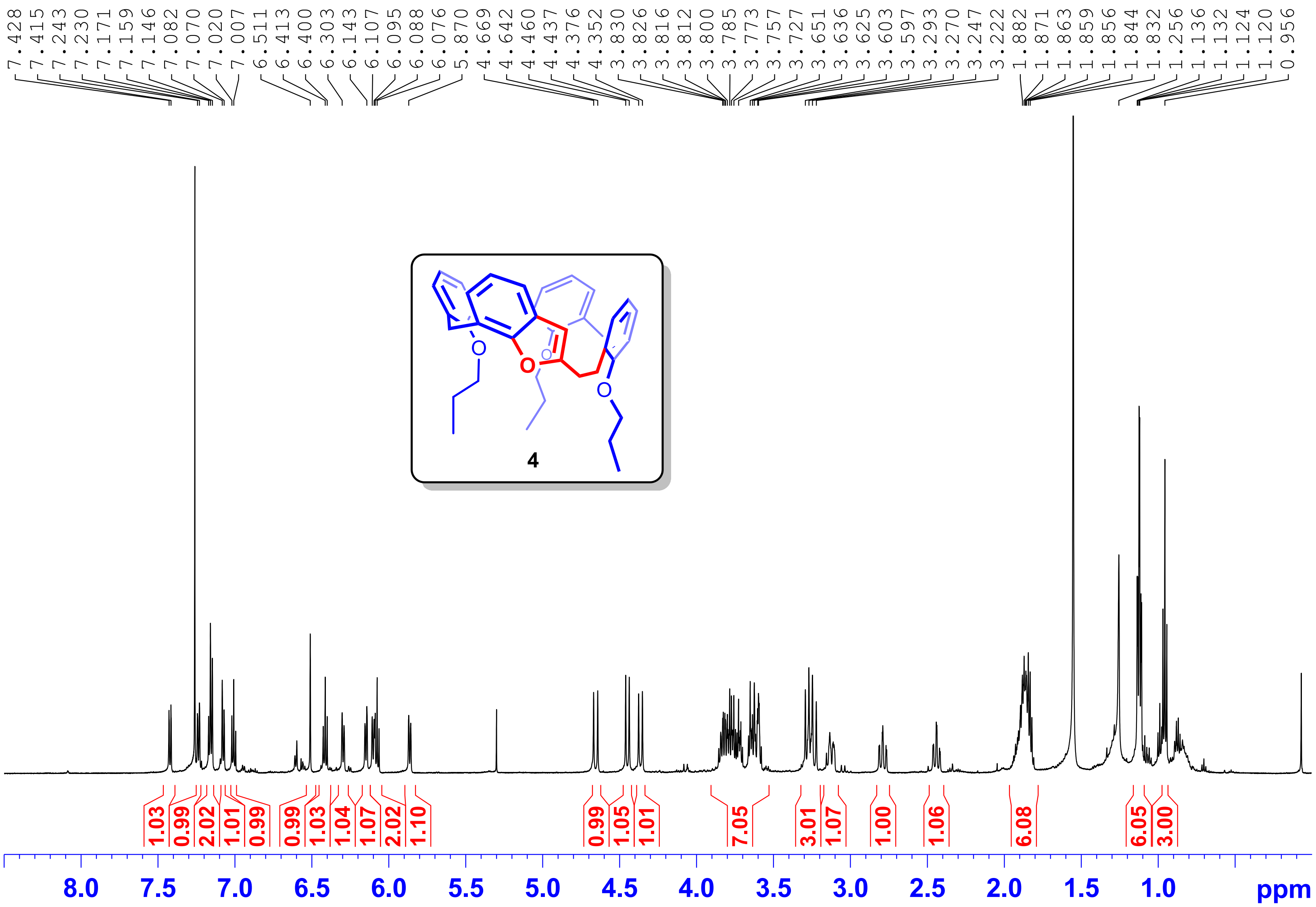

Figure S11. ^{1}H NMR spectrum of **4** ($CDCl_3$, 600 MHz, 298 K).

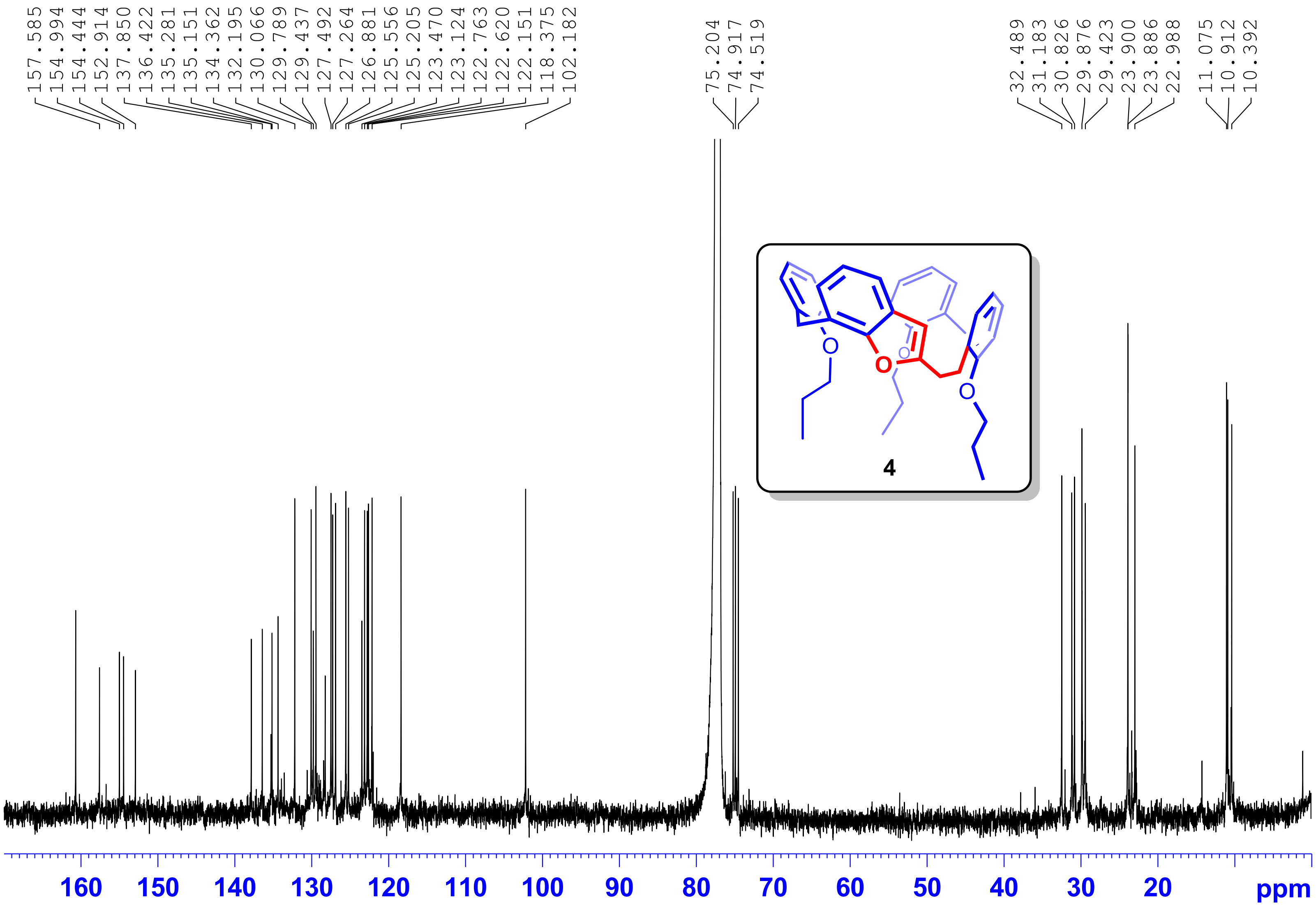

Figure S12. ^{13}C NMR spectrum of **4** ($CDCl_3$, 150 MHz, 298 K).

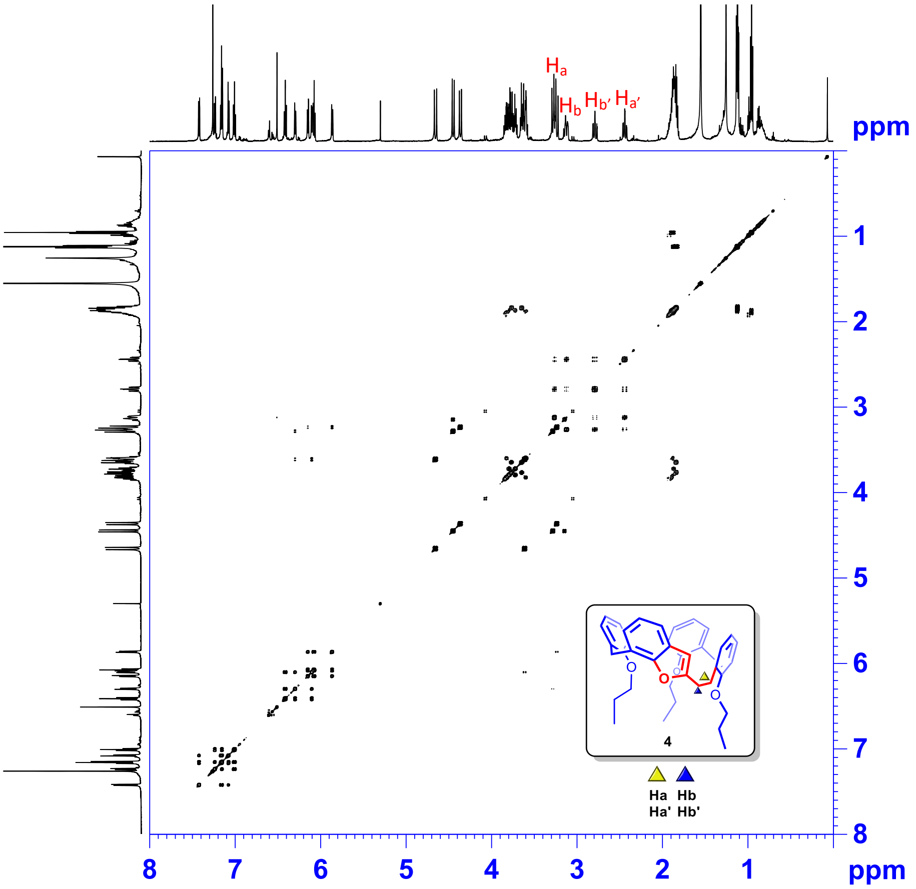

Figure S13. DQF COSY spectrum of **4** ($CDCl_3$, 600 MHz, 298 K).

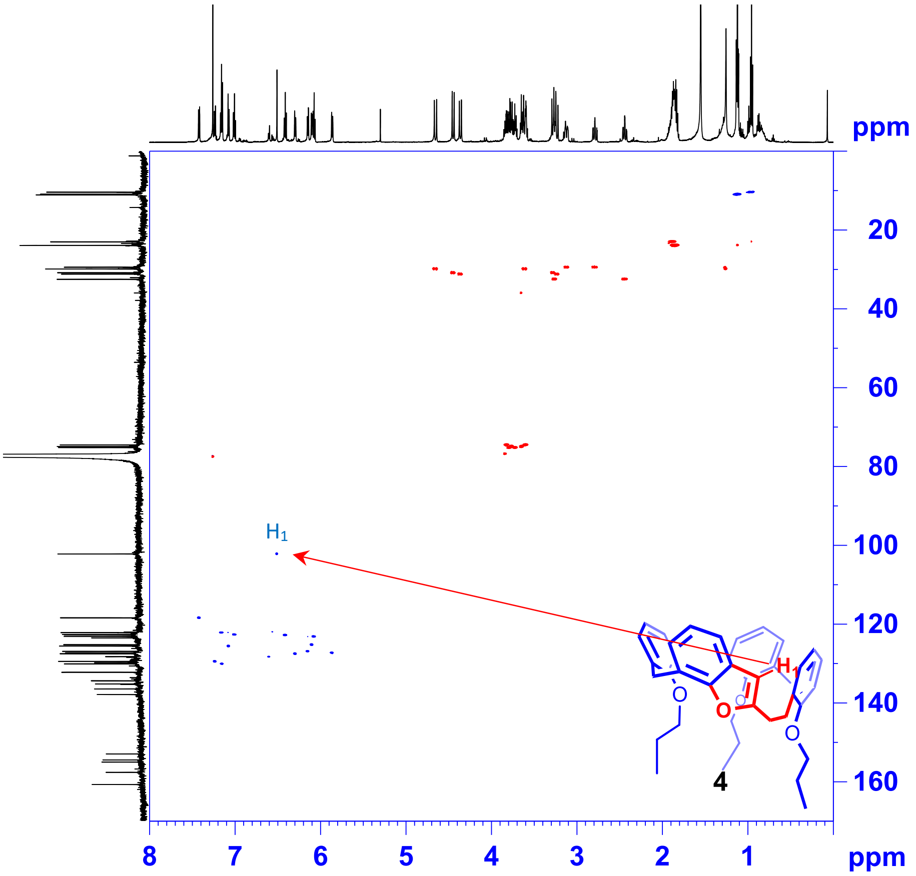

Figure S14. 2D-HSQC spectrum of **4** ($CDCl_3$, 600 MHz, 298 K).

^{1}H, ^{13}C NMR and 2D NMR spectra of derivative 5

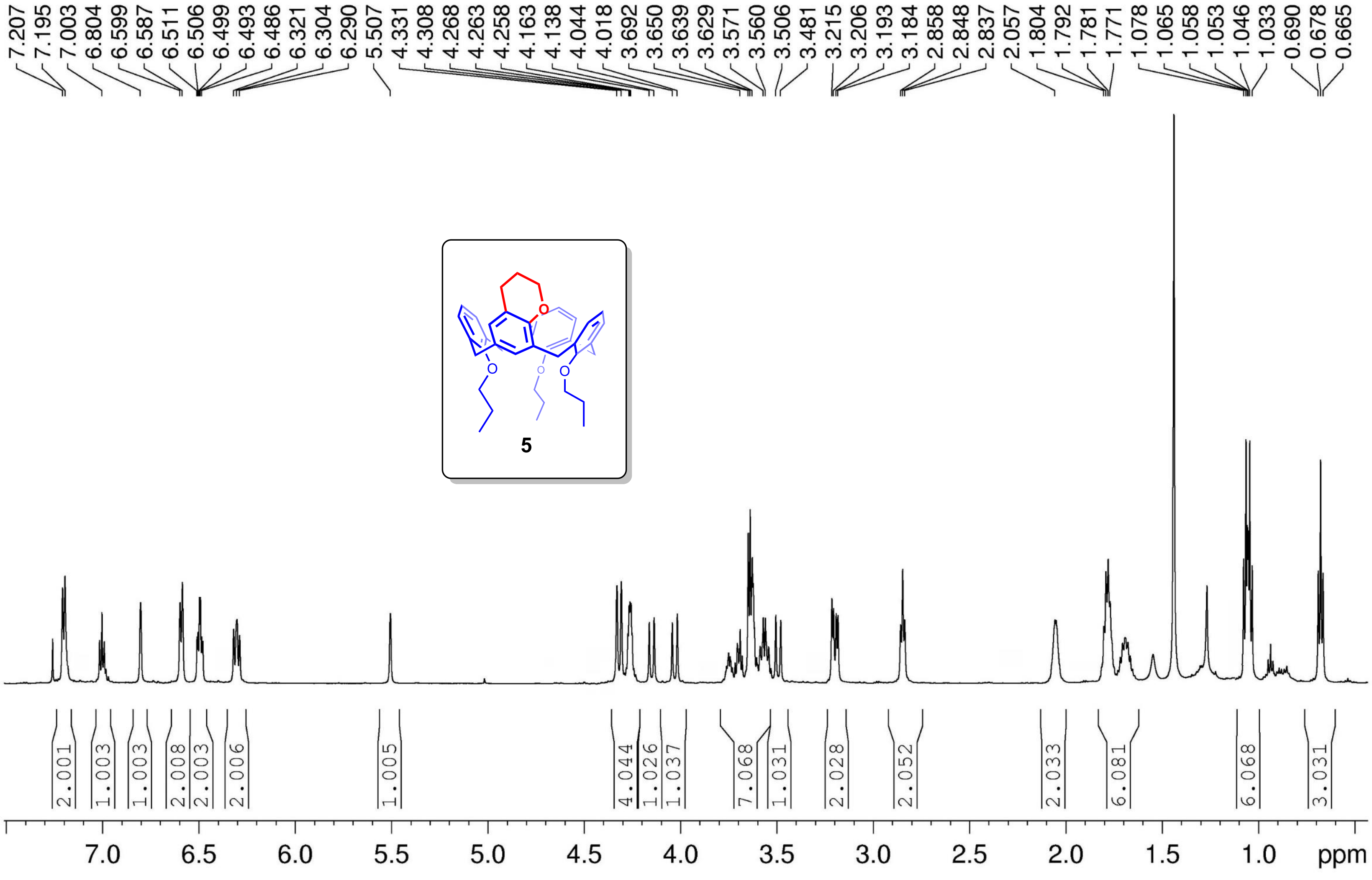

Figure S15. ^{1}H NMR spectrum of **5** ($CDCl_3$, 600 MHz, 298 K).

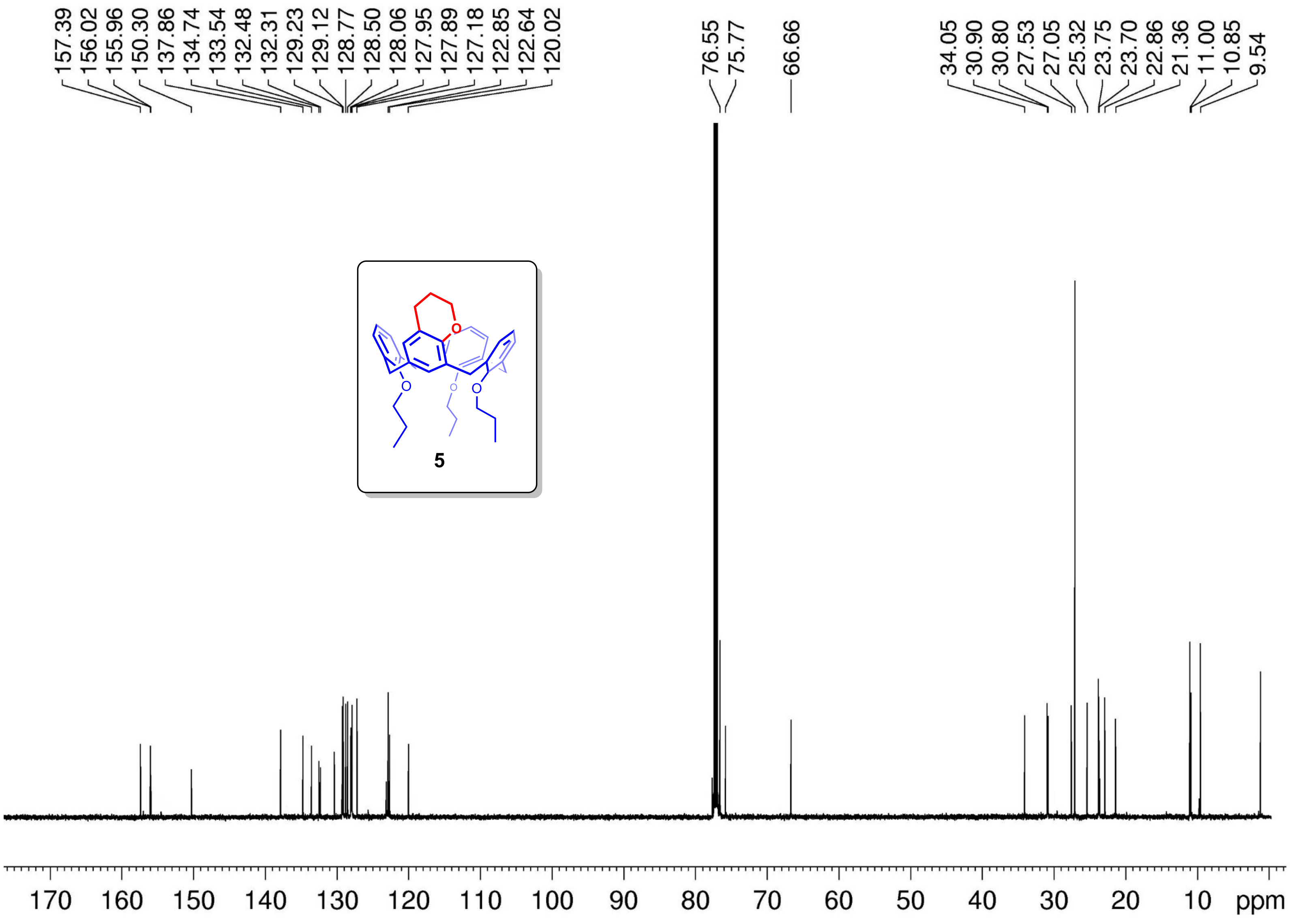

Figure S16. ^{13}C NMR spectrum of **5** ($CDCl_3$, 150 MHz, 298 K).

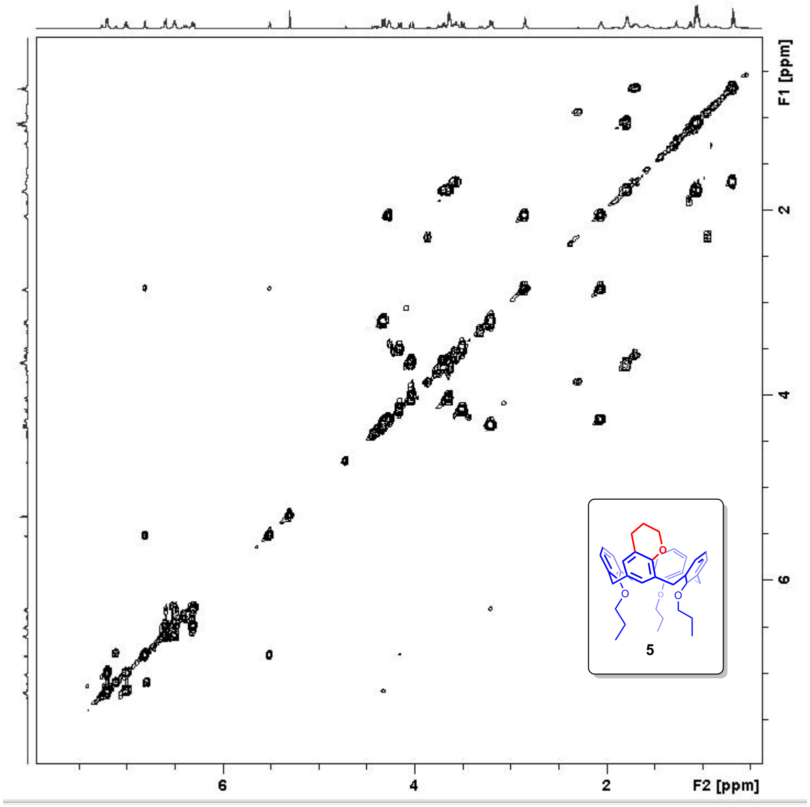

Figure S17. DQF COSY spectrum of **5** ($CDCl_3$, 600 MHz, 298 K).

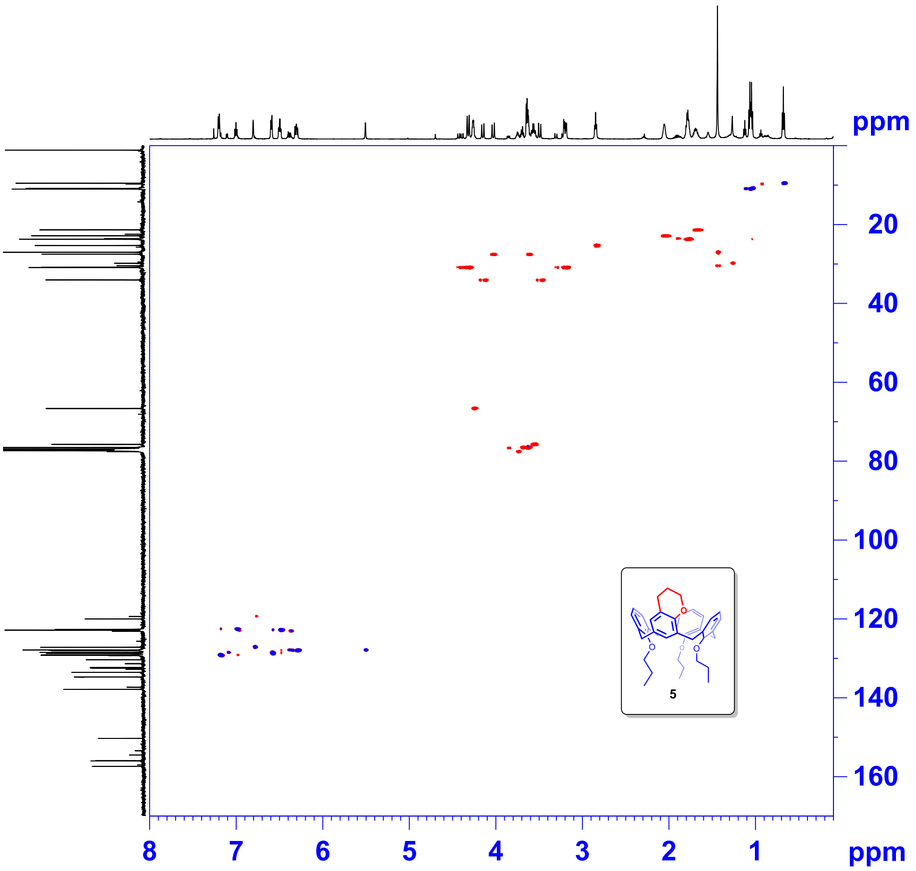

Figure S18. 2D-HSQC spectrum of **5** ($CDCl_3$, 600 MHz, 298 K).

^{1}H, ^{13}C NMR and 2D NMR spectra of derivative 6

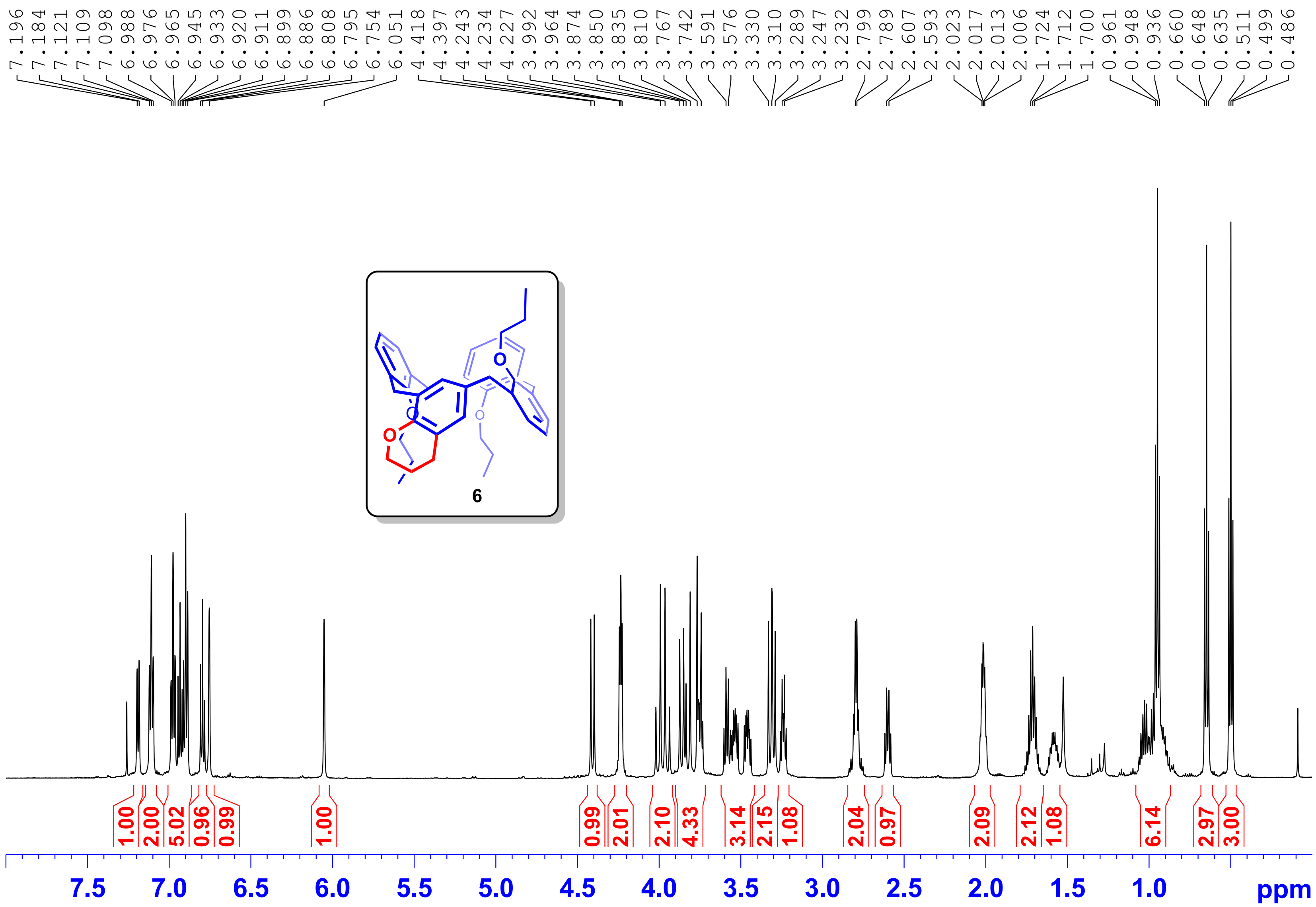

Figure S19. ^{1}H NMR spectrum of **6** ($CDCl_3$, 600 MHz, 298 K).

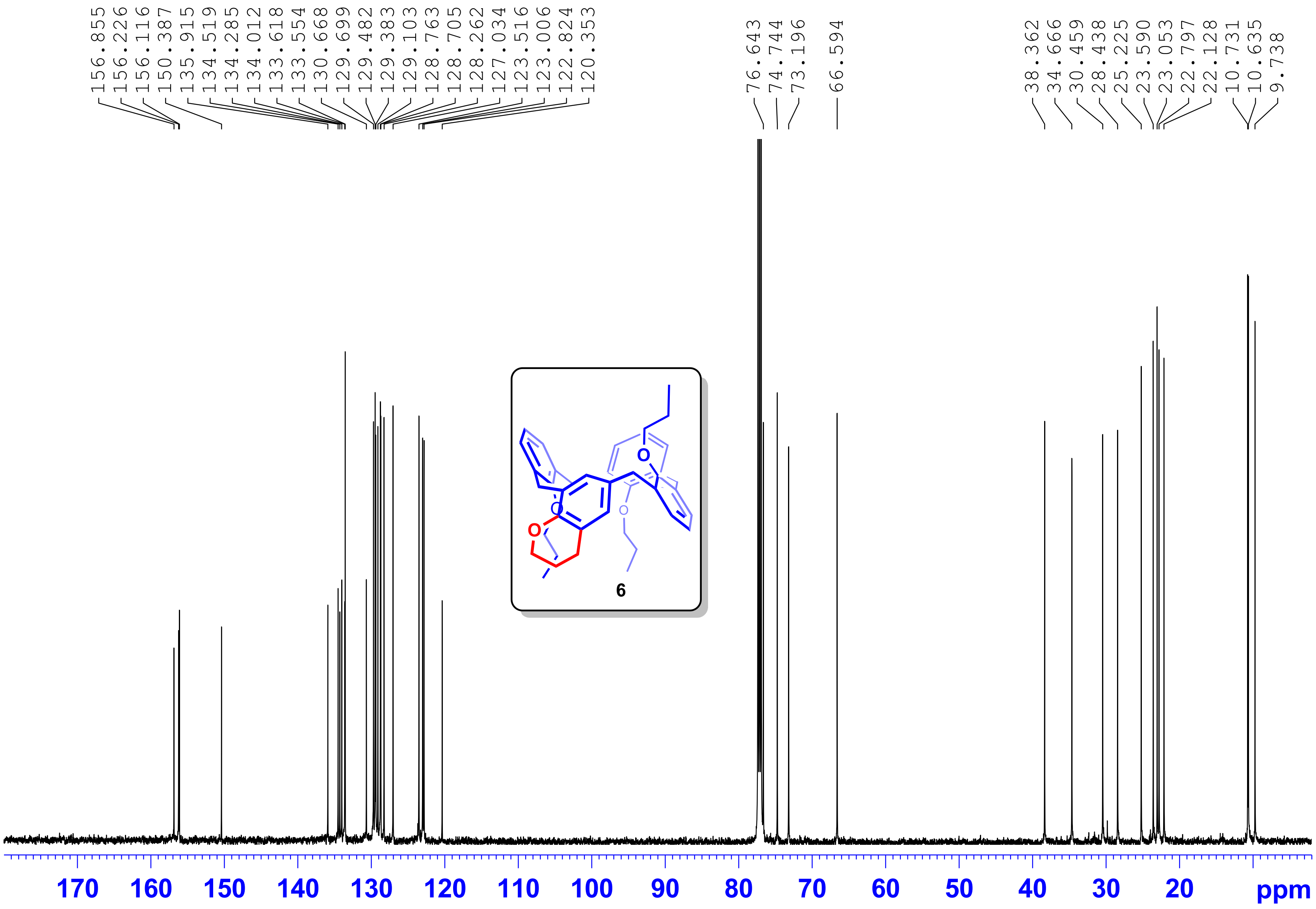

Figure S20. ^{13}C NMR spectrum of **6** ($CDCl_3$, 150 MHz, 298 K).

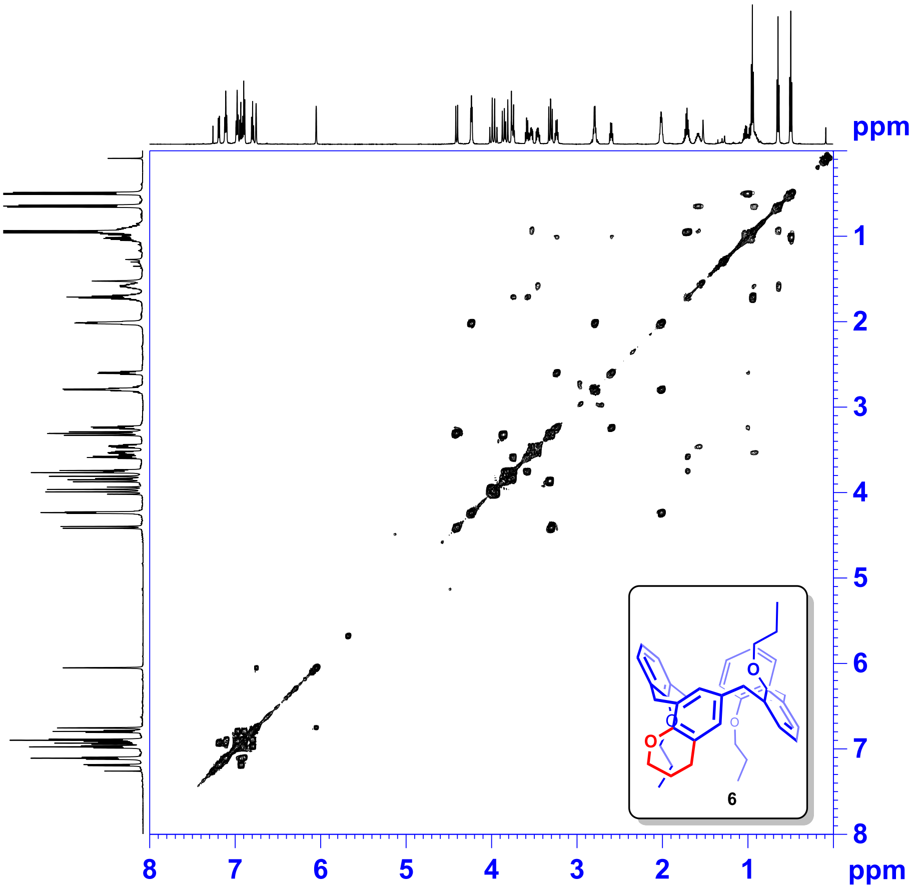

Figure S21. DQF COSY spectrum of **6** ($CDCl_3$, 600 MHz, 298 K).

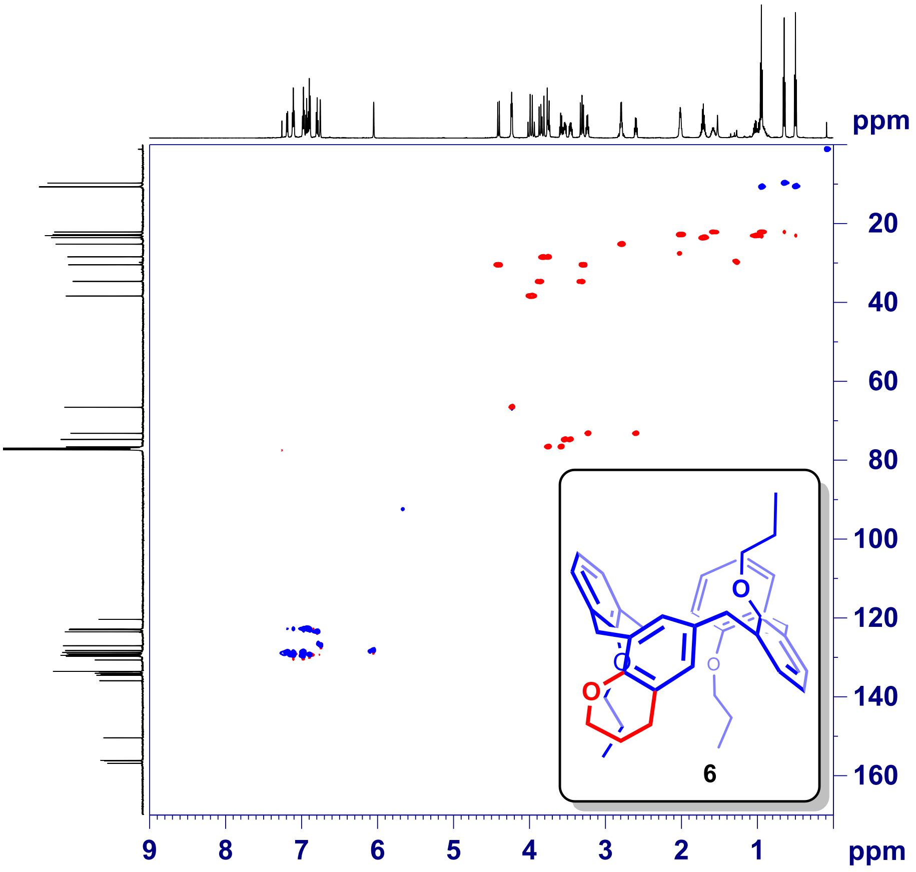

Figure S22. 2D-HSQC spectrum of **6** ($CDCl_3$, 600 MHz, 298 K).

Computational details: mechanism investigation (figure 3)

Geometry optimizations have been performed with (U)B3LYP functional using 6-31+G* the basis set. The same level of method was used for the frequency calculations at all the optimized structures. Zero-point vibrational energy corrections are included in the total energy. Harmonic frequency calculations were performed for all stationary points to confirm them as minima. The intrinsic reaction coordinate (IRC) calculations have also been carried out to verify the identity of the transition state (TS) structures and to obtain the potential energy surface profile connecting the TS to the two associated minima of the proposed mechanisms. All calculations have been performed with the Gaussian 16 package.[3]

Table S1. Energies in Hartree and Number of Negative Frequencies for Structures, Intermediates, and Transition States of the Investigated Rearrangement

Structure	E(0)	E	H	G	Neg. Freq.
**1**	−1850.859879	−1850.817437	−1850.816492	−1850.937464	0
TS1	−1850.809352	−1850.767289	−1850.766345	−1850.885101	1
Int1	−1850.860106	−1850.817504	−1850.816559	−1850.937417	1
TS2	−1850.821439	−1850.778457	−1850.777513	−1850.899713	1
Int2a	−1850.835729	−1850.791004	−1850.790060	−1850.923188	0
TS3a	−1850.822435	−1850.778665	−1850.777720	−1850.908263	1
Int3a	−1850.882607	−1850.839156	−1850.838212	−1850.969738	0
TS4a	−1850.881216	−1850.839064	−1850.838120	−1850.960089	1
**4**	−1850.957736	−1850.916642	−1850.915698	−1851.034131	0
TSInt2a−b	−1850.831624	−1850.788001	−1850.787057	−1850.914884	1
Int2b	−1850.843135	−1850.798551	−1850.797607	−1850.930495	0
TS3b	−1850.826238	−1850.783215	−1850.782270	−1850.905909	1
Int3b	−1850.870344	−1850.827943	−1850.826999	−1850.947145	0
Int4b	−1850.904026	−1850.861705	−1850.860761	−1850.981300	0
TS5b	−1850.872247	−1850.830721	−1850.829777	−1850.948295	1
Int5b	−1850.913645	−1850.871446	−1850.870502	−1850.990898	0
TS6b	−1850.887519	−1850.846413	−1850.845469	−1850.963256	1
**2**	−1850.936102	−1850.895135	−1850.894191	−1851.011755	0
**3**	−1850.933965	−1850.892861	−1850.891917	−1851.010138	0
TS3b_p	−1850.824889	−1850.781825	−1850.780881	−1850.90458	1
TS2_claisen	−1850.807483	−1850.765354	−1850.764409	−1850.882851	1
TS2_sigma	−1850.761009	−1850.720206	−1850.719262	−1850.834688	1

Table S2. B3LYP/6-31+G* Relative Gibbs Free Energies, direct and inverse Gibbs Free Energies of activation, ⟨S^2⟩ values, and Singlet-Triplet Separations for Structures, Intermediates, and Transition States of the Investigated Rearrangement[a]

Entry	Struct.	ΔG	$\Delta G^{\#}$	$\Delta G^{\#}_{inv}$	$\langle S^2 \rangle$	ΔG_{ST}^{b}

1	**1**	0.00			0.00	
2	TS1	32.86	32.86	32.83	0.00	
3	Int1	0.03			0.00	
4	TS2	23.69	23.66	14.73	0.03	13.19
5	Int2a	8.96			2.00	−0.57
6	TS3a	18.32	9.36	38.57	2.00	−0.65
7	Int3a	−20.25			2.00	−0.61
8	TS4a	−14.20	6.05	46.46	0.34	3.72
9	**4**	−60.66			0.00	
10	TSInt2a-b	14.17	5.21	9.80	2.00	–24.41
11	Int2b	4.37			2.00	−23.53
12	TS3b	19.80	15.43	25.87	0.12	7.03
13	Int3b	−6.07			0.00	
14	TS4b	—	—	—	—	—
15	Int4b	−27.51			0.00	
16	TS5b	−6.80	20.71	26.73	0.00	
17	Int5b	−33.53			0.00	
18	TS6b	−16.18	17.35	26.44	0.00	
19	**2**	−46.62			0.00	
20	**3**	−45.60			0.00	

[a]All energy values are in kcal/mol. [b]ΔG_{ST} = energy of triplet minus energy of spin-contaminated singlet.

Cartesian coordinates of all investigated structures

1

Center Number	Atomic Number	Forces (Hartrees/Bohr) X	Y	Z
1	8	0.000120067	-0.000098317	-0.000014235
2	8	-0.000140950	-0.000044767	-0.000001279
3	6	0.000009430	-0.000004174	-0.000029337
4	8	-0.000093828	0.000093715	-0.000079201
5	6	0.000013332	0.000040353	-0.000028827
6	6	0.000001590	-0.000019396	-0.000043721
7	6	-0.000003633	-0.000057900	-0.000074222
8	6	-0.000077565	-0.000065485	0.000018599
9	6	0.000006255	0.000032234	-0.000003395
10	6	-0.000008409	0.000013188	0.000052930
11	6	0.000002258	0.000041949	-0.000047674
12	6	-0.000014791	-0.000081692	-0.000048327
13	6	0.000001237	0.000060186	-0.000013530
14	6	0.000021488	-0.000070121	-0.000011152
15	6	0.000010067	-0.000026077	-0.000034610
16	6	-0.000017426	0.000027812	0.000000651
17	6	0.000013667	-0.000004870	0.000054488
18	6	0.000089446	0.000018163	-0.000004413
19	6	0.000005341	-0.000033563	-0.000037434
20	8	0.000130684	0.000006885	0.000041010
21	6	0.000066089	0.000104986	-0.000007459
22	6	-0.000080146	-0.000073749	0.000007010
23	6	-0.000023314	0.000093669	-0.000015923
24	6	0.000052641	-0.000032218	0.000103195
25	6	-0.000024759	0.000027001	-0.000003276
26	6	0.000002257	-0.000066493	0.000084607
27	6	-0.000019599	0.000044095	0.000021300
28	6	-0.000067811	0.000023663	-0.000033663
29	6	-0.000055760	-0.000038534	-0.000034337
30	6	-0.000163433	-0.000081794	-0.000050185
31	6	0.000083350	-0.000018736	-0.000000905
32	6	0.000124102	0.000041746	0.000100038
33	6	0.000056222	-0.000008410	0.000003987
34	6	0.000024429	0.000010873	-0.000041866
35	6	-0.000047597	0.000003905	-0.000038725
36	6	-0.000012928	-0.000000030	-0.000024832
37	6	0.000029380	-0.000022985	0.000019758
38	6	-0.000018135	0.000009167	0.000015839
39	1	-0.000024189	-0.000003190	0.000003437
40	1	-0.000006571	0.000010322	0.000021991
41	1	-0.000002576	0.000003033	0.000005511
42	1	-0.000004559	0.000010473	0.000015407
43	1	-0.000008717	0.000002705	0.000008838
44	1	-0.000012231	0.000017370	-0.000008819
45	1	-0.000001266	0.000009998	-0.000003609
46	1	0.000023924	-0.000011936	0.000040571
47	1	-0.000025517	-0.000010831	-0.000001986

48	1	0.000003685	-0.000023319	0.000009283
49	1	0.000000897	0.000012118	-0.000033793
50	1	0.000071966	0.000079859	0.000032304
51	1	0.000011155	-0.000021044	-0.000007702
52	1	0.000023848	-0.000016406	0.000008148
53	1	0.000022965	0.000012182	0.000006238
54	1	0.000032793	0.000020538	-0.000024808
55	1	-0.000071676	0.000080952	-0.000034331
56	1	0.000001646	0.000004521	0.000009687
57	1	-0.000036987	0.000000362	0.000017099
58	1	-0.000019678	-0.000010206	-0.000019482
59	1	-0.000000645	0.000003375	0.000045593
60	1	-0.000053047	0.000025278	0.000032470
61	1	-0.000003542	-0.000004745	0.000010626
62	1	-0.000028362	0.000017222	-0.000023780
63	1	-0.000018351	-0.000062707	-0.000021028
64	1	0.000008771	-0.000044638	-0.000008234
65	1	0.000007527	-0.000004425	-0.000001061
66	1	0.000001027	0.000014161	0.000012613
67	1	-0.000001318	-0.000018776	-0.000010218
68	6	0.000042495	0.000008259	-0.000040386
69	1	-0.000004617	0.000001809	0.000015260
70	1	0.000001805	0.000008686	0.000001152
71	6	0.000046621	0.000012168	0.000024470
72	1	0.000044397	0.000023415	0.000035705
73	1	0.000018262	-0.000006671	0.000033152
74	6	-0.000030277	0.000025793	0.000007892
75	1	0.000028635	0.000000730	0.000005154
76	1	-0.000007165	0.000001163	0.000054911
77	6	-0.000043720	-0.000002145	-0.000041863
78	1	0.000024043	-0.000000165	0.000014190
79	1	0.000001552	-0.000001786	-0.000001874
80	1	0.000005538	-0.000000709	-0.000003465
81	6	0.000024214	0.000029037	0.000000901
82	1	0.000001689	-0.000008231	-0.000001309
83	1	0.000014744	0.000000044	-0.000000919
84	1	-0.000020255	-0.000017472	-0.000012384
85	6	0.000010208	-0.000012477	0.000003366
86	1	-0.000007207	-0.000000768	0.000009101
87	1	-0.000029197	-0.000001109	0.000016472
88	1	-0.000005993	0.000003908	-0.000001376

TS1

Center Number	Atomic Number	Forces (Hartrees/Bohr) X	Y	Z
1	8	-0.000004410	0.000004575	-0.000002327
2	8	-0.000006809	0.000002804	0.000003281
3	6	0.000007184	-0.000009460	-0.000009616
4	8	-0.000001472	-0.000027233	0.000015881
5	6	-0.000000315	0.000000256	0.000000008
6	6	0.000003409	0.000000944	-0.000000681

7	6	-0.000002508	-0.000001567	0.000001611
8	6	-0.000005170	-0.000001159	0.000002522
9	6	0.000002053	0.000003512	0.000004645
10	6	-0.000000750	0.000001143	0.000001835
11	6	0.000000508	0.000000000	-0.000000428
12	6	0.000004312	-0.000004089	0.000015344
13	6	-0.000000739	0.000000271	0.000000095
14	6	-0.000001189	0.000001704	-0.000000243
15	6	0.000003565	-0.000001838	-0.000000344
16	6	0.000003231	0.000000727	-0.000005206
17	6	-0.000001825	-0.000001209	0.000000653
18	6	0.000001393	-0.000000185	-0.000000396
19	6	-0.000003345	-0.000001078	0.000000009
20	8	-0.000012721	0.000010950	-0.000004050
21	6	-0.000004344	0.000015326	-0.000015105
22	6	0.000003441	-0.000001340	0.000001733
23	6	0.000001672	0.000002727	-0.000002513
24	6	-0.000003283	0.000000702	0.000000873
25	6	-0.000001709	-0.000001303	-0.000000563
26	6	-0.000004380	0.000000717	-0.000000222
27	6	-0.000001514	-0.000000639	-0.000000954
28	6	-0.000000282	-0.000000626	0.000000427
29	6	-0.000000827	-0.000000090	-0.000002134
30	6	0.000004125	-0.000003058	0.000000522
31	6	-0.000002424	0.000000128	-0.000000365
32	6	0.000001825	-0.000000269	0.000000731
33	6	-0.000000325	-0.000004654	0.000018374
34	6	-0.000003729	-0.000003817	-0.000000991
35	6	0.000003817	-0.000001642	0.000000272
36	6	0.000011703	-0.000002563	-0.000000586
37	6	-0.000013166	-0.000006380	0.000006716
38	6	0.000003717	0.000009096	-0.000012043
39	1	-0.000000695	0.000000145	-0.000000360
40	1	-0.000000870	0.000000126	-0.000000314
41	1	-0.000000641	0.000000461	-0.000000656
42	1	-0.000000671	0.000000046	-0.000000120
43	1	-0.000001182	-0.000002298	-0.000002323
44	1	-0.000000008	0.000000044	0.000000039
45	1	-0.000000038	-0.000000292	0.000000191
46	1	0.000001967	-0.000000454	0.000004336
47	1	-0.000004383	0.000002122	0.000001671
48	1	0.000000180	-0.000000164	-0.000000143
49	1	-0.000007066	0.000000021	-0.000002673
50	1	0.000004807	0.000002694	0.000003597
51	1	-0.000001919	0.000001715	-0.000006932
52	1	-0.000000394	0.000000263	-0.000000314
53	1	-0.000000734	-0.000000326	0.000000199
54	1	-0.000000071	-0.000000268	-0.000001041
55	1	-0.000002126	0.000000525	0.000001695
56	1	-0.000000066	-0.000000951	0.000000189
57	1	0.000008151	0.000000538	-0.000001230
58	1	0.000002441	0.000000547	-0.000006239
59	6	0.000004318	0.000009950	-0.000005989
60	6	0.000001295	-0.000001412	0.000000326

61	1	0.000001900	0.000000942	0.000001661
62	1	-0.000001017	0.000000873	-0.000000456
63	6	-0.000000339	0.000000464	-0.000000098
64	1	-0.000003396	-0.000001577	-0.000001655
65	1	0.000002247	-0.000000574	-0.000001507
66	1	0.000001104	0.000000633	0.000000718
67	1	0.000000608	0.000000122	-0.000001591
68	1	0.000000044	-0.000000600	0.000000935
69	1	-0.000000332	-0.000000312	0.000000347
70	1	0.000001429	-0.000000809	-0.000001348
71	1	0.000001923	0.000000936	0.000002274
72	1	-0.000001477	0.000001281	0.000000091
73	6	0.000000473	-0.000001426	-0.000000127
74	1	0.000000639	-0.000000332	0.000001980
75	1	0.000000043	0.000000473	-0.000000122
76	1	0.000000503	-0.000001811	0.000000259
77	1	-0.000001572	0.000001294	-0.000003218
78	1	0.000001479	-0.000000473	0.000002518
79	6	0.000000318	0.000003021	-0.000000117
80	1	0.000001630	0.000003189	-0.000002862
81	1	-0.000000944	0.000001645	0.000002537
82	1	0.000000650	-0.000002874	-0.000001537
83	1	0.000006272	-0.000000123	0.000001290
84	1	0.000005612	0.000002043	0.000000237
85	6	-0.000001091	-0.000000580	-0.000000907
86	1	0.000001504	-0.000000707	-0.000000395
87	1	0.000001195	0.000001180	0.000000604
88	1	-0.000000419	-0.000000310	-0.000000187

Int1

Center Number	Atomic Number	Forces (Hartrees/Bohr) X	Y	Z
1	8	0.000002327	0.000005701	-0.000003186
2	8	0.000003382	0.000002946	-0.000001651
3	6	-0.000015871	0.000018689	-0.000018645
4	8	-0.000003987	-0.000003665	0.000000959
5	6	-0.000002628	-0.000000253	-0.000000909
6	6	-0.000000206	-0.000000433	-0.000000244
7	6	-0.000002603	0.000002908	0.000003707
8	6	0.000008516	-0.000006029	0.000006170
9	6	-0.000001738	-0.000000686	0.000000619
10	6	-0.000000487	0.000000405	-0.000002110
11	6	0.000001454	0.000000620	-0.000000117
12	6	-0.000001190	-0.000002770	0.000006735
13	6	0.000000720	0.000001538	0.000000081
14	6	-0.000001001	-0.000001606	0.000004156
15	6	0.000004310	-0.000001900	-0.000000009
16	6	0.000002755	0.000000095	-0.000002575
17	6	-0.000000561	-0.000001331	-0.000000545
18	6	-0.000000779	-0.000002398	-0.000002323
19	6	-0.000001046	0.000002535	-0.000000739

20	8	0.000008308	-0.000007356	0.000006288
21	6	-0.000002079	-0.000002606	0.000001374
22	6	-0.000003845	0.000003238	-0.000002380
23	6	-0.000003179	-0.000001085	0.000001473
24	6	0.000003004	0.000001603	-0.000000741
25	6	0.000000150	-0.000000247	-0.000002104
26	6	0.000002412	0.000003395	0.000000276
27	6	-0.000000209	-0.000002609	0.000000665
28	6	0.000000438	-0.000000704	0.000000906
29	6	-0.000000985	-0.000002660	-0.000001371
30	6	-0.000000745	-0.000009173	0.000001781
31	6	0.000004221	0.000004638	0.000001646
32	6	-0.000002584	-0.000000350	-0.000001742
33	6	0.000001676	0.000000373	-0.000000381
34	6	0.000001820	0.000000643	0.000000180
35	6	0.000002212	-0.000001111	-0.000001036
36	6	-0.000003648	-0.000008337	-0.000002828
37	6	0.000006745	0.000011642	0.000006423
38	6	-0.000002224	-0.000000979	-0.000003897
39	1	-0.000000676	0.000000482	-0.000000424
40	1	-0.000000651	0.000001762	0.000000498
41	1	0.000000377	0.000001624	-0.000000334
42	1	0.000000610	0.000000080	0.000000946
43	1	-0.000000457	-0.000000158	-0.000000711
44	1	-0.000000554	-0.000001025	0.000000014
45	1	0.000000612	-0.000000660	-0.000000897
46	1	-0.000000778	0.000000407	-0.000000943
47	1	0.000000561	0.000000346	0.000000930
48	1	-0.000000193	0.000001072	0.000001050
49	1	-0.000000385	0.000001090	-0.000000293
50	1	0.000001283	0.000000635	-0.000000014
51	1	-0.000000787	-0.000001342	-0.000000025
52	1	-0.000000866	0.000000035	-0.000000305
53	1	-0.000000357	-0.000001092	0.000000785
54	1	-0.000000224	-0.000000670	-0.000000816
55	1	0.000000149	-0.000001658	0.000001617
56	1	0.000000435	-0.000000638	0.000000092
57	1	-0.000000462	0.000001003	0.000000964
58	1	-0.000001249	-0.000001196	-0.000000696
59	6	0.000002029	0.000000676	-0.000000060
60	6	0.000000174	-0.000000408	0.000000484
61	1	-0.000000242	0.000001530	0.000000548
62	1	0.000000164	0.000001158	0.000000528
63	6	-0.000000159	-0.000000677	0.000000025
64	1	0.000000236	0.000000256	-0.000000205
65	1	0.000000247	-0.000000139	-0.000000561
66	1	0.000001495	0.000000548	-0.000000680
67	1	-0.000000296	0.000001500	-0.000000233
68	1	0.000000547	0.000000714	0.000001993
69	1	-0.000000225	0.000000843	-0.000000067
70	1	0.000000703	-0.000000462	0.000000131
71	1	0.000000341	-0.000000476	-0.000000453
72	1	0.000000106	0.000000069	-0.000000022
73	6	-0.000000083	-0.000000089	0.000000125

74	1	0.000000699	-0.000000531	-0.000000252
75	1	0.000000475	-0.000000392	-0.000000511
76	1	0.000000270	-0.000000751	-0.000000391
77	1	0.000000208	-0.000000201	0.000000510
78	1	-0.000000317	0.000000119	0.000000571
79	6	-0.000000487	0.000000174	0.000000456
80	1	-0.000000512	0.000000087	0.000000305
81	1	-0.000000274	-0.000000014	0.000000485
82	1	-0.000000291	-0.000000130	0.000000047
83	1	-0.000000612	-0.000001536	0.000000927
84	1	-0.000002011	-0.000001583	0.000000319
85	6	-0.000001298	-0.000001876	-0.000000053
86	1	-0.000000186	-0.000000417	-0.000000437
87	1	-0.000000136	-0.000000438	0.000001097
88	1	0.000000192	-0.000000327	0.000000032

TS2

Center Number	Atomic Number	Forces (Hartrees/Bohr) X	Y	Z
1	8	-0.000000398	0.000000096	0.000000366
2	8	0.000000724	-0.000000013	0.000000153
3	6	0.000000397	0.000000127	-0.000001036
4	8	-0.000000534	-0.000000415	-0.000000168
5	6	0.000000124	-0.000000085	0.000000011
6	6	0.000000329	0.000000181	0.000000047
7	6	0.000000055	0.000000017	-0.000000055
8	6	0.000000160	-0.000000502	0.000000760
9	6	0.000000355	-0.000000348	-0.000000029
10	6	-0.000000741	-0.000000554	0.000000234
11	6	0.000000229	0.000001237	0.000001672
12	6	0.000000446	-0.000002214	0.000000197
13	6	0.000000211	0.000000039	-0.000000504
14	6	-0.000000049	0.000000232	-0.000000113
15	6	0.000000051	0.000000181	0.000000270
16	6	0.000000314	0.000000232	-0.000000662
17	6	0.000000186	0.000000005	-0.000000361
18	6	-0.000000673	-0.000000156	0.000000312
19	6	-0.000000080	-0.000000609	-0.000000247
20	8	0.000001270	-0.000001439	-0.000000182
21	6	0.000000931	0.000000130	-0.000001079
22	6	-0.000000108	0.000000335	-0.000000139
23	6	-0.000000047	-0.000000024	-0.000000731
24	6	0.000000571	-0.000000099	-0.000000041
25	6	-0.000000360	-0.000000189	0.000000158
26	6	0.000000316	-0.000000150	-0.000000142
27	6	0.000000005	-0.000000503	-0.000000074
28	6	0.000000336	-0.000000252	-0.000000079
29	6	-0.000000249	0.000001022	-0.000000465
30	6	-0.000000113	0.000000032	0.000000047
31	6	0.000000064	-0.000000398	-0.000000088
32	6	0.000001034	-0.000000039	0.000000266

33	6	-0.000000494	0.000000426	0.000000269
34	6	0.000000014	-0.000000345	-0.000000290
35	6	-0.000000364	0.000000598	-0.000000081
36	6	-0.000000323	-0.000000202	0.000000520
37	6	0.000000189	0.000000036	0.000000541
38	6	-0.000000618	0.000000687	0.000000039
39	1	0.000000273	-0.000000237	-0.000000046
40	1	0.000000123	-0.000000171	0.000000185
41	1	0.000000114	-0.000000235	0.000000438
42	1	0.000000108	-0.000000447	0.000000511
43	1	0.000000062	-0.000000444	0.000000626
44	1	0.000000255	0.000000012	-0.000000681
45	1	0.000000123	0.000000070	-0.000000497
46	1	0.000000100	0.000000131	-0.000000696
47	1	-0.000000525	0.000000321	-0.000000052
48	1	0.000000269	-0.000000032	-0.000000541
49	1	-0.000000093	0.000000137	-0.000000269
50	1	0.000000038	0.000000329	0.000000261
51	1	-0.000000171	-0.000000112	0.000000382
52	1	0.000000205	-0.000000091	-0.000000227
53	1	0.000000336	-0.000000375	-0.000000105
54	1	0.000000040	-0.000001088	0.000000194
55	1	0.000000117	-0.000000499	0.000000590
56	1	0.000000173	-0.000000447	0.000000160
57	1	-0.000000093	0.000000271	-0.000000623
58	1	0.000000304	0.000000322	0.000000021
59	6	-0.000001678	0.000000422	0.000000285
60	6	0.000000081	0.000000141	-0.000000109
61	1	0.000000086	-0.000000212	-0.000000106
62	1	0.000000289	0.000000068	-0.000000100
63	6	-0.000000451	0.000000273	0.000000089
64	1	-0.000000177	-0.000000071	-0.000000155
65	1	-0.000000036	0.000000101	0.000000282
66	1	-0.000000113	-0.000000159	0.000000639
67	1	-0.000000048	-0.000000218	0.000000539
68	1	-0.000000146	-0.000000066	0.000000289
69	1	0.000000355	-0.000000114	-0.000000332
70	1	0.000000409	-0.000000109	-0.000000418
71	1	-0.000000301	0.000000217	0.000000173
72	1	-0.000000228	0.000000291	-0.000000026
73	6	-0.000000397	0.000000437	0.000000181
74	1	-0.000000327	0.000000387	0.000000066
75	1	-0.000000385	0.000000329	0.000000196
76	1	-0.000000404	0.000000433	0.000000131
77	1	-0.000000083	-0.000000054	-0.000000166
78	1	-0.000000006	0.000000104	0.000000011
79	6	-0.000000112	0.000000108	-0.000000049
80	1	-0.000000005	0.000000016	-0.000000085
81	1	0.000000038	0.000000053	-0.000000196
82	1	-0.000000047	0.000000116	-0.000000105
83	1	-0.000000398	0.000001247	0.000000097
84	1	0.000000224	0.000000475	0.000000152
85	6	-0.000000309	0.000000147	-0.000000119
86	1	-0.000000352	0.000000387	-0.000000078

87	1	-0.000000167	0.000000346	-0.000000115
88	1	-0.000000233	0.000000413	0.000000107

Int2a

Center Number	Atomic Number	Forces (Hartrees/Bohr) X	Y	Z
1	8	0.000003321	-0.000000786	-0.000000401
2	8	-0.000005831	-0.000000231	-0.000000999
3	6	0.000002783	-0.000000968	-0.000003435
4	8	-0.000005238	-0.000002041	-0.000002184
5	6	0.000002033	0.000002691	-0.000000047
6	6	-0.000001299	-0.000000757	-0.000002755
7	6	-0.000000492	-0.000002679	0.000000948
8	6	-0.000004570	0.000000497	0.000003861
9	6	0.000000685	0.000003542	-0.000001913
10	6	0.000002474	-0.000003085	0.000000153
11	6	-0.000002314	0.000000140	0.000002682
12	6	0.000002456	0.000002260	-0.000000407
13	6	-0.000000265	0.000000677	-0.000001011
14	6	-0.000000706	0.000000462	-0.000000835
15	6	-0.000000253	-0.000002578	0.000000941
16	6	-0.000001898	-0.000000379	-0.000001243
17	6	0.000000375	0.000000279	0.000001916
18	6	0.000003632	0.000002303	0.000000262
19	6	0.000000131	-0.000000110	-0.000001039
20	8	-0.000000492	-0.000001432	0.000001832
21	6	0.000004537	0.000004253	-0.000000041
22	6	-0.000000004	-0.000001631	-0.000002740
23	6	-0.000001219	0.000003391	-0.000002587
24	6	-0.000000341	0.000001454	0.000004419
25	6	0.000003389	0.000000942	0.000000734
26	6	-0.000000651	-0.000000857	0.000002075
27	6	-0.000001411	-0.000000118	-0.000001651
28	6	-0.000000155	0.000001164	-0.000000308
29	6	0.000001635	-0.000002596	-0.000000773
30	6	-0.000001968	-0.000001022	-0.000002092
31	6	0.000000812	-0.000000568	-0.000001335
32	6	-0.000000609	0.000001713	0.000001566
33	6	0.000002922	-0.000000525	0.000002643
34	6	-0.000002181	0.000000313	-0.000000369
35	6	0.000005791	-0.000002572	0.000000171
36	6	0.000003680	0.000002393	-0.000000153
37	6	-0.000000249	-0.000001379	0.000001511
38	6	-0.000002182	0.000000387	-0.000000789
39	1	-0.000000047	-0.000000039	-0.000000021
40	1	0.000000204	0.000000088	0.000000522
41	1	0.000000101	-0.000000483	0.000000649
42	1	-0.000000010	0.000000462	0.000000625
43	1	0.000000347	0.000000088	0.000000398
44	1	0.000000366	-0.000000035	-0.000000415
45	1	-0.000000003	-0.000000243	0.000000117

46	1	0.000000101	-0.000000521	0.000000679
47	1	0.000001746	-0.000000297	-0.000001075
48	1	-0.000000112	0.000000489	-0.000000736
49	1	-0.000000261	-0.000001519	0.000001653
50	1	-0.000000355	-0.000000336	-0.000000326
51	1	-0.000000506	0.000000646	-0.000000557
52	1	0.000000499	0.000000666	-0.000000370
53	1	-0.000000148	0.000000352	0.000000058
54	1	-0.000000262	0.000000842	0.000000407
55	1	-0.000000304	0.000001152	0.000000103
56	1	0.000000108	0.000000571	0.000000469
57	1	-0.000000694	-0.000000909	-0.000001177
58	1	-0.000001653	0.000001803	0.000000734
59	6	0.000000202	-0.000000199	-0.000001016
60	6	-0.000002095	-0.000000151	0.000001111
61	1	0.000000532	0.000000078	0.000000012
62	1	0.000000499	-0.000000110	-0.000000113
63	6	0.000000813	-0.000000731	0.000000237
64	1	-0.000001164	0.000000568	-0.000000231
65	1	-0.000001030	-0.000000037	0.000000289
66	1	-0.000000613	-0.000000049	0.000000555
67	1	-0.000001653	-0.000000654	0.000000501
68	1	0.000000379	-0.000000022	0.000000157
69	1	-0.000000275	0.000000723	-0.000000056
70	1	-0.000000105	-0.000000409	-0.000000275
71	1	-0.000000185	-0.000000419	-0.000000243
72	1	-0.000000222	-0.000000177	-0.000000482
73	6	-0.000001582	0.000000471	-0.000000039
74	1	0.000000181	-0.000000516	-0.000000153
75	1	0.000000248	-0.000000497	-0.000000026
76	1	0.000000213	-0.000000611	-0.000000005
77	1	-0.000000091	-0.000000175	-0.000000504
78	1	0.000000201	-0.000000258	-0.000000275
79	6	0.000002056	0.000000233	-0.000000369
80	1	-0.000000542	-0.000000180	-0.000000073
81	1	-0.000000285	-0.000000152	0.000000037
82	1	-0.000000415	0.000000005	0.000000011
83	1	-0.000000378	-0.000000530	0.000000195
84	1	0.000000191	-0.000001193	0.000001377
85	6	-0.000000714	0.000000563	0.000001176
86	1	-0.000000009	0.000000330	-0.000000152
87	1	-0.000000267	-0.000000963	0.000000127
88	1	0.000000667	-0.000000267	-0.000000121

TS3a

Center Number	Atomic Number	Forces (Hartrees/Bohr) X	Y	Z
1	8	-0.000000105	-0.000000127	0.000000055
2	8	0.000000104	-0.000000058	0.000000060
3	6	-0.000002546	-0.000000510	0.000002309
4	8	-0.000000106	-0.000000081	0.000000121

5	6	-0.000000026	0.000000326	0.000000020
6	6	0.000000051	0.000000277	0.000000156
7	6	-0.000000001	0.000000136	0.000000100
8	6	0.000001222	0.000000309	-0.000000466
9	6	-0.000001090	-0.000000667	0.000001120
10	6	-0.000001066	0.000002078	0.000000597
11	6	0.000001890	-0.000000195	-0.000000439
12	6	-0.000002038	0.000001312	0.000000707
13	6	0.000000182	0.000000142	-0.000000405
14	6	0.000000157	0.000000193	-0.000000315
15	6	0.000000252	0.000000025	-0.000000249
16	6	0.000000129	0.000000085	-0.000000179
17	6	0.000000085	0.000000060	-0.000000380
18	6	0.000000186	0.000000288	-0.000000189
19	6	0.000000031	0.000000156	-0.000000119
20	8	0.000003496	-0.000003792	-0.000001407
21	6	0.000000077	-0.000000101	-0.000000187
22	6	0.000000120	-0.000000010	-0.000000193
23	6	0.000000072	-0.000000051	-0.000000152
24	6	-0.000000132	-0.000000012	-0.000000084
25	6	-0.000000142	0.000000146	0.000000212
26	6	0.000000141	0.000000180	-0.000000132
27	6	-0.000000071	0.000000144	-0.000000168
28	6	0.000000168	0.000000172	0.000000173
29	6	-0.000000053	-0.000000045	0.000000239
30	6	0.000000014	-0.000000092	0.000000015
31	6	-0.000000067	0.000000102	0.000000140
32	6	-0.000000114	0.000000009	0.000000084
33	6	0.000000020	-0.000000135	-0.000000197
34	6	-0.000000047	-0.000000208	-0.000000020
35	6	0.000000058	0.000000069	-0.000000137
36	6	0.000002377	-0.000000004	-0.000000806
37	6	-0.000003392	0.000005929	0.000002815
38	6	0.000000450	-0.000002867	-0.000001021
39	1	0.000000099	0.000000406	0.000000025
40	1	0.000000063	0.000000362	0.000000186
41	1	-0.000000014	0.000000272	0.000000324
42	1	-0.000000163	0.000000344	0.000000386
43	1	-0.000000135	0.000000204	0.000000513
44	1	0.000000218	0.000000171	-0.000000431
45	1	0.000000189	0.000000177	-0.000000382
46	1	0.000000105	0.000000121	-0.000000272
47	1	0.000000140	0.000000020	-0.000000344
48	1	0.000000140	0.000000068	-0.000000337
49	1	0.000000038	-0.000000071	-0.000000190
50	1	0.000000003	0.000000016	0.000000068
51	1	-0.000000008	0.000000165	0.000000081
52	1	0.000000081	0.000000261	-0.000000136
53	1	0.000000028	0.000000268	0.000000077
54	1	-0.000000135	-0.000000011	0.000000288
55	1	-0.000000149	-0.000000179	0.000000218
56	1	-0.000000041	0.000000161	0.000000232
57	1	-0.000000017	-0.000000197	-0.000000197
58	1	0.000000126	-0.000000150	-0.000000189

59	6	0.000000027	-0.000000183	0.000000031
60	6	-0.000000129	-0.000000352	0.000000035
61	1	-0.000000041	-0.000000193	0.000000031
62	1	-0.000000035	-0.000000201	-0.000000092
63	6	0.000000052	-0.000000061	-0.000000153
64	1	0.000000127	0.000000100	-0.000000185
65	1	0.000000076	0.000000124	-0.000000099
66	1	-0.000000235	-0.000000312	-0.000000124
67	1	-0.000000231	-0.000000164	0.000000332
68	1	-0.000000114	0.000000063	0.000000697
69	1	0.000000117	0.000000221	-0.000000120
70	1	0.000000096	-0.000000066	-0.000000188
71	1	0.000000016	-0.000000092	-0.000000105
72	1	0.000000019	-0.000000105	-0.000000249
73	6	0.000000071	-0.000000066	-0.000000215
74	1	0.000000108	-0.000000021	-0.000000296
75	1	0.000000057	-0.000000010	-0.000000168
76	1	0.000000057	-0.000000137	-0.000000232
77	1	-0.000000071	-0.000000368	-0.000000060
78	1	-0.000000118	-0.000000363	0.000000071
79	6	-0.000000082	-0.000000418	-0.000000065
80	1	-0.000000094	-0.000000402	0.000000004
81	1	-0.000000048	-0.000000402	-0.000000127
82	1	-0.000000112	-0.000000505	-0.000000046
83	1	-0.000000015	-0.000000218	-0.000000132
84	1	-0.000000046	-0.000000176	0.000000037
85	6	-0.000000078	-0.000000293	-0.000000022
86	1	-0.000000050	-0.000000362	-0.000000086
87	1	-0.000000062	-0.000000300	-0.000000105
88	1	-0.000000111	-0.000000325	0.000000035

Int3a

Center Number	Atomic Number	Forces (Hartrees/Bohr) X	Y	Z
1	8	0.000000368	-0.000000843	0.000000812
2	8	-0.000004333	-0.000001960	0.000000004
3	6	0.000000342	-0.000001480	0.000000348
4	8	-0.000000394	0.000000977	-0.000001122
5	6	-0.000000427	-0.000000443	-0.000000456
6	6	-0.000000115	0.000000469	0.000000839
7	6	0.000000552	-0.000002132	-0.000003243
8	6	-0.000000743	0.000001146	0.000000335
9	6	0.000000481	-0.000000496	-0.000000357
10	6	0.000000154	-0.000001171	0.000000074
11	6	-0.000000440	0.000000189	0.000000179
12	6	0.000001160	0.000000036	0.000000674
13	6	-0.000000649	-0.000000305	0.000000119
14	6	0.000000428	0.000000417	-0.000000489
15	6	-0.000002220	0.000001774	0.000001412
16	6	0.000001133	-0.000001781	0.000000378
17	6	-0.000000735	-0.000000094	0.000000243

18	6	0.000002117	0.000001357	-0.000000011
19	6	-0.000000074	0.000000358	-0.000000810
20	8	-0.000000194	-0.000000098	0.000000039
21	6	0.000001042	-0.000001360	-0.000000135
22	6	0.000002128	-0.000000289	0.000000242
23	6	-0.000002221	0.000000481	0.000000160
24	6	-0.000000348	0.000002327	0.000002675
25	6	0.000001253	-0.000000027	0.000001420
26	6	-0.000000293	-0.000000629	-0.000001351
27	6	0.000000810	0.000000451	0.000005300
28	6	0.000000195	-0.000000992	-0.000000467
29	6	-0.000000099	-0.000000057	-0.000000002
30	6	0.000000742	-0.000000653	-0.000001523
31	6	-0.000000302	0.000000370	0.000000394
32	6	0.000000657	0.000001008	-0.000000681
33	6	-0.000000143	-0.000000437	0.000000890
34	6	-0.000000337	0.000000978	-0.000000010
35	6	0.000001689	0.000001301	0.000000297
36	6	0.000000546	-0.000000305	-0.000000362
37	6	-0.000000007	0.000000101	0.000000565
38	6	0.000000311	-0.000000234	-0.000000501
39	1	0.000000165	-0.000000116	0.000000079
40	1	0.000000174	-0.000000513	0.000000095
41	1	0.000000031	-0.000000376	0.000000286
42	1	0.000000123	-0.000000408	0.000000124
43	1	0.000000307	-0.000000434	0.000000171
44	1	0.000000114	0.000000147	-0.000000155
45	1	-0.000000436	-0.000000325	0.000000186
46	1	-0.000000043	0.000000279	-0.000001096
47	1	-0.000000120	0.000000021	-0.000001082
48	1	0.000000094	0.000000412	-0.000000244
49	1	-0.000000136	-0.000000224	-0.000000137
50	1	-0.000000706	0.000000132	-0.000000356
51	1	-0.000000298	0.000000498	-0.000000041
52	1	-0.000000305	0.000000167	0.000000226
53	1	0.000000205	0.000000060	-0.000000011
54	1	0.000000471	0.000000191	-0.000000154
55	1	0.000000252	0.000000358	-0.000000310
56	1	0.000000485	0.000000055	-0.000000107
57	1	-0.000000156	0.000000125	-0.000000292
58	1	-0.000000145	0.000000333	0.000000021
59	6	0.000000040	0.000001149	-0.000000420
60	6	-0.000000037	0.000000444	-0.000000339
61	1	0.000000032	0.000000122	-0.000000277
62	1	0.000000121	0.000000108	-0.000000307
63	6	-0.000000281	-0.000000145	0.000000071
64	1	-0.000000767	-0.000000126	-0.000000661
65	1	-0.000000487	-0.000001162	0.000000786
66	1	0.000000389	0.000000043	0.000000096
67	1	-0.000000128	-0.000000173	0.000000072
68	1	0.000000211	-0.000000241	-0.000000232
69	1	0.000000066	-0.000000159	-0.000000216
70	1	0.000001149	0.000000426	-0.000001444
71	1	-0.000000201	-0.000000116	0.000000147

72	1	-0.000000169	-0.000000090	0.000000186
73	6	-0.000000384	-0.000000216	0.000000100
74	1	-0.000000362	-0.000000148	0.000000188
75	1	-0.000000273	-0.000000250	0.000000215
76	1	-0.000000333	-0.000000103	0.000000194
77	1	-0.000000086	0.000000314	-0.000000072
78	1	0.000000115	0.000000365	-0.000000174
79	6	0.000000038	0.000000437	-0.000000265
80	1	0.000000105	0.000000437	-0.000000274
81	1	0.000000010	0.000000477	-0.000000240
82	1	0.000000034	0.000000446	-0.000000239
83	1	-0.000000042	-0.000000310	0.000000032
84	1	-0.000000193	-0.000000298	0.000000171
85	6	-0.000000172	0.000000202	-0.000000358
86	1	-0.000000137	0.000000168	0.000000011
87	1	-0.000000218	-0.000000072	0.000000153
88	1	-0.000000150	0.000000132	0.000000012

TS4a

Center Number	Atomic Number	Forces (Hartrees/Bohr) X	Y	Z
1	8	0.000000086	-0.000000096	0.000000207
2	8	0.000000451	0.000000153	-0.000000109
3	6	-0.000000060	0.000000401	0.000000078
4	8	0.000000029	-0.000000587	-0.000000408
5	6	-0.000000196	0.000000308	-0.000000345
6	6	-0.000000166	0.000000297	-0.000000068
7	6	0.000000069	-0.000000015	-0.000000408
8	6	-0.000000009	0.000000053	-0.000000372
9	6	-0.000000238	0.000000294	-0.000000177
10	6	-0.000000305	0.000000342	-0.000000226
11	6	-0.000000378	0.000000296	-0.000000002
12	6	-0.000000210	0.000000057	-0.000000075
13	6	-0.000000014	0.000000040	-0.000000140
14	6	0.000000112	-0.000000003	-0.000000040
15	6	-0.000000192	0.000000261	-0.000000286
16	6	0.000000376	0.000000017	0.000000133
17	6	0.000000069	-0.000000080	0.000000018
18	6	0.000000084	0.000000256	-0.000000073
19	6	0.000000036	0.000000062	0.000000284
20	8	-0.000000141	0.000000282	0.000000153
21	6	0.000000283	-0.000000002	0.000000072
22	6	-0.000000037	-0.000000112	-0.000000072
23	6	0.000000038	-0.000000259	0.000000396
24	6	-0.000000281	-0.000000127	-0.000000071
25	6	-0.000000012	0.000000183	-0.000000312
26	6	0.000000017	0.000000190	-0.000000102
27	6	0.000000102	-0.000000015	-0.000000505
28	6	-0.000000447	0.000000096	-0.000000031
29	6	0.000001759	0.000000134	0.000001658
30	6	0.000000033	-0.000000040	0.000000326

31	6	-0.000000304	0.000000118	0.000000743
32	6	-0.000000368	-0.000000594	-0.000000271
33	6	0.000000108	-0.000000129	-0.000000057
34	6	-0.000000185	-0.000000189	0.000000368
35	6	0.000000265	0.000000153	-0.000000350
36	6	-0.000002386	-0.000000133	-0.000001037
37	6	0.000001594	0.000000435	-0.000000730
38	6	-0.000000861	-0.000000298	0.000000482
39	1	-0.000000187	0.000000302	-0.000000165
40	1	-0.000000192	0.000000394	-0.000000134
41	1	-0.000000129	0.000000395	-0.000000305
42	1	-0.000000284	0.000000445	-0.000000204
43	1	-0.000000358	0.000000332	-0.000000016
44	1	0.000000064	-0.000000000	-0.000000068
45	1	0.000000212	0.000000117	-0.000000203
46	1	0.000000087	0.000000145	-0.000000429
47	1	0.000000246	0.000000146	-0.000000109
48	1	0.000000006	-0.000000139	0.000000117
49	1	0.000000107	-0.000000074	0.000000254
50	1	-0.000000016	0.000000214	-0.000000077
51	1	0.000000016	0.000000286	-0.000000241
52	1	-0.000000085	0.000000244	-0.000000184
53	1	-0.000000369	0.000000155	0.000000191
54	1	-0.000000681	0.000000122	0.000000243
55	1	-0.000000739	-0.000000027	0.000000174
56	1	-0.000000389	0.000000073	0.000000260
57	1	0.000000112	-0.000000253	0.000000062
58	1	0.000000101	-0.000000069	-0.000000050
59	6	0.000000209	-0.000000182	0.000000087
60	6	-0.000000033	-0.000000367	0.000000479
61	1	-0.000000014	-0.000000322	0.000000399
62	1	-0.000000034	-0.000000361	0.000000463
63	6	0.000000335	0.000000016	-0.000000350
64	1	0.000000165	0.000000160	-0.000000465
65	1	0.000000166	0.000000188	-0.000000390
66	1	-0.000000002	0.000000016	0.000000712
67	1	-0.000000064	-0.000000082	0.000000342
68	1	-0.000000258	0.000000140	0.000000203
69	1	-0.000000250	0.000000094	0.000000153
70	1	-0.000000053	-0.000000120	0.000000204
71	1	0.000000194	0.000000084	-0.000000392
72	1	0.000000394	-0.000000167	-0.000000304
73	6	0.000000403	0.000000076	-0.000000456
74	1	0.000000433	0.000000079	-0.000000482
75	1	0.000000379	0.000000124	-0.000000532
76	1	0.000000470	0.000000003	-0.000000469
77	1	0.000000080	-0.000000364	0.000000350
78	1	0.000000023	-0.000000386	0.000000389
79	6	0.000000031	-0.000000412	0.000000433
80	1	-0.000000060	-0.000000452	0.000000550
81	1	0.000000013	-0.000000493	0.000000493
82	1	0.000000082	-0.000000529	0.000000511
83	1	0.000000115	-0.000000212	0.000000009
84	1	-0.000000078	-0.000000043	0.000000240

85	6	0.000000261	-0.000000232	0.000000060
86	1	0.000000291	-0.000000335	0.000000043
87	1	0.000000314	-0.000000227	-0.000000074
88	1	0.000000243	-0.000000253	0.000000032

4

Center Number	Atomic Number	Forces (Hartrees/Bohr) X	Y	Z
1	8	0.000001009	0.000007864	0.000003084
2	8	-0.000006373	-0.000007612	0.000001086
3	6	0.000008311	0.000005163	0.000004384
4	8	0.000001120	-0.000001078	0.000007804
5	6	-0.000005151	0.000005360	-0.000001192
6	6	-0.000001782	-0.000001406	0.000000526
7	6	0.000004357	0.000007599	-0.000006670
8	6	-0.000002498	-0.000003542	-0.000000072
9	6	0.000000602	0.000002468	-0.000006780
10	6	0.000002402	-0.000001403	0.000003649
11	6	-0.000003660	-0.000001643	-0.000005271
12	6	-0.000001533	0.000000826	-0.000001325
13	6	-0.000001320	0.000001997	-0.000002190
14	6	-0.000000957	0.000001678	0.000011588
15	6	0.000003544	0.000000044	-0.000006536
16	6	-0.000008491	-0.000000954	0.000002392
17	6	-0.000000164	0.000003618	-0.000000323
18	6	0.000002158	0.000003699	-0.000001832
19	6	-0.000003792	-0.000005409	-0.000000563
20	8	-0.000004793	0.000000499	0.000006971
21	6	0.000004431	0.000005908	-0.000008022
22	6	-0.000010153	-0.000013114	-0.000001166
23	6	0.000005312	0.000000743	-0.000002589
24	6	0.000004987	0.000008063	-0.000005089
25	6	-0.000002197	-0.000001650	0.000006546
26	6	0.000011308	-0.000007664	-0.000002630
27	6	-0.000010170	0.000004867	0.000005375
28	6	0.000002922	0.000005347	0.000001381
29	6	0.000002593	0.000001915	0.000005627
30	6	0.000004928	-0.000012604	-0.000002813
31	6	-0.000002524	-0.000000522	-0.000004324
32	6	-0.000000327	0.000000004	0.000001299
33	6	-0.000001162	0.000007921	0.000005084
34	6	-0.000005809	-0.000006132	-0.000000436
35	6	0.000012003	0.000004694	0.000003257
36	6	0.000001223	-0.000002211	0.000004830
37	6	0.000001592	-0.000000940	-0.000008088
38	6	-0.000003327	-0.000002291	-0.000001841
39	1	-0.000000239	0.000001528	0.000000541
40	1	0.000001730	0.000000940	0.000000057
41	1	-0.000000056	0.000000045	0.000000603
42	1	-0.000000248	0.000000656	0.000000662
43	1	0.000000585	0.000000372	0.000000003

44	1	-0.000000033	0.000000772	0.000000450
45	1	0.000000853	0.000000394	0.000000421
46	1	-0.000000115	-0.000001257	-0.000002216
47	1	0.000002411	-0.000002699	0.000003736
48	1	0.000000427	-0.000000188	-0.000001790
49	1	-0.000000171	0.000005285	-0.000003054
50	1	-0.000000358	-0.000003109	-0.000002030
51	1	-0.000001914	-0.000000864	0.000002154
52	1	0.000000158	0.000000643	0.000001105
53	1	-0.000000435	-0.000000930	-0.000000766
54	1	-0.000000488	0.000000150	0.000000850
55	1	0.000002719	-0.000004986	0.000005215
56	1	-0.000000343	-0.000001328	-0.000002864
57	6	0.000005153	0.000007103	-0.000003502
58	6	-0.000000329	0.000000768	-0.000000661
59	1	0.000001349	0.000000499	-0.000001038
60	1	0.000000361	0.000001681	-0.000000204
61	6	-0.000000465	0.000000989	-0.000000426
62	1	-0.000001144	0.000000686	-0.000000846
63	1	-0.000000342	-0.000003781	0.000001681
64	1	-0.000000457	0.000000857	-0.000002980
65	1	0.000000325	-0.000004042	0.000000804
66	1	0.000000586	0.000000982	-0.000000071
67	1	-0.000000924	-0.000003130	0.000001850
68	1	-0.000000657	-0.000000523	0.000001037
69	1	0.000000395	-0.000000184	-0.000000091
70	6	-0.000002049	0.000000475	-0.000001863
71	1	-0.000000528	0.000000221	0.000000496
72	1	0.000000734	-0.000000189	0.000000326
73	1	-0.000001079	0.000000466	0.000000408
74	1	-0.000000272	-0.000000248	-0.000000573
75	1	0.000000033	-0.000000557	0.000000148
76	6	0.000002024	0.000000736	-0.000000862
77	1	-0.000000481	-0.000000458	-0.000000688
78	1	-0.000000220	-0.000000123	-0.000000386
79	1	-0.000000348	-0.000000322	-0.000000376
80	1	-0.000000736	-0.000002476	0.000001173
81	1	0.000000374	-0.000004351	-0.000001429
82	6	-0.000005204	-0.000002069	-0.000001727
83	1	0.000001268	0.000000478	0.000000292
84	1	0.000001038	-0.000001187	0.000001045
85	1	-0.000000523	-0.000000461	0.000001045
86	1	0.000001763	0.000002204	0.000003652
87	1	-0.000005418	0.000000999	-0.000003270
88	1	0.000002678	-0.000000571	-0.000001170

TSInt2a-b

Center Number	Atomic Number	Forces (Hartrees/Bohr) X	Y	Z
1	8	0.000000140	0.000000233	-0.000000273
2	8	-0.000000124	0.000000301	0.000000064

3	6	0.000000303	-0.000000043	0.000000064
4	8	-0.000001192	0.000000505	0.000000510
5	6	0.000000205	-0.000000345	0.000000178
6	6	-0.000000678	0.000000315	0.000000291
7	6	-0.000000101	-0.000000097	-0.000000011
8	6	0.000000040	-0.000000417	-0.000000729
9	6	-0.000000221	-0.000000331	-0.000000230
10	6	0.000000188	-0.000000298	-0.000000184
11	6	0.000000123	-0.000000143	-0.000000305
12	6	0.000000241	-0.000000011	-0.000000382
13	6	-0.000000083	0.000000022	0.000000272
14	6	-0.000000095	0.000000302	-0.000001041
15	6	0.000000053	-0.000000165	0.000000807
16	6	0.000001204	-0.000000636	0.000000411
17	6	-0.000000030	-0.000000054	0.000000205
18	6	0.000000722	-0.000000737	0.000000453
19	6	0.000000392	-0.000000004	-0.000000001
20	8	-0.000000393	-0.000000252	0.000000081
21	6	0.000001025	-0.000000417	0.000001013
22	6	-0.000000187	-0.000000114	0.000000036
23	6	-0.000000188	-0.000000038	-0.000000040
24	6	0.000000236	-0.000000110	-0.000000028
25	6	0.000000429	0.000000077	-0.000000372
26	6	0.000000359	-0.000000514	0.000000016
27	6	-0.000000900	-0.000000320	-0.000000432
28	6	0.000000023	-0.000000265	-0.000000079
29	6	0.000000115	-0.000000033	-0.000000367
30	6	-0.000000433	-0.000000271	-0.000000031
31	6	0.000000139	-0.000000183	-0.000000159
32	6	0.000000043	-0.000000091	-0.000000211
33	6	0.000001226	-0.000000263	-0.000000286
34	6	-0.000000003	0.000000162	-0.000000123
35	6	-0.000000244	-0.000000124	0.000000286
36	6	0.000000132	0.000000132	-0.000000295
37	6	-0.000000037	-0.000000053	-0.000000182
38	6	0.000000050	0.000000154	-0.000000250
39	1	0.000000088	-0.000000313	0.000000166
40	1	0.000000003	-0.000000238	0.000000121
41	1	0.000000276	-0.000000346	-0.000000081
42	1	0.000000146	-0.000000356	-0.000000229
43	1	0.000000089	-0.000000200	-0.000000354
44	1	-0.000000069	-0.000000041	0.000000448
45	1	-0.000000055	-0.000000060	0.000000514
46	1	-0.000000323	-0.000000306	0.000001042
47	1	0.000000039	0.000000976	-0.000000385
48	1	-0.000000038	0.000000029	0.000000200
49	1	0.000000243	0.000000137	0.000000121
50	1	-0.000000677	0.000000207	0.000000480
51	1	0.000000567	-0.000000663	0.000000790
52	1	-0.000000028	-0.000000152	0.000000271
53	1	0.000000152	-0.000000271	-0.000000016
54	1	0.000000142	-0.000000093	-0.000000324
55	1	0.000000098	0.000000009	-0.000000329
56	1	0.000000113	-0.000000271	-0.000000216

57	1	-0.000000260	0.000000622	-0.000000086
58	1	-0.000000387	-0.000000077	0.000000307
59	6	0.000000152	0.000000255	-0.000000024
60	6	-0.000000058	0.000000185	-0.000000241
61	1	-0.000000028	0.000000078	-0.000000213
62	1	-0.000000012	0.000000126	-0.000000181
63	6	-0.000000060	0.000000146	0.000000227
64	1	-0.000000114	-0.000000019	0.000000355
65	1	-0.000000071	0.000000049	0.000000193
66	1	0.000000054	0.000000046	-0.000000118
67	1	-0.000000058	0.000000162	-0.000000269
68	1	-0.000000007	-0.000000014	-0.000000412
69	1	0.000000114	-0.000000123	0.000000109
70	1	-0.000000162	0.000000273	0.000000273
71	1	-0.000000081	0.000000111	0.000000170
72	1	-0.000000168	0.000000192	0.000000195
73	6	-0.000000138	0.000000092	0.000000319
74	1	-0.000000139	0.000000125	0.000000419
75	1	-0.000000093	0.000000043	0.000000308
76	1	-0.000000120	0.000000224	0.000000301
77	1	-0.000000124	0.000000278	-0.000000151
78	1	0.000000033	0.000000193	-0.000000330
79	6	-0.000000040	0.000000321	-0.000000322
80	1	-0.000000042	0.000000278	-0.000000309
81	1	-0.000000130	0.000000284	-0.000000201
82	1	-0.000000085	0.000000327	-0.000000285
83	1	0.000000023	0.000000871	-0.000000475
84	1	-0.000000657	-0.000000399	0.000000176
85	6	-0.000000066	0.000000033	-0.000000041
86	1	-0.000000342	0.000001232	-0.000000491
87	1	-0.000000568	-0.000000271	0.000000034
88	1	0.000000389	0.000000440	-0.000000132

Int2b

Center Number	Atomic Number	Forces (Hartrees/Bohr) X	Y	Z
1	8	-0.000002498	-0.000002425	-0.000000480
2	8	0.000001547	0.000002079	0.000000063
3	6	0.000000427	-0.000001858	-0.000002711
4	8	0.000002909	0.000002167	0.000003041
5	6	-0.000000416	0.000000840	0.000000248
6	6	0.000001676	0.000000542	0.000000083
7	6	0.000001572	-0.000001557	0.000000919
8	6	0.000000153	0.000002236	0.000004560
9	6	-0.000000612	-0.000001058	-0.000001264
10	6	0.000000790	-0.000000008	-0.000000132
11	6	-0.000000696	-0.000000834	0.000000791
12	6	0.000000300	-0.000000513	0.000000004
13	6	0.000000742	0.000000019	-0.000000646
14	6	-0.000001213	0.000000823	-0.000000241
15	6	0.000002403	-0.000001215	-0.000001384

16	6	-0.000000876	0.000000387	0.000002273
17	6	-0.000001247	-0.000000421	-0.000000909
18	6	0.000002179	-0.000002038	0.000001050
19	6	0.000000130	0.000000903	-0.000001145
20	8	0.000000739	0.000000962	0.000002260
21	6	0.000000113	-0.000000153	-0.000000675
22	6	-0.000003004	0.000001198	0.000002135
23	6	-0.000002018	-0.000001044	0.000000074
24	6	-0.000001814	0.000000330	0.000000726
25	6	-0.000001993	0.000000856	0.000001364
26	6	-0.000000795	-0.000000579	-0.000001737
27	6	0.000000098	0.000002886	-0.000000290
28	6	0.000000752	0.000000155	-0.000000608
29	6	-0.000000116	0.000000572	-0.000000447
30	6	0.000001580	-0.000001119	-0.000000673
31	6	0.000000020	-0.000000304	-0.000002424
32	6	0.000000636	-0.000001451	0.000002239
33	6	-0.000001669	-0.000000213	-0.000000590
34	6	-0.000000156	-0.000000708	-0.000000457
35	6	0.000001682	-0.000000604	-0.000000174
36	6	0.000000130	-0.000001781	-0.000000842
37	6	0.000001864	0.000000752	0.000002047
38	6	-0.000001698	0.000002511	0.000004662
39	1	0.000000251	-0.000000030	-0.000000417
40	1	0.000000476	-0.000000286	0.000000616
41	1	0.000000118	0.000000730	-0.000000035
42	1	-0.000000284	-0.000000274	-0.000000009
43	1	0.000000305	-0.000000343	0.000000178
44	1	-0.000000266	0.000000375	-0.000000729
45	1	0.000000548	-0.000000138	-0.000000278
46	1	-0.000000043	-0.000000729	-0.000000851
47	1	-0.000001623	-0.000001808	0.000000535
48	1	-0.000000073	-0.000000173	-0.000000799
49	1	-0.000000568	0.000001161	-0.000002426
50	1	0.000001478	0.000000850	0.000000353
51	1	-0.000000748	0.000000187	-0.000000874
52	1	-0.000000216	0.000000016	-0.000000118
53	1	0.000000508	0.000000190	-0.000000709
54	1	0.000000649	-0.000000186	-0.000000487
55	1	-0.000000541	-0.000000222	0.000000882
56	1	0.000000651	-0.000000159	0.000000334
57	1	0.000000530	0.000001117	-0.000000144
58	1	-0.000000216	0.000002477	-0.000002152
59	6	0.000001427	-0.000002190	-0.000001037
60	6	0.000000949	-0.000000316	0.000001287
61	1	0.000001120	0.000000827	-0.000001312
62	1	0.000000836	0.000000981	-0.000001533
63	6	-0.000000123	-0.000002027	0.000001760
64	1	-0.000000773	0.000000010	0.000000043
65	1	-0.000001577	-0.000001259	-0.000001680
66	1	-0.000000282	-0.000000965	0.000000395
67	1	-0.000000435	-0.000000295	0.000001353
68	1	0.000000402	-0.000000537	-0.000001339
69	1	0.000000176	0.000000740	-0.000000312

70	1	0.000001021	0.000000251	-0.000001604
71	1	-0.000000216	-0.000000039	0.000000731
72	1	-0.000000472	-0.000000365	-0.000000054
73	6	-0.000000258	0.000000721	0.000000650
74	1	-0.000000466	0.000000228	0.000000306
75	1	0.000000072	0.000000138	0.000000387
76	1	-0.000000544	-0.000000167	0.000000215
77	1	-0.000000174	0.000000008	-0.000000245
78	1	-0.000000066	0.000000127	-0.000000041
79	6	-0.000000961	-0.000000318	-0.000000319
80	1	-0.000000273	-0.000000127	-0.000000683
81	1	-0.000000491	0.000000197	-0.000000667
82	1	-0.000000232	0.000000236	-0.000000620
83	1	0.000000455	-0.000000008	0.000000091
84	1	-0.000000076	0.000000493	0.000000451
85	6	-0.000000235	-0.000000128	0.000000338
86	1	-0.000000573	0.000000115	-0.000000284
87	1	-0.000000364	0.000000367	0.000000319
88	1	-0.000000426	0.000000207	-0.000000176

TS3b

Center Number	Atomic Number	Forces (Hartrees/Bohr) X	Y	Z
1	8	0.000000082	-0.000000047	-0.000000192
2	8	-0.000000048	-0.000000024	0.000000173
3	6	-0.000000042	-0.000000152	0.000000152
4	8	-0.000000082	-0.000000073	0.000000002
5	6	-0.000000158	0.000000276	0.000000163
6	6	-0.000000128	0.000000207	0.000000196
7	6	-0.000000091	0.000000115	0.000000239
8	6	-0.000000091	0.000000040	0.000000242
9	6	-0.000000111	-0.000000066	0.000000048
10	6	-0.000000217	0.000000371	-0.000000021
11	6	0.000000117	-0.000000104	-0.000000015
12	6	-0.000000031	0.000000001	0.000000241
13	6	-0.000000032	0.000000254	-0.000000189
14	6	-0.000000046	0.000000231	-0.000000089
15	6	-0.000000027	0.000000184	-0.000000079
16	6	-0.000000039	0.000000147	0.000000042
17	6	0.000000005	0.000000205	-0.000000249
18	6	-0.000000123	0.000000092	0.000000147
19	6	0.000000038	0.000000195	-0.000000265
20	8	-0.000000062	-0.000000044	0.000000320
21	6	0.000000068	0.000000158	-0.000000030
22	6	0.000000038	0.000000089	-0.000000196
23	6	0.000000029	0.000000128	-0.000000248
24	6	0.000000030	0.000000104	-0.000000213
25	6	-0.000000125	0.000000037	0.000000197
26	6	-0.000000119	0.000000233	0.000000111
27	6	-0.000000108	0.000000150	0.000000140
28	6	-0.000000015	0.000000233	-0.000000231

29	6	0.000000168	-0.000000197	-0.000000088
30	6	0.000000068	0.000000004	-0.000000143
31	6	0.000000014	0.000000133	-0.000000237
32	6	-0.000000095	0.000000121	-0.000000099
33	6	0.000000062	0.000000073	-0.000000185
34	6	0.000000097	-0.000000120	-0.000000232
35	6	-0.000000063	-0.000000055	0.000000238
36	6	0.000000028	-0.000000181	0.000000138
37	6	-0.000000021	-0.000000193	0.000000163
38	6	0.000000003	-0.000000143	0.000000212
39	1	-0.000000173	0.000000339	0.000000155
40	1	-0.000000147	0.000000205	0.000000232
41	1	-0.000000088	0.000000127	0.000000053
42	1	-0.000000013	0.000000030	0.000000078
43	1	-0.000000003	-0.000000008	0.000000045
44	1	-0.000000038	0.000000321	-0.000000204
45	1	-0.000000085	0.000000267	-0.000000039
46	1	-0.000000098	0.000000179	0.000000100
47	1	-0.000000056	0.000000066	0.000000101
48	1	0.000000020	0.000000235	-0.000000305
49	1	0.000000110	0.000000036	-0.000000191
50	1	-0.000000049	-0.000000041	0.000000202
51	1	-0.000000139	0.000000028	0.000000310
52	1	-0.000000138	0.000000298	0.000000074
53	1	-0.000000014	0.000000298	-0.000000248
54	1	0.000000063	0.000000058	-0.000000150
55	1	0.000000063	-0.000000028	-0.000000118
56	1	0.000000004	0.000000183	-0.000000167
57	1	0.000000022	-0.000000085	0.000000029
58	1	0.000000099	-0.000000187	0.000000084
59	6	0.000000024	-0.000000123	-0.000000114
60	6	0.000000136	-0.000000223	-0.000000172
61	1	0.000000139	-0.000000103	-0.000000244
62	1	0.000000147	-0.000000100	-0.000000282
63	6	-0.000000042	-0.000000135	0.000000265
64	1	-0.000000104	0.000000001	0.000000265
65	1	-0.000000096	-0.000000029	0.000000310
66	1	-0.000000001	-0.000000171	0.000000176
67	1	0.000000045	-0.000000239	0.000000077
68	1	-0.000000028	-0.000000184	0.000000264
69	1	0.000000008	0.000000261	-0.000000278
70	1	0.000000090	0.000000100	-0.000000300
71	1	-0.000000014	-0.000000179	0.000000245
72	1	-0.000000026	-0.000000157	0.000000174
73	6	-0.000000065	-0.000000167	0.000000351
74	1	-0.000000088	-0.000000147	0.000000359
75	1	-0.000000074	-0.000000180	0.000000410
76	1	-0.000000035	-0.000000255	0.000000332
77	1	0.000000110	-0.000000219	-0.000000189
78	1	0.000000130	-0.000000238	-0.000000114
79	6	0.000000191	-0.000000278	-0.000000255
80	1	0.000000205	-0.000000272	-0.000000265
81	1	0.000000198	-0.000000268	-0.000000316
82	1	0.000000206	-0.000000358	-0.000000239

83	1	0.000000130	-0.000000120	-0.000000162
84	1	0.000000094	-0.000000040	-0.000000167
85	6	0.000000078	-0.000000218	-0.000000073
86	1	0.000000067	-0.000000180	-0.000000073
87	1	0.000000118	-0.000000258	-0.000000034
88	1	0.000000140	-0.000000223	-0.000000157

Int3b

Center Number	Atomic Number	Forces (Hartrees/Bohr) X	Y	Z
1	8	0.000002687	0.000000796	0.000003309
2	8	0.000001502	0.000005252	-0.000002315
3	6	-0.000000438	0.000002395	-0.000001437
4	8	-0.000003440	-0.000000483	-0.000000187
5	6	-0.000000168	-0.000000537	0.000000100
6	6	0.000000759	0.000000039	0.000000358
7	6	-0.000002106	0.000001081	0.000002819
8	6	0.000000676	-0.000001460	-0.000001961
9	6	0.000001835	0.000000550	-0.000001680
10	6	-0.000007705	-0.000001976	0.000000381
11	6	0.000001954	0.000000074	0.000002290
12	6	-0.000002952	-0.000001975	0.000002498
13	6	-0.000000216	-0.000000964	-0.000000494
14	6	0.000000357	0.000001604	-0.000001390
15	6	-0.000001200	0.000000731	0.000000246
16	6	0.000002036	-0.000000966	0.000001307
17	6	0.000000585	-0.000002063	-0.000001151
18	6	-0.000005497	-0.000001626	-0.000003376
19	6	0.000001233	-0.000000551	-0.000003256
20	8	-0.000001959	0.000001389	0.000000213
21	6	0.000003165	-0.000000857	0.000001492
22	6	0.000003736	0.000001940	0.000002872
23	6	-0.000000733	-0.000001228	-0.000001678
24	6	-0.000003312	0.000004328	0.000004195
25	6	-0.000000091	-0.000000085	0.000001322
26	6	0.000000757	-0.000000585	-0.000002142
27	6	0.000002332	0.000000826	0.000001065
28	6	0.000000990	-0.000004012	0.000000990
29	6	0.000004947	0.000004519	0.000001764
30	6	0.000000139	-0.000005232	-0.000004017
31	6	-0.000002414	0.000001641	0.000001423
32	6	0.000000837	-0.000002926	0.000000259
33	6	0.000004058	0.000001786	0.000000030
34	6	-0.000001707	-0.000001552	-0.000000012
35	6	0.000000657	-0.000000324	-0.000001703
36	6	0.000000356	0.000009871	-0.000003689
37	6	0.000000198	-0.000012632	0.000004551
38	6	-0.000002307	0.000007608	-0.000004230
39	1	-0.000000396	0.000000535	-0.000000288
40	1	-0.000000804	0.000000348	-0.000000040
41	1	-0.000001363	0.000000190	0.000000269

42	1	-0.000000929	-0.000000976	0.000000991
43	1	-0.000000698	0.000000270	-0.000000796
44	1	0.000001584	-0.000000016	0.000000452
45	1	0.000000925	0.000000766	0.000000276
46	1	-0.000000192	0.000001593	0.000000089
47	1	-0.000000295	0.000000005	-0.000001455
48	1	0.000001057	-0.000000060	0.000000463
49	1	0.000001423	-0.000000353	-0.000000195
50	1	0.000000922	0.000000839	-0.000000156
51	1	-0.000001589	-0.000000899	-0.000000105
52	1	-0.000000000	0.000000614	-0.000000372
53	1	-0.000000597	-0.000000732	0.000000596
54	1	-0.000000726	-0.000001079	0.000000487
55	1	-0.000002069	-0.000001671	-0.000000442
56	1	-0.000000428	-0.000000878	0.000000500
57	1	-0.000000041	-0.000000465	-0.000000371
58	1	-0.000001158	-0.000001157	-0.000001463
59	6	0.000002227	-0.000002894	-0.000003005
60	6	0.000001769	-0.000001287	0.000001393
61	1	-0.000000471	-0.000000583	0.000000903
62	1	0.000000605	0.000000476	0.000001159
63	6	0.000000190	0.000001759	0.000001333
64	1	-0.000000055	0.000002185	-0.000000408
65	1	-0.000002327	0.000001550	-0.000000757
66	1	-0.000001357	-0.000002174	0.000001886
67	1	0.000000622	-0.000001953	-0.000000169
68	1	-0.000000505	-0.000001343	-0.000000070
69	1	0.000000227	-0.000000254	-0.000000110
70	1	0.000001417	0.000000159	0.000000313
71	1	0.000000432	-0.000000661	-0.000001105
72	1	0.000000818	0.000001653	-0.000001257
73	6	0.000000989	0.000001318	0.000000176
74	1	-0.000000059	0.000001950	-0.000000638
75	1	-0.000000647	0.000000886	-0.000000580
76	1	0.000000031	0.000001245	-0.000001526
77	1	-0.000000930	-0.000000047	0.000000811
78	1	-0.000000414	-0.000001194	-0.000000727
79	6	-0.000001369	-0.000001125	0.000000430
80	1	0.000000603	-0.000001586	0.000001507
81	1	0.000000465	-0.000000712	0.000000670
82	1	0.000000510	-0.000001738	0.000000423
83	1	-0.000000269	0.000000476	0.000000573
84	1	0.000000091	0.000001377	-0.000000039
85	6	0.000001092	0.000001455	0.000001587
86	1	0.000000533	0.000001404	0.000000390
87	1	0.000000141	-0.000000785	-0.000000621
88	1	0.000001462	-0.000000833	0.000000249

Int4b

Center Number	Atomic Number	Forces (Hartrees/Bohr) X	Y	Z

1	8	-0.000001738	-0.000003320	0.000011272
2	8	0.000002707	-0.000010899	-0.000009110
3	8	0.000004026	-0.000003699	0.000010404
4	6	-0.000005466	-0.000002861	0.000002977
5	6	-0.000002776	0.000007565	-0.000010958
6	6	0.000003717	0.000004391	-0.000002152
7	1	0.000000330	-0.000001164	0.000003149
8	1	-0.000000831	0.000001214	-0.000002903
9	6	0.000007930	-0.000004876	0.000013952
10	6	0.000004786	0.000000884	0.000001643
11	1	-0.000000501	-0.000000632	0.000000616
12	6	-0.000003705	-0.000002484	0.000001768
13	8	-0.000008561	0.000004077	-0.000018089
14	6	-0.000003745	0.000009218	-0.000000193
15	6	0.000000060	0.000001936	-0.000001492
16	1	-0.000000667	0.000001437	0.000000405
17	6	-0.000007228	0.000001235	-0.000002977
18	1	0.000000026	0.000000861	0.000000736
19	6	-0.000000686	-0.000000766	0.000002313
20	6	-0.000000017	0.000002298	0.000000142
21	1	0.000000548	0.000000229	-0.000000140
22	6	0.000000800	-0.000004062	-0.000006414
23	1	0.000000357	0.000001146	0.000002923
24	1	-0.000000600	0.000002131	-0.000001340
25	6	0.000004131	-0.000000343	0.000002628
26	1	0.000000570	0.000001886	-0.000001744
27	6	0.000001115	-0.000004485	-0.000005174
28	1	0.000002655	0.000000945	0.000002043
29	1	-0.000002213	0.000000270	0.000001386
30	6	0.000000274	-0.000009028	0.000002782
31	6	-0.000000382	-0.000000466	-0.000005652
32	6	0.000002263	-0.000002384	-0.000003865
33	1	0.000000496	0.000001946	-0.000001224
34	1	-0.000001862	0.000000706	-0.000001208
35	6	0.000003193	0.000009461	0.000005587
36	6	-0.000002104	0.000008558	0.000006896
37	1	0.000000249	-0.000002286	-0.000000086
38	1	0.000000124	-0.000002874	-0.000000848
39	6	0.000000709	-0.000002292	0.000000778
40	6	-0.000004855	-0.000000705	0.000000106
41	1	-0.000001027	-0.000000031	0.000001898
42	1	0.000001586	0.000000601	-0.000002046
43	6	0.000000955	0.000007781	-0.000009615
44	6	0.000003334	-0.000002100	0.000003085
45	6	0.000000676	-0.000007563	0.000011695
46	6	0.000001585	0.000000805	0.000002908
47	1	0.000000008	-0.000000641	0.000000369
48	6	0.000002658	-0.000003754	-0.000006641
49	1	-0.000001377	0.000001928	-0.000002532
50	1	-0.000001280	0.000001716	0.000001442
51	6	-0.000001332	-0.000001258	-0.000000987
52	1	0.000000616	0.000001164	0.000001095
53	1	-0.000000032	0.000000495	-0.000000882
54	1	0.000000355	-0.000000749	-0.000001196

55	6	-0.000001867	0.000000074	0.000000118
56	1	-0.000000018	-0.000000400	0.000000160
57	6	0.000002038	0.000001224	-0.000000873
58	1	0.000001554	0.000000772	0.000002976
59	1	-0.000001182	0.000001002	-0.000000154
60	6	0.000003521	-0.000005854	0.000005804
61	1	-0.000001002	0.000003554	-0.000001557
62	6	-0.000000650	-0.000004701	-0.000007108
63	1	0.000000511	0.000000489	0.000002150
64	1	-0.000000956	-0.000001300	0.000000614
65	6	-0.000000400	-0.000001512	-0.000000086
66	1	-0.000000175	-0.000000332	0.000001376
67	6	-0.000003800	0.000004658	-0.000002185
68	1	-0.000000699	0.000000478	0.000000838
69	6	0.000001478	-0.000001431	-0.000000409
70	1	-0.000000143	0.000000281	0.000000177
71	6	-0.000000028	0.000002018	0.000001185
72	1	0.000000387	0.000000133	-0.000000135
73	6	0.000002999	0.000002266	-0.000000641
74	1	-0.000001222	-0.000001335	-0.000002678
75	1	0.000000211	0.000000710	0.000001060
76	6	-0.000000798	0.000001423	0.000000145
77	1	0.000000530	-0.000001951	0.000000240
78	1	0.000000569	-0.000000212	0.000000703
79	1	-0.000000756	-0.000000760	0.000000438
80	6	-0.000003586	0.000005736	-0.000002372
81	6	0.000003945	0.000005570	0.000001474
82	1	-0.000002046	-0.000000273	-0.000001092
83	1	-0.000000830	0.000000536	-0.000000654
84	6	0.000001053	-0.000000382	-0.000001996
85	1	-0.000001199	-0.000000189	0.000000537
86	1	0.000000170	-0.000000988	-0.000001282
87	1	-0.000000876	-0.000000099	-0.000000817
88	1	0.000003414	-0.000010369	0.000006511

TS5b

Center Number	Atomic Number	Forces (Hartrees/Bohr) X	Y	Z
1	8	0.000003668	0.000002301	0.000000240
2	8	0.000004499	-0.000002300	0.000006129
3	8	-0.000006243	-0.000000200	-0.000002636
4	6	0.000000109	-0.000000286	-0.000001872
5	6	0.000002870	0.000002583	0.000002112
6	6	-0.000000265	0.000001638	-0.000002751
7	1	-0.000000302	0.000000365	0.000005345
8	1	0.000000789	0.000000451	0.000000110
9	6	-0.000002242	-0.000000835	0.000000427
10	6	0.000000222	0.000000429	0.000000320
11	1	0.000000265	0.000000474	0.000000174
12	6	0.000001549	-0.000000105	0.000000681
13	8	0.000002580	0.000003788	-0.000003610

14	6	-0.000000162	-0.000002757	0.000004577
15	6	-0.000000776	-0.000002005	0.000002863
16	1	-0.000000069	0.000000195	0.000000319
17	6	-0.000001327	-0.000000366	0.000000133
18	1	-0.000000507	0.000000224	0.000000348
19	6	-0.000000750	0.000001354	-0.000001337
20	6	-0.000000238	0.000000425	0.000000627
21	1	-0.000000122	0.000000374	0.000000275
22	6	0.000000387	0.000003206	-0.000000660
23	1	-0.000000567	-0.000002249	0.000002403
24	1	0.000000472	0.000000760	0.000000484
25	6	0.000000630	0.000000888	0.000000199
26	1	0.000000342	0.000000339	0.000000211
27	6	-0.000010499	-0.000002439	-0.000004097
28	1	0.000000808	-0.000001219	0.000002985
29	1	0.000001733	0.000000280	0.000001440
30	6	0.000002002	-0.000001208	-0.000000905
31	6	0.000001232	0.000002935	-0.000001633
32	6	0.000002339	0.000001891	0.000004585
33	1	-0.000000581	0.000000898	-0.000000109
34	1	0.000002140	0.000000260	0.000001186
35	6	-0.000000587	0.000003249	0.000000149
36	6	-0.000004541	0.000006123	-0.000016306
37	1	0.000000888	-0.000004459	0.000004367
38	1	0.000000532	-0.000004415	0.000001730
39	6	-0.000000187	-0.000000869	0.000000191
40	6	0.000003360	0.000001738	-0.000001028
41	1	-0.000001988	-0.000000671	0.000002084
42	1	0.000000170	0.000001029	0.000000410
43	6	-0.000000464	-0.000000607	0.000000398
44	6	-0.000000813	0.000000822	0.000000490
45	6	-0.000001531	-0.000000810	-0.000000171
46	6	-0.000000204	-0.000000209	-0.000000429
47	1	-0.000000627	-0.000000041	0.000000436
48	6	-0.000000411	0.000001685	-0.000006630
49	1	-0.000000759	0.000000082	-0.000000855
50	1	0.000001946	-0.000003996	0.000004892
51	6	0.000001340	0.000000631	-0.000000324
52	1	-0.000001469	0.000000103	0.000001331
53	1	-0.000000347	0.000000013	-0.000001168
54	1	0.000000931	0.000000640	0.000000916
55	6	-0.000000828	-0.000000564	-0.000000542
56	1	-0.000000417	-0.000000379	-0.000000099
57	6	0.000001342	0.000000976	0.000001456
58	1	0.000001359	0.000002096	0.000000351
59	1	-0.000001156	-0.000004174	-0.000003616
60	6	-0.000001897	0.000001607	0.000000304
61	1	0.000000679	0.000000838	-0.000000309
62	6	0.000000661	-0.000000180	-0.000003132
63	1	-0.000000035	-0.000000136	0.000000738
64	1	0.000000422	-0.000001300	0.000000539
65	6	-0.000000280	-0.000000689	-0.000000127
66	1	-0.000000615	-0.000000233	0.000000355
67	6	-0.000000707	0.000000127	0.000000005

68	1	-0.000000273	-0.000000305	-0.000000150
69	6	0.000000733	-0.000000100	-0.000000596
70	1	0.000000511	0.000000108	-0.000000336
71	6	0.000000704	-0.000000190	-0.000000254
72	1	0.000000345	0.000000081	-0.000000250
73	6	-0.000001855	-0.000000905	-0.000001052
74	1	-0.000000652	-0.000001235	-0.000001242
75	1	0.000000452	0.000000207	0.000000612
76	6	0.000000088	0.000002308	0.000000538
77	1	-0.000000563	-0.000001255	0.000000208
78	1	-0.000000008	-0.000000852	-0.000000010
79	1	-0.000000137	0.000000371	0.000000096
80	1	-0.000002629	-0.000006863	-0.000000344
81	6	-0.000000478	0.000000767	-0.000001443
82	1	0.000001020	0.000000130	-0.000000196
83	1	0.000001414	0.000000834	-0.000001007
84	6	-0.000000211	-0.000000759	-0.000001369
85	1	0.000000651	0.000000067	0.000000195
86	1	-0.000000622	-0.000000983	-0.000000329
87	1	-0.000000204	-0.000000629	-0.000001183
88	6	0.000003964	0.000001082	0.000003142

Int5b

Center Number	Atomic Number	Forces (Hartrees/Bohr) X	Y	Z
1	8	-0.000008301	0.000005460	0.000001974
2	8	-0.000001710	-0.000004319	0.000001520
3	8	-0.000001456	0.000001071	-0.000005162
4	6	0.000000148	0.000003049	-0.000003798
5	6	0.000005321	0.000005159	-0.000004737
6	6	0.000001729	0.000001852	-0.000001098
7	1	-0.000000579	-0.000001559	-0.000000836
8	1	-0.000001068	-0.000001044	0.000000878
9	6	0.000001328	-0.000007911	0.000005702
10	6	0.000001104	-0.000004924	0.000001397
11	1	-0.000000589	0.000000455	-0.000000742
12	6	-0.000001948	-0.000004306	-0.000001291
13	8	-0.000001784	-0.000001833	0.000000543
14	6	0.000000100	0.000007873	-0.000002906
15	6	0.000000311	0.000003781	0.000001787
16	1	0.000000296	-0.000001339	-0.000000980
17	6	-0.000002938	-0.000003009	-0.000005546
18	1	0.000000486	-0.000000759	0.000000044
19	6	-0.000001909	-0.000002957	0.000002677
20	6	-0.000000073	0.000003655	0.000000544
21	1	-0.000000232	-0.000000338	-0.000000454
22	6	0.000002322	0.000005342	0.000001433
23	1	0.000001334	-0.000000331	-0.000003263
24	1	-0.000000058	-0.000001501	0.000000370
25	6	0.000000269	0.000001960	0.000000836
26	1	-0.000000722	0.000000523	-0.000000422

27	6	0.000006155	0.000000920	-0.000004749
28	1	-0.000001826	-0.000000200	0.000001120
29	1	-0.000002190	-0.000001531	0.000001081
30	6	-0.000000937	-0.000000426	-0.000002027
31	6	-0.000001552	-0.000005844	-0.000000464
32	6	0.000000923	-0.000002103	0.000002926
33	1	-0.000001364	0.000000333	0.000000926
34	1	0.000002096	0.000002582	0.000000313
35	6	0.000002771	0.000000916	0.000000753
36	6	0.000001163	0.000002723	-0.000002962
37	1	-0.000000518	-0.000001928	0.000001649
38	1	0.000000504	-0.000000696	-0.000000194
39	6	0.000000868	0.000000080	0.000000687
40	6	-0.000001642	0.000000257	0.000002757
41	1	0.000001622	-0.000000495	-0.000001311
42	1	0.000000399	0.000000321	-0.000003094
43	6	-0.000000025	-0.000000929	0.000003027
44	6	0.000001726	-0.000000251	-0.000001549
45	6	-0.000001777	-0.000001531	-0.000000091
46	6	-0.000002652	0.000003253	-0.000000931
47	1	0.000000347	-0.000000375	-0.000000309
48	6	-0.000001448	-0.000000783	0.000004506
49	1	0.000002141	0.000000122	-0.000000585
50	1	0.000001423	0.000001455	-0.000000671
51	6	0.000001173	-0.000001826	-0.000001449
52	1	-0.000000150	0.000000942	-0.000000949
53	1	0.000001558	0.000000926	0.000000128
54	1	-0.000000348	0.000000487	0.000001164
55	6	-0.000000675	-0.000001436	-0.000000086
56	1	0.000000643	0.000000222	0.000000090
57	6	-0.000001193	-0.000001087	-0.000001791
58	1	-0.000000683	-0.000000211	-0.000000163
59	1	-0.000000047	-0.000000001	0.000003118
60	6	0.000002017	0.000003155	0.000001132
61	1	-0.000001371	-0.000000490	-0.000000316
62	6	-0.000002820	-0.000001988	-0.000002532
63	1	0.000000579	-0.000000444	0.000003133
64	1	0.000001904	0.000001800	0.000002250
65	6	0.000002202	0.000000096	0.000000030
66	1	0.000000054	0.000000262	-0.000000159
67	6	0.000000516	-0.000000732	-0.000002310
68	1	0.000000015	0.000000143	0.000000319
69	6	0.000000953	-0.000000800	0.000001572
70	1	-0.000000642	0.000000059	0.000000433
71	6	-0.000001737	0.000001878	0.000001712
72	1	-0.000000339	-0.000000516	0.000000109
73	6	0.000000190	0.000002342	0.000003647
74	1	-0.000000008	0.000000565	-0.000001617
75	1	-0.000000146	-0.000001824	-0.000000421
76	6	0.000000978	0.000000823	0.000001078
77	1	0.000000029	0.000001233	0.000000148
78	1	-0.000000053	-0.000000755	-0.000000502
79	1	0.000001624	0.000000508	-0.000000713
80	6	-0.000000999	-0.000002827	-0.000001813

81	1	0.000000087	0.000000505	0.000000934
82	6	-0.000001786	0.000001032	0.000001770
83	1	-0.000000320	-0.000001171	-0.000000238
84	1	-0.000000413	-0.000000494	0.000000015
85	6	0.000001987	-0.000001590	0.000001029
86	1	0.000000194	0.000000991	-0.000000429
87	1	0.000000378	0.000000840	0.000001563
88	1	-0.000000940	-0.000000537	0.000000837

TS6b

Center Number	Atomic Number	Forces (Hartrees/Bohr) X	Y	Z
1	8	0.000001771	0.000003031	-0.000001988
2	8	-0.000004588	-0.000000751	-0.000003546
3	8	-0.000003447	-0.000001885	-0.000000304
4	6	-0.000000728	-0.000001888	0.000001119
5	6	-0.000005434	-0.000004176	0.000000524
6	6	0.000000403	0.000000619	0.000000562
7	1	0.000002771	-0.000000303	0.000000919
8	1	-0.000000989	-0.000001327	-0.000002927
9	6	0.000000127	0.000001152	-0.000001238
10	6	0.000003880	0.000001679	-0.000000365
11	1	0.000000405	-0.000000444	0.000000377
12	6	-0.000002860	-0.000000161	-0.000000169
13	8	-0.000003555	-0.000002594	-0.000004566
14	6	0.000000550	0.000003035	0.000002698
15	6	0.000001318	0.000002497	-0.000000512
16	1	-0.000000502	0.000000294	0.000002006
17	6	0.000001920	0.000001236	-0.000000127
18	1	0.000000465	-0.000000001	-0.000000054
19	6	0.000001552	-0.000001776	-0.000001150
20	6	0.000000131	0.000000099	0.000000243
21	1	0.000000510	-0.000000103	0.000000164
22	6	0.000001077	-0.000000601	0.000000674
23	1	0.000001999	-0.000001203	0.000000514
24	1	-0.000000817	0.000000905	0.000000230
25	6	-0.000002046	0.000001415	-0.000000016
26	1	-0.000000242	-0.000000341	0.000000123
27	6	0.000002778	0.000000386	-0.000003924
28	1	-0.000000788	0.000000212	-0.000000508
29	1	-0.000000817	-0.000000378	0.000000254
30	6	0.000000880	-0.000005229	-0.000002263
31	6	-0.000003546	0.000002353	-0.000006978
32	6	0.000000993	-0.000002797	0.000000787
33	1	0.000000184	-0.000000359	0.000001232
34	1	0.000001429	0.000001212	-0.000000643
35	6	0.000006701	0.000004832	0.000006626
36	6	0.000002414	-0.000002332	0.000003710
37	1	-0.000000317	0.000001165	-0.000002006
38	1	-0.000000960	0.000001123	-0.000000332
39	6	0.000001847	-0.000000818	-0.000002645

40	6	-0.000001425	0.000001081	0.000005950
41	1	0.000001729	-0.000001292	0.000000351
42	1	0.000000373	-0.000001635	0.000000856
43	6	0.000004965	0.000002477	0.000000533
44	6	0.000001758	-0.000001453	-0.000000035
45	6	0.000001209	0.000000840	-0.000000105
46	6	0.000000326	-0.000000375	-0.000000707
47	1	0.000000746	0.000000132	0.000000156
48	6	-0.000000144	0.000000620	-0.000000092
49	1	0.000000229	0.000002334	-0.000001169
50	1	-0.000000896	-0.000001423	0.000000906
51	6	0.000002323	0.000000536	0.000001125
52	1	0.000000173	-0.000000586	0.000000402
53	1	-0.000001276	-0.000000914	-0.000000197
54	1	-0.000000766	-0.000001255	-0.000000272
55	6	0.000000683	0.000000416	-0.000000124
56	1	0.000000453	0.000000473	-0.000000173
57	6	-0.000000067	0.000000826	0.000003678
58	1	-0.000001442	0.000000620	0.000000551
59	1	0.000000591	-0.000000731	-0.000001035
60	6	-0.000003029	-0.000011789	0.000012382
61	1	-0.000001281	-0.000003290	0.000000397
62	6	-0.000001785	-0.000003109	0.000001994
63	1	0.000001259	0.000003253	0.000000738
64	1	-0.000002936	-0.000000271	0.000000278
65	6	0.000000363	0.000000467	-0.000000685
66	1	0.000000707	0.000000367	-0.000000247
67	6	-0.000002277	-0.000000190	-0.000000061
68	1	0.000000168	0.000000487	-0.000000136
69	6	-0.000001512	-0.000000738	0.000000032
70	1	-0.000000189	0.000000230	-0.000000021
71	6	0.000000601	0.000000481	-0.000000363
72	1	0.000000361	0.000000476	-0.000000153
73	6	0.000000267	0.000000384	-0.000004360
74	1	-0.000000356	-0.000001592	-0.000000626
75	1	-0.000001984	0.000002667	-0.000000870
76	6	0.000000154	0.000000722	-0.000000460
77	1	-0.000000265	0.000000029	0.000000180
78	1	-0.000000256	-0.000000012	0.000000600
79	1	-0.000000376	0.000000073	0.000000428
80	6	0.000009410	0.000012157	-0.000005414
81	1	0.000000642	0.000000237	-0.000001771
82	6	-0.000012312	0.000000555	0.000004215
83	1	0.000002691	-0.000002390	-0.000002232
84	1	-0.000000326	0.000000216	-0.000000084
85	6	0.000001363	0.000001990	-0.000000807
86	1	-0.000001106	-0.000000210	-0.000000349
87	1	-0.000001115	0.000000411	-0.000001173
88	1	-0.000000894	-0.000000081	0.000001471

2

Center Atomic Forces (Hartrees/Bohr)

Number	Number	X	Y	Z
1	8	-0.000001699	0.000001694	0.000003075
2	8	0.000001475	0.000004085	0.000000209
3	8	-0.000004222	-0.000000668	0.000002202
4	6	-0.000001211	0.000000222	0.000002100
5	6	0.000000654	-0.000003356	-0.000003466
6	6	-0.000001303	-0.000000119	-0.000001050
7	1	0.000000133	0.000001000	0.000000534
8	1	0.000001736	-0.000000158	-0.000001128
9	6	0.000001089	0.000000504	-0.000000793
10	6	-0.000000518	0.000001284	-0.000000167
11	1	0.000000017	0.000000154	-0.000000242
12	6	-0.000000570	-0.000001687	-0.000000294
13	8	-0.000001561	-0.000002103	-0.000002618
14	6	0.000001781	-0.000002301	-0.000000566
15	6	0.000000084	-0.000000623	0.000000076
16	1	-0.000000081	-0.000000078	-0.000000290
17	6	-0.000000288	-0.000000476	0.000000174
18	1	-0.000000278	-0.000000110	0.000000127
19	6	0.000000038	0.000002812	-0.000002342
20	6	0.000000074	-0.000000063	0.000000151
21	1	-0.000000269	-0.000000057	-0.000000228
22	6	-0.000000013	-0.000002275	0.000000682
23	1	0.000000302	-0.000000888	0.000001615
24	1	-0.000001512	0.000000381	-0.000000273
25	6	0.000000073	0.000001736	0.000000251
26	1	0.000000017	0.000000085	0.000000127
27	6	-0.000000748	-0.000001440	-0.000000522
28	1	0.000000473	0.000001199	-0.000000336
29	1	0.000000439	-0.000000144	0.000000438
30	6	0.000002657	0.000002001	-0.000000405
31	6	-0.000002239	-0.000000193	-0.000000307
32	6	0.000000211	0.000001087	-0.000000851
33	1	0.000000202	0.000000423	0.000000178
34	1	-0.000000135	-0.000000875	0.000000373
35	6	0.000001757	-0.000002163	0.000000990
36	6	-0.000005013	-0.000003322	0.000002968
37	1	0.000000641	0.000000084	-0.000001392
38	1	-0.000000093	0.000000610	0.000000438
39	6	0.000001737	0.000001750	-0.000001493
40	6	0.000000846	0.000001239	0.000000857
41	1	-0.000000658	-0.000001695	-0.000000144
42	1	-0.000001034	-0.000001184	0.000000390
43	6	0.000005016	-0.000002117	-0.000003047
44	6	-0.000001363	0.000000537	0.000000200
45	6	-0.000000720	-0.000000074	0.000001780
46	6	0.000000105	0.000000293	0.000001604
47	1	-0.000000359	-0.000000268	0.000000076
48	6	0.000001264	0.000001484	0.000001441
49	1	0.000000175	-0.000000327	-0.000000710
50	1	0.000000155	-0.000001867	0.000000315
51	6	-0.000000504	0.000000176	-0.000001047
52	1	-0.000000287	0.000000169	0.000000081

53	1	-0.000000038	-0.000000287	-0.000000372
54	1	0.000000447	0.000000025	0.000000555
55	6	-0.000001323	-0.000000440	-0.000000524
56	1	0.000000077	-0.000000248	0.000000071
57	6	-0.000000639	0.000002238	0.000001546
58	1	-0.000001202	0.000000061	-0.000001654
59	1	-0.000000246	-0.000001320	0.000001525
60	6	0.000000880	0.000000517	-0.000001355
61	1	-0.000000281	0.000000137	0.000000153
62	6	-0.000001061	0.000000023	-0.000001455
63	1	0.000000992	0.000000454	0.000000213
64	1	0.000001268	0.000000298	0.000000709
65	6	0.000000386	-0.000001126	0.000000453
66	1	-0.000000218	-0.000000355	0.000000124
67	6	0.000000203	-0.000000142	-0.000000643
68	1	-0.000000213	-0.000000297	0.000000040
69	6	-0.000001244	0.000000903	-0.000000371
70	1	-0.000000066	0.000000482	-0.000000561
71	6	0.000000092	-0.000000092	-0.000000124
72	1	0.000000150	-0.000000043	0.000000108
73	6	0.000002916	0.000000261	0.000001697
74	1	-0.000000937	-0.000000028	0.000000023
75	1	-0.000000818	0.000000610	-0.000000437
76	6	0.000001957	0.000000015	-0.000000283
77	1	-0.000000191	-0.000000030	0.000000692
78	1	-0.000000530	-0.000000034	0.000000043
79	1	0.000000046	-0.000000359	0.000000318
80	6	0.000000089	0.000001099	-0.000001203
81	1	0.000000497	0.000000434	-0.000000705
82	6	0.000002720	0.000003616	0.000002071
83	1	-0.000000345	-0.000000428	0.000000342
84	1	-0.000000979	-0.000000169	-0.000000849
85	6	0.000000237	-0.000001145	-0.000000293
86	1	0.000000288	0.000000041	0.000000412
87	1	0.000000727	0.000000597	0.000000195
88	1	-0.000000115	0.000000357	-0.000000200

5

Center Number	Atomic Number	Forces (Hartrees/Bohr) X	Y	Z
1	8	-0.000005335	0.000001662	0.000002676
2	8	0.000001100	-0.000002472	-0.000003061
3	8	-0.000001019	-0.000001547	-0.000002171
4	6	-0.000000314	0.000000104	-0.000002366
5	6	0.000006781	-0.000005855	-0.000001083
6	6	-0.000004647	0.000000746	0.000001111
7	1	-0.000001190	0.000000831	0.000001476
8	1	0.000000704	0.000001802	-0.000001039
9	6	0.000000792	0.000000467	-0.000001048
10	6	-0.000000172	0.000001812	-0.000000562
11	1	-0.000000061	0.000000295	0.000000010

12	6	0.000000721	-0.000005869	-0.000002746
13	8	-0.000000679	-0.000000805	0.000003452
14	6	0.000004230	0.000001116	0.000003450
15	6	-0.000002673	0.000001749	-0.000001124
16	1	0.000000391	0.000000181	0.000000331
17	6	-0.000000787	0.000000957	-0.000001218
18	1	0.000000048	-0.000000902	-0.000000451
19	6	-0.000001296	-0.000000340	-0.000000539
20	6	-0.000000899	-0.000001279	0.000001813
21	1	-0.000000016	-0.000000146	0.000000126
22	6	0.000002122	-0.000001257	0.000002993
23	1	-0.000001330	-0.000000046	-0.000001307
24	1	0.000000069	0.000000051	-0.000001085
25	6	0.000000805	0.000002271	-0.000000042
26	1	-0.000000367	0.000000179	0.000000093
27	6	0.000000894	-0.000001513	-0.000003742
28	1	-0.000001511	0.000000360	-0.000000467
29	1	-0.000001282	0.000000638	0.000000350
30	6	0.000002045	0.000002851	0.000004065
31	6	-0.000000651	0.000002869	0.000000997
32	6	0.000002844	-0.000000367	-0.000002740
33	1	-0.000000800	0.000000818	0.000000872
34	1	-0.000000466	-0.000000947	-0.000000234
35	6	0.000001122	-0.000001901	-0.000002061
36	6	-0.000004512	-0.000000618	0.000004995
37	1	0.000001333	-0.000001524	-0.000000226
38	1	0.000000420	-0.000000936	-0.000000965
39	6	0.000001350	-0.000002688	-0.000000369
40	6	-0.000004085	-0.000000644	0.000002611
41	1	0.000000329	-0.000000101	0.000000257
42	1	0.000001544	0.000001067	-0.000000499
43	6	-0.000002532	0.000001148	0.000004205
44	6	-0.000001250	-0.000002086	0.000000115
45	6	0.000002954	-0.000000550	-0.000005164
46	6	-0.000002447	0.000001341	0.000000764
47	1	0.000000358	-0.000000547	-0.000000811
48	6	0.000002672	0.000003370	0.000001355
49	1	0.000000486	-0.000000599	-0.000000428
50	1	-0.000001006	-0.000000831	0.000001696
51	6	-0.000000023	0.000000550	0.000001368
52	1	-0.000000938	-0.000000570	-0.000000743
53	1	-0.000001273	0.000001033	-0.000000583
54	1	0.000000169	0.000000221	-0.000000140
55	6	-0.000000012	0.000000708	-0.000001060
56	1	0.000000687	-0.000000258	0.000001790
57	6	-0.000000442	0.000001770	-0.000001609
58	1	0.000001138	0.000001138	-0.000000556
59	1	0.000000443	-0.000000242	0.000000698
60	6	-0.000000359	0.000000908	-0.000003532
61	1	-0.000000325	0.000000491	0.000000394
62	6	-0.000001290	-0.000000473	0.000002152
63	1	0.000002669	0.000001030	-0.000000442
64	1	0.000002317	-0.000001567	-0.000000102
65	6	0.000000289	-0.000002316	0.000000284

66	1	-0.000000206	0.000001190	-0.000000153
67	6	-0.000003600	-0.000000691	0.000002263
68	1	-0.000000514	-0.000000201	-0.000000384
69	6	0.000001151	-0.000000268	-0.000000681
70	1	0.000000258	0.000000792	-0.000000186
71	6	0.000001546	0.000001489	0.000001475
72	1	0.000000106	0.000000560	0.000000120
73	6	0.000002686	0.000000232	0.000000633
74	1	-0.000001033	0.000000994	-0.000000716
75	1	-0.000001169	0.000000164	-0.000001290
76	6	-0.000000428	0.000000349	0.000000391
77	1	0.000000314	-0.000000282	0.000000506
78	1	-0.000000598	-0.000000465	-0.000000076
79	1	0.000000546	-0.000000896	0.000000154
80	6	0.000001284	0.000000200	0.000002410
81	1	-0.000001223	0.000000991	-0.000000636
82	6	-0.000001207	0.000001044	-0.000004289
83	1	0.000000814	0.000000016	0.000000020
84	1	0.000000534	0.000000491	-0.000000593
85	6	0.000001477	-0.000001425	-0.000000381
86	1	0.000000510	0.000000437	0.000000625
87	1	0.000000744	0.000000028	0.000000443
88	1	0.000000169	0.000000514	0.000000161

TS3b_p

Center Number	Atomic Number	Forces (Hartrees/Bohr) X	Y	Z
1	8	-0.000000297	-0.000000122	-0.000000450
2	8	0.000000300	0.000000129	0.000000441
3	6	-0.000000209	0.000000571	0.000000451
4	8	0.000000210	-0.000000259	-0.000000054
5	6	-0.000000157	0.000000241	0.000000117
6	6	-0.000000191	0.000000392	0.000000215
7	6	-0.000000015	0.000000367	0.000000304
8	6	-0.000000182	0.000000460	0.000000273
9	6	-0.000000409	0.000000569	0.000000152
10	6	-0.000000358	0.000000421	0.000000089
11	6	-0.000000523	0.000000555	0.000000151
12	6	-0.000000237	0.000000473	0.000000257
13	6	0.000000211	-0.000000632	-0.000000463
14	6	0.000000280	-0.000000477	-0.000000241
15	6	0.000000310	-0.000000375	-0.000000172
16	6	0.000000345	-0.000000232	0.000000157
17	6	0.000000113	-0.000000605	-0.000000553
18	6	0.000000102	0.000000138	0.000000277
19	6	-0.000000001	-0.000000370	-0.000000398
20	8	0.000000057	0.000000469	0.000000560
21	6	0.000000199	-0.000000415	-0.000000156
22	6	0.000000235	-0.000000527	-0.000000494
23	6	0.000000024	-0.000000504	-0.000000381
24	6	-0.000000044	-0.000000314	-0.000000461

25	6	-0.000000102	0.000000531	0.000000463
26	6	-0.000000003	0.000000050	0.000000054
27	6	0.000000160	-0.000000004	0.000000198
28	6	-0.000000106	-0.000000184	-0.000000320
29	6	-0.000000669	0.000000254	-0.000000429
30	6	-0.000000222	-0.000000164	-0.000000426
31	6	-0.000000296	-0.000000024	-0.000000323
32	6	-0.000000351	0.000000016	-0.000000333
33	6	0.000000420	-0.000000373	0.000000040
34	6	-0.000000413	-0.000000205	-0.000000656
35	6	0.000000405	0.000000153	0.000000618
36	6	-0.000000351	0.000000550	0.000000223
37	6	-0.000000268	0.000000534	0.000000308
38	6	-0.000000197	0.000000514	0.000000388
39	1	-0.000000270	0.000000276	0.000000024
40	1	-0.000000330	0.000000550	0.000000250
41	1	-0.000000460	0.000000500	0.000000068
42	1	-0.000000678	0.000000553	-0.000000107
43	1	-0.000000547	0.000000542	0.000000022
44	1	0.000000198	-0.000000710	-0.000000575
45	1	0.000000327	-0.000000479	-0.000000188
46	1	0.000000432	-0.000000261	0.000000188
47	1	0.000000453	-0.000000231	0.000000234
48	1	0.000000078	-0.000000719	-0.000000715
49	1	0.000000221	-0.000000635	-0.000000468
50	1	0.000000059	0.000000500	0.000000593
51	1	-0.000000200	0.000000668	0.000000525
52	1	0.000000039	-0.000000074	-0.000000034
53	1	-0.000000072	-0.000000211	-0.000000309
54	1	-0.000000652	0.000000278	-0.000000360
55	1	-0.000000580	0.000000209	-0.000000394
56	1	-0.000000385	0.000000112	-0.000000277
57	1	0.000000458	-0.000000502	-0.000000088
58	1	0.000000558	-0.000000400	0.000000147
59	6	0.000000392	-0.000000219	0.000000171
60	6	-0.000000462	-0.000000160	-0.000000683
61	1	-0.000000333	-0.000000373	-0.000000763
62	1	-0.000000562	-0.000000144	-0.000000745
63	6	0.000000552	0.000000145	0.000000739
64	1	0.000000533	0.000000059	0.000000633
65	1	0.000000358	0.000000328	0.000000716
66	1	-0.000000469	0.000000686	0.000000261
67	1	-0.000000311	0.000000402	0.000000115
68	1	-0.000000040	0.000000525	0.000000533
69	1	0.000000135	-0.000000496	-0.000000405
70	1	-0.000000016	-0.000000619	-0.000000674
71	1	0.000000452	0.000000248	0.000000740
72	1	0.000000634	-0.000000015	0.000000654
73	6	0.000000710	0.000000202	0.000000974
74	1	0.000000830	0.000000102	0.000000980
75	1	0.000000643	0.000000358	0.000001071
76	1	0.000000813	0.000000185	0.000001053
77	1	-0.000000556	0.000000010	-0.000000578
78	1	-0.000000323	-0.000000241	-0.000000596

79	6	-0.000000614	-0.000000237	-0.000000896
80	1	-0.000000533	-0.000000402	-0.000001000
81	1	-0.000000760	-0.000000169	-0.000000982
82	1	-0.000000653	-0.000000197	-0.000000901
83	1	0.000000363	-0.000000079	0.000000295
84	1	0.000000273	-0.000000183	0.000000080
85	6	0.000000591	-0.000000304	0.000000290
86	1	0.000000630	-0.000000448	0.000000174
87	1	0.000000732	-0.000000339	0.000000398
88	1	0.000000575	-0.000000193	0.000000387

TS2_claisen

Center Number	Atomic Number	Forces (Hartrees/Bohr) X	Y	Z
1	8	-0.000002694	0.000001342	-0.000000244
2	8	-0.000004246	0.000001262	-0.000000281
3	6	-0.000011546	-0.000022259	-0.000004496
4	8	0.000000685	0.000002922	-0.000000737
5	6	-0.000000085	-0.000000293	-0.000000387
6	6	-0.000000094	-0.000001987	0.000000014
7	6	-0.000000447	-0.000002758	0.000000261
8	6	-0.000006513	0.000000471	0.000003051
9	6	0.000001768	-0.000000006	-0.000001049
10	6	-0.000029659	0.000022456	0.000001756
11	6	0.000001136	-0.000033383	-0.000044794
12	6	0.000001330	0.000022234	0.000030392
13	6	0.000000144	0.000000779	-0.000000389
14	6	0.000000574	0.000000431	-0.000001164
15	6	-0.000000365	-0.000000059	-0.000001548
16	6	-0.000001139	-0.000001698	-0.000000218
17	6	0.000000540	0.000000477	-0.000000424
18	6	0.000001680	0.000001109	0.000000247
19	6	0.000000583	0.000002937	-0.000000546
20	8	0.000006536	0.000000008	0.000012051
21	6	-0.000000869	-0.000001972	0.000000711
22	6	0.000001170	-0.000000288	0.000000616
23	6	0.000000303	-0.000000282	0.000000925
24	6	0.000000167	0.000000033	0.000000311
25	6	-0.000003563	0.000002395	-0.000003112
26	6	0.000000273	0.000001518	-0.000000088
27	6	0.000000200	0.000003271	-0.000001161
28	6	0.000000725	0.000000185	0.000000162
29	6	0.000005660	0.000015414	-0.000005747
30	6	0.000000345	-0.000007584	0.000000243
31	6	0.000000683	0.000000939	-0.000000477
32	6	0.000002659	0.000007564	-0.000002280
33	6	-0.000000351	-0.000001151	0.000000151
34	6	0.000001396	-0.000000293	0.000000054
35	6	0.000001262	0.000000369	-0.000000221
36	6	-0.000001937	0.000014445	0.000027808
37	6	0.000062782	0.000011395	-0.000036080

38	6	-0.000037880	-0.000034849	-0.000000301
39	1	0.000000166	0.000000282	-0.000000104
40	1	-0.000000021	-0.000000501	0.000000012
41	1	0.000002905	-0.000000675	0.000003222
42	1	-0.000004356	0.000000820	0.000002531
43	1	0.000001911	-0.000001808	0.000000192
44	1	0.000000402	0.000000613	-0.000000686
45	1	0.000000092	0.000000519	-0.000000551
46	1	-0.000000131	-0.000000306	0.000001321
47	1	0.000001010	0.000001551	-0.000000355
48	1	0.000000430	0.000000472	-0.000000409
49	1	-0.000000004	0.000002769	-0.000000328
50	1	0.000000007	-0.000002984	0.000000375
51	1	0.000000201	0.000000472	-0.000002754
52	1	0.000000245	0.000000715	-0.000000296
53	1	0.000000633	0.000000258	-0.000000054
54	1	0.000002982	-0.000000973	0.000003774
55	1	-0.000007440	-0.000001655	-0.000001902
56	1	0.000000380	0.000000502	0.000000251
57	1	0.000001032	0.000000756	-0.000000600
58	1	-0.000001083	0.000001063	-0.000000170
59	6	-0.000000638	-0.000000754	0.000000012
60	6	0.000000727	0.000000300	-0.000000006
61	1	0.000000510	-0.000000805	-0.000000045
62	1	0.000000819	0.000000795	0.000000199
63	6	-0.000000269	-0.000000213	-0.000000072
64	1	-0.000000720	0.000000182	0.000000270
65	1	-0.000000757	0.000000058	-0.000000608
66	1	-0.000001815	0.000002987	0.000004495
67	1	0.000015734	-0.000008027	0.000020908
68	1	0.000001660	0.000000749	0.000000558
69	1	0.000000472	0.000000249	-0.000000253
70	1	-0.000000686	-0.000001046	-0.000002247
71	1	-0.000000543	-0.000000087	0.000000332
72	1	-0.000000714	-0.000000112	-0.000000740
73	6	-0.000000565	0.000000157	-0.000000014
74	1	-0.000000872	-0.000000637	0.000000662
75	1	-0.000000386	-0.000000376	-0.000000666
76	1	-0.000000973	0.000000531	0.000000029
77	1	-0.000000860	-0.000000054	0.000000387
78	1	0.000000459	-0.000001036	-0.000000091
79	6	0.000000417	0.000000065	-0.000000098
80	1	-0.000000096	-0.000000136	0.000000516
81	1	0.000000102	-0.000000326	0.000000119
82	1	0.000000154	0.000000121	0.000000202
83	1	0.000000187	0.000000453	-0.000000370
84	1	-0.000000269	0.000000047	0.000000536
85	6	-0.000000413	-0.000000281	-0.000000145
86	1	-0.000000328	0.000000108	-0.000000140
87	1	-0.000000495	0.000000120	-0.000000146
88	1	-0.000000416	-0.000000017	-0.000000065

TS2_sigma

Center Number	Atomic Number	Forces (Hartrees/Bohr) X	Y	Z
1	8	0.000119986	0.000730632	-0.000484660
2	8	-0.002302522	-0.003494740	0.001916816
3	6	0.000601452	0.000131834	-0.002717269
4	8	-0.001858694	0.002221590	-0.005860640
5	6	-0.000068754	-0.000360850	0.001026886
6	6	0.001926900	0.002260890	-0.000959029
7	6	-0.001933076	-0.007506888	-0.001116722
8	6	0.000876375	-0.004013662	0.019841454
9	6	0.005948715	0.003480065	-0.001267219
10	6	0.001390819	-0.012194455	0.002504977
11	6	-0.008415778	-0.000767753	0.002147718
12	6	-0.006397022	-0.004019096	-0.002489710
13	6	-0.004197675	-0.003715218	-0.000946728
14	6	0.013288971	-0.008285736	0.004924811
15	6	-0.008537207	0.003719137	-0.003366599
16	6	-0.002622170	-0.001488621	0.004846347
17	6	-0.008688293	0.008443704	-0.001300384
18	6	0.004332164	0.004637956	-0.000395049
19	6	-0.000855862	0.000534426	0.000091098
20	8	-0.001040914	0.000128111	0.002682455
21	6	0.006598864	0.006053848	0.001855079
22	6	0.001773717	-0.010069392	-0.007733451
23	6	0.001235566	-0.001829262	0.009459886
24	6	0.002223437	0.002040434	0.002748863
25	6	-0.003892179	0.003998745	-0.014435538
26	6	0.000084318	-0.000597362	-0.000951274
27	6	-0.001338829	0.002979431	0.003038657
28	6	-0.000596818	-0.000217494	0.000837662
29	6	0.002230775	-0.003623750	-0.011425372
30	6	-0.003773946	0.002202135	0.001079174
31	6	0.004242273	-0.001635456	-0.001286182
32	6	0.002159816	0.004163001	-0.006363641
33	6	0.001312868	-0.000314164	-0.000683191
34	6	-0.000893135	-0.000065415	-0.000444082
35	6	0.002058961	0.001032875	-0.001730320
36	6	0.000051058	-0.000060073	-0.000068296
37	6	0.000677627	-0.000376937	0.000435570
38	6	-0.001833339	-0.000511720	-0.001663517
39	1	0.000184597	-0.000003469	0.000018694
40	1	-0.000237199	-0.000130382	-0.000259290
41	1	-0.000453242	0.001164127	0.000400790
42	1	-0.000007592	-0.001302660	0.000153754
43	1	-0.000352740	0.003050926	0.000281306
44	1	-0.000062852	-0.000003282	0.000084865
45	1	0.000273439	0.001396361	-0.000300911
46	1	0.000720221	-0.000168105	-0.000676493
47	1	0.001224143	0.000956662	-0.002741339
48	1	0.000138660	0.000169273	0.000582909
49	1	-0.000980177	0.000577679	-0.000992370
50	1	0.000114832	-0.002550149	-0.000096935

51	1	0.001025416	0.001201124	0.001220492
52	1	0.000542388	0.000091003	-0.000304479
53	1	-0.000160116	0.000102852	0.000083034
54	1	0.008925624	0.002203961	0.010737455
55	1	-0.004954419	0.009177577	-0.003156346
56	1	0.000170585	-0.000145772	0.000097031
57	1	-0.000239617	-0.000134029	0.000141325
58	1	-0.000216285	-0.000313427	0.002161870
59	6	0.000660496	0.000058553	-0.000353240
60	6	-0.000470404	0.000015114	0.000162997
61	1	0.000199485	0.000210255	0.000837286
62	1	0.000106220	0.000453037	-0.000085904
63	6	0.001108553	0.000046920	-0.000136208
64	1	0.001418318	-0.000664637	0.001359477
65	1	-0.001499183	-0.000237878	0.000757429
66	1	0.000020890	0.000012076	0.000005290
67	1	-0.000017048	-0.000044788	-0.000039520
68	1	0.000261906	0.000044401	-0.000321092
69	1	-0.000062426	0.000110791	0.000026323
70	1	0.000524325	0.000497940	-0.000678763
71	1	-0.000256361	-0.001115745	0.000629194
72	1	-0.000980173	0.000871525	-0.000418565
73	6	0.000369043	0.000354163	-0.000477994
74	1	0.000373891	0.000232372	-0.000105768
75	1	-0.000092285	-0.000015401	0.000419311
76	1	-0.000320280	-0.000189595	-0.000389994
77	1	0.000333430	0.000346403	-0.000430657
78	1	0.000121746	-0.000658666	-0.000210042
79	6	0.000248280	-0.000161454	0.000111245
80	1	-0.000167593	-0.000348430	0.000375200
81	1	0.000111939	0.000427727	0.000143106
82	1	-0.000025925	-0.000115054	-0.000566033
83	1	-0.001252885	-0.000406622	0.000424794
84	1	-0.000457255	0.000455198	0.000266365
85	6	0.000141896	0.000617836	-0.000200824
86	1	0.000595666	-0.000067490	0.000046862
87	1	-0.000377292	-0.000230773	0.000032639
88	1	-0.000161088	0.000551181	-0.000366860

Determination of the crystal structures of 3

Single crystals suitable for X-ray investigation were obtained by slow evaporation of a chloroform / methanol solution of **3**. Data collection was carried out at the Macromolecular crystallography XRD1 beamline of the Elettra synchrotron (Trieste, Italy), employing the rotating-crystal method with a Dectris Pilatus 2M area detector. The single crystals investigated were dipped in a cryo-protectant (glycerol), mounted on a loop and flash-frozen under a liquid nitrogen stream at 100 K. Diffraction data were indexed and integrated using the XDS package,[4] while scaling was carried out with XSCALE.[5] The structures were solved using the SHELXT package;[6] and structure refinement was performed with SHELXL-14,[7] operating through the WinGX GUI,[8] by full-matrix least-squares (FMLS) methods on F^2.The asymmetric unit of the orthorhombic crystal (space group Pcab) is composed of one molecule of **3.** All non-hydrogen atoms were anisotropically refined. Hydrogen atoms were added at the calculated positions and refined using the riding model. Crystallographic data and refinement details are reported in Table S2. The dihydropyran ring shows a two position disorder of two contiguous sp^3 carbon atoms bonded to the oxygen atom with refined occupancy factors 70/30 %. This results in two inverted half-chair conformations of the dihydropyran ring.

Table S3. Crystal data and structure refinement for compound **3**.

Empirical formula	$C_{40}H_{46}O_4$
Formula weight	590.77
Temperature (K)	100(2)
Wavelength (Å)	0.7
Crystal system	Orthorombic
Space group	*P*cab
Unit cell dimensions (Å)	*a* = 16.029(5)
	b = 20.169(2)
	c = 20.514(3)
Volume (Å^3)	6632(2)
Z	8
ρ_{calcd} (g/cm^3)	1.183
μ (mm^{-1})	0.072
F(000)	2544
Reflections collected	133396
Independent reflections	11528 [R(int) = 0.0369]

Data / restraints / parameters	11528 / 0 / 420
GooF	1.046
Final *R* indices [I>2σ(I)]	R_1 = 0.0404, wR_2 = 0.1102
R indices (all data)	R_1 = 0.0458, wR_2 = 0.1145
CCDC code	1979551

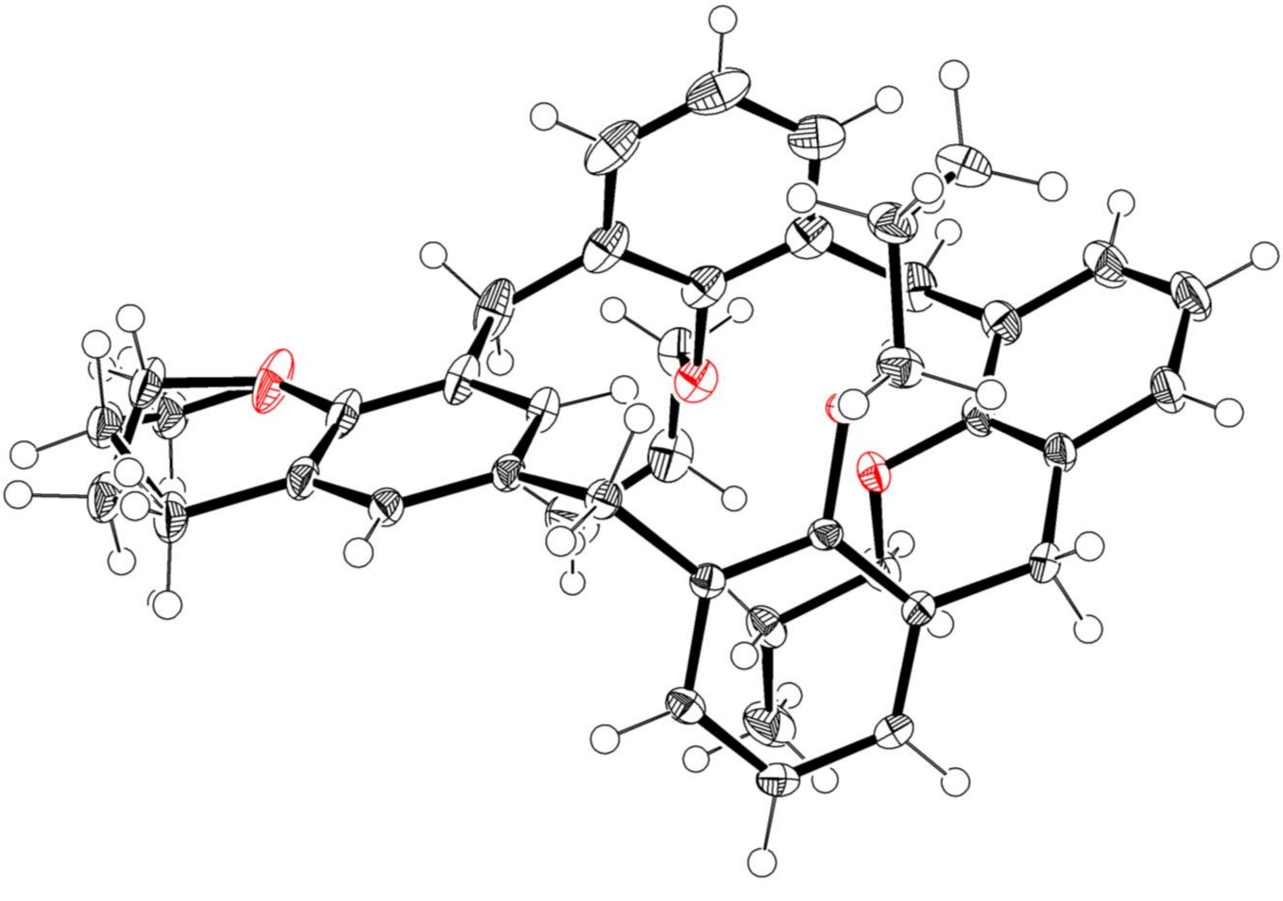

Figure S23. ORTEP drawing of **3** with ellipsoids at 50% probability level, the hydrogen atoms are shown as small sphere of arbitrary radii. The atomic species are represented in CPK colours.

DFT calculation

Conformational studies have been performed using the DFT method incorporated in the Gaussian 16 package,[3] and using B3LYP/6-31G(d,p) level of theory. The starting structure for DFT calculations were obtained by molecular mechanics calculation performed by YASARA software.

DFT optimized structure of 2

C	-1.99146300	-0.85275400	0.00019700
C	-2.20443800	-2.23721600	-0.05156600
C	-3.52152200	-2.69947900	-0.11532000
C	-3.04545400	0.07183200	-0.01244700
C	-1.79390800	1.92259900	-2.22955900
C	-0.74995600	2.28523200	-3.07681100
C	0.45521100	2.73168900	-2.53702300
C	0.62000700	2.86960000	-1.15481300
C	-0.47326400	2.56936400	-0.32149700
C	-1.67148700	2.04682800	-0.84014100
C	4.94331200	1.68369000	-2.42509800
C	4.85470900	0.35614900	-2.00577400
C	3.89202100	-0.04039800	-1.06923900
C	3.03382800	0.94125900	-0.54262100
C	3.05615100	2.26848500	-1.00669200
C	4.03362000	2.62546700	-1.94374300
C	0.10738000	-2.86625600	-0.92809900
C	1.37898200	-2.53239800	-0.42837700
C	2.39836700	-2.05222500	-1.27061700
C	2.15318700	-2.01136600	-2.64721300
C	0.92345700	-2.40779500	-3.17104500
C	-0.09777200	-2.80584400	-2.31191400
O	-0.35192300	2.74709400	1.04972300
C	-0.74068300	4.05465600	1.50598600
C	-0.56768500	4.11417700	3.01609400
C	-0.95953000	5.47962500	3.58922600
O	1.61875500	-2.63627400	0.93505500
C	2.13184100	-3.91747400	1.34043600
C	2.34242000	-3.90356500	2.84687400
C	2.88853600	-5.23729800	3.36590800
O	2.09731800	0.57780600	0.40666100
C	2.56083600	0.70744700	1.76445700
C	1.44033400	0.30344000	2.70891400
C	1.86417700	0.41544800	4.17717900
C	3.70398500	-1.50484300	-0.70431100
C	1.97871600	3.25147800	-0.57663400
C	-2.79252900	1.57301200	0.06736100
C	-1.04043500	-3.22100000	0.00154100
H	-3.72011200	-3.76857000	-0.15163300
H	-2.71622800	1.52235800	-2.64252500
H	-0.86278900	2.19106400	-4.15293200
H	1.29205300	2.95565700	-3.19293300
H	5.70121300	1.97754100	-3.14532100
H	5.53446600	-0.38804000	-2.41315300
H	4.07321000	3.65085700	-2.30264500
H	2.92966400	-1.64018400	-3.31066600
H	0.75263200	-2.37519600	-4.24312300
H	-1.07619900	-3.06094100	-2.71058100
H	-1.78630000	4.24784400	1.22617700
H	-0.12051900	4.81686600	1.01235800
H	0.47702700	3.88748000	3.26034300
H	-1.17656800	3.32425800	3.47155500
H	-0.82679100	5.49931200	4.67482600
H	-0.34640100	6.28217900	3.16453300
H	-2.00850300	5.71500700	3.37830000
H	3.07751100	-4.11824100	0.81637900
H	1.42226700	-4.70726400	1.05452300
H	1.38793800	-3.67093600	3.33376100
H	3.03186100	-3.08909200	3.09922000
H	3.03192400	-5.20445700	4.44981700
H	2.20202900	-6.06238000	3.14622900
H	3.85506100	-5.47808700	2.90963000
H	2.87116800	1.74631500	1.94836900

H	3.44519900	0.07092600	1.91281900
H	1.14947200	-0.72453200	2.47321600
H	0.57716200	0.94566200	2.51055200
H	1.04662300	0.12156000	4.84255000
H	2.72046700	-0.23177100	4.40029400
H	2.14910900	1.44156800	4.43758500
H	4.55258400	-2.07466100	-1.09972000
H	3.69918000	-1.63816500	0.37869900
H	2.26341000	4.25358300	-0.91748300
H	1.89789500	3.28890800	0.51090300
H	-3.72181000	2.09076800	-0.18515500
H	-2.55394300	1.83907500	1.10127900
H	-1.41901200	-4.22117300	-0.24052700
H	-0.65959100	-3.26981000	1.02593300
H	-0.97279200	-0.47629600	0.03046100
C	-4.60512000	-1.81361300	-0.12954400
C	-4.35352500	-0.42762500	-0.08266000
O	-5.39920600	0.46447000	-0.02775600
C	-6.62803800	0.06506200	-0.66006500
C	-6.97894400	-1.37081700	-0.38139200
C	-5.99867300	-2.25494500	-0.15442000
H	-7.38597200	0.74948900	-0.26987300
H	-8.02539600	-1.65919900	-0.40993100
H	-6.21164500	-3.30604100	0.02188600
H	-6.55001000	0.24420700	-1.74668600

1 2 1.5 4 1.5 78 1.0
2 3 1.5 38 1.0
3 39 1.0 79 1.5
4 37 1.0 80 1.5
5 6 1.5 10 1.5 40 1.0
6 7 1.5 41 1.0
7 8 1.5 42 1.0
8 9 1.5 36 1.0
9 10 1.5 23 1.0
10 37 1.0
11 12 1.5 16 1.5 43 1.0
12 13 1.5 44 1.0
13 14 1.5 35 1.0
14 15 1.5 31 1.0
15 16 1.5 36 1.0
16 45 1.0
17 18 1.5 22 1.5 38 1.0
18 19 1.5 27 1.0
19 20 1.5 35 1.0
20 21 1.5 46 1.0
21 22 1.5 47 1.0
22 48 1.0
23 24 1.0
24 25 1.0 49 1.0 50 1.0
25 26 1.0 51 1.0 52 1.0
26 53 1.0 54 1.0 55 1.0
27 28 1.0
28 29 1.0 56 1.0 57 1.0
29 30 1.0 58 1.0 59 1.0
30 60 1.0 61 1.0 62 1.0
31 32 1.0
32 33 1.0 63 1.0 64 1.0
33 34 1.0 65 1.0 66 1.0
34 67 1.0 68 1.0 69 1.0
35 70 1.0 71 1.0
36 72 1.0 73 1.0
37 74 1.0 75 1.0
38 76 1.0 77 1.0
39
40
41
42
43
44
45
46
47
48
49
50
51
52

53
54
55
56
57
58
59
60
61
62
63
64
65
66
67
68
69
70
71
72
73
74
75
76
77
78
79 80 1.5 84 1.0
80 81 1.0
81 82 1.0
82 83 1.0 85 1.0 88 1.0
83 84 2.0 86 1.0
84 87 1.0
85
86
87
88

0 imaginary frequency

DFT optimized structure of 3

C	1.91587400	1.20171000	-0.62318200
C	2.07057300	2.44877200	0.00237300
C	3.34191800	2.82047900	0.43926600
C	2.98800600	0.32554600	-0.81466500
C	1.19989300	-0.71311100	-3.39015000
C	-0.07148700	-0.84788100	-3.94502900
C	-1.06529900	-1.51632100	-3.23456200
C	-0.80057700	-2.09521000	-1.98726900
C	0.50210100	-1.99252800	-1.46891900
C	1.50467100	-1.27056500	-2.14342900
C	-5.06600300	-0.61096300	-1.34758400
C	-4.82930000	0.29916100	-0.31957000
C	-3.70919100	0.18475900	0.51481700
C	-2.82208400	-0.88469700	0.29203900
C	-2.99635500	-1.76477500	-0.79686600
C	-4.14396100	-1.62771200	-1.58753400
C	-0.09367800	2.90126600	1.26350800
C	-1.32486100	2.30218300	0.93920600
C	-2.19408300	1.82097900	1.93724000
C	-1.81303400	1.99408000	3.27369700
C	-0.60754000	2.60466600	3.61695600
C	0.24928600	3.04463200	2.61327300
O	0.80762100	-2.58719300	-0.25320700
C	1.29872800	-3.93432700	-0.37259400
C	1.68128700	-4.43781600	1.01111600
C	2.23746200	-5.86464500	0.97342200
O	-1.64450700	2.13652000	-0.39843600
C	-2.49571300	3.14872300	-0.95847500
C	-2.64455200	2.87624000	-2.44784400
C	-3.54884000	3.90286100	-3.13637300
O	-1.71515900	-1.00625500	1.10566400
C	-1.69169700	-2.11059800	2.02863200

C	-2.33754200	-1.78351300	3.37296600
C	-2.20648200	-2.94847100	4.36015100
C	-3.54667300	1.17951500	1.65473900
C	-1.92201300	-2.76582700	-1.20171800
C	2.85849800	-1.02282400	-1.51701400
C	0.87299800	3.36989600	0.18554100
H	3.49606900	3.79203200	0.90396500
H	1.96949400	-0.15806100	-3.92055700
H	-0.29217800	-0.41626700	-4.91686200
H	-2.06850400	-1.58576900	-3.64561700
H	-5.95160600	-0.51626200	-1.96893600
H	-5.53204400	1.11056400	-0.14687100
H	-4.30248800	-2.31949800	-2.41103400
H	-2.47637100	1.63824100	4.05793600
H	-0.33629900	2.72545300	4.66167800
H	1.20021500	3.50408500	2.87048900
H	2.16805300	-3.95388800	-1.04537000
H	0.52353500	-4.57340700	-0.82039700
H	0.79876700	-4.39490500	1.66014200
H	2.42171500	-3.75306000	1.44109800
H	2.50312500	-6.20624100	1.97806900
H	1.50403600	-6.56927100	0.56591100
H	3.13798900	-5.92571700	0.35245300
H	-2.05454800	4.14109000	-0.78423100
H	-3.47723800	3.13133300	-0.46462000
H	-3.04776600	1.86601700	-2.57704200
H	-1.64884700	2.87965700	-2.90683100
H	-3.63919000	3.68866900	-4.20540800
H	-4.55835200	3.89557300	-2.71025400
H	-3.15240100	4.91931900	-3.03400500
H	-0.63306600	-2.35162600	2.15720600
H	-2.18589300	-2.98490100	1.58736600
H	-3.39770600	-1.54923400	3.21692900
H	-1.86557000	-0.88416700	3.78227700
H	-2.67154800	-2.70482400	5.32007400
H	-2.69096800	-3.85402200	3.97745900
H	-1.15509900	-3.19015800	4.55242600
H	-4.28133700	1.97793400	1.48773000
H	-3.86404700	0.69514600	2.58588000
H	-2.38840200	-3.53694600	-1.82510600
H	-1.49341200	-3.26692600	-0.33592400
H	3.63268500	-1.08091900	-2.29102900
H	3.09905000	-1.80966400	-0.79650300
H	0.33972300	3.45830800	-0.76352700
H	1.23539100	4.37009100	0.44780800
H	0.92989900	0.91176800	-0.97118200
C	4.44255000	1.96606600	0.28186300
C	4.24669200	0.71674600	-0.33282100
O	5.30505500	-0.12895300	-0.56163500
C	6.42960400	-0.02540400	0.33078000
C	6.77083200	1.39951500	0.67149600
C	5.80477100	2.32743400	0.67023300
H	6.21442500	-0.60430000	1.24569800
H	7.79521800	1.62911900	0.94898300
H	6.00961500	3.36013500	0.94021400
H	7.25313800	-0.52569100	-0.18584500

1 2 1.5 4 1.5 78 1.0
2 3 1.5 38 1.0
3 39 1.0 79 1.5
4 37 1.0 80 1.5
5 6 1.5 10 1.5 40 1.0
6 7 1.5 41 1.0
7 8 1.5 42 1.0
8 9 1.5 36 1.0
9 10 1.5 23 1.0
10 37 1.0
11 12 1.5 16 1.5 43 1.0
12 13 1.5 44 1.0
13 14 1.5 35 1.0
14 15 1.5 31 1.0
15 16 1.5 36 1.0

16 45 1.0
17 18 1.5 22 1.5 38 1.0
18 19 1.5 27 1.0
19 20 1.5 35 1.0
20 21 1.5 46 1.0
21 22 1.5 47 1.0
22 48 1.0
23 24 1.0
24 25 1.0 49 1.0 50 1.0
25 26 1.0 51 1.0 52 1.0
26 53 1.0 54 1.0 55 1.0
27 28 1.0
28 29 1.0 56 1.0 57 1.0
29 30 1.0 58 1.0 59 1.0
30 60 1.0 61 1.0 62 1.0
31 32 1.0
32 33 1.0 63 1.0 64 1.0
33 34 1.0 65 1.0 66 1.0
34 67 1.0 68 1.0 69 1.0
35 70 1.0 71 1.0
36 72 1.0 73 1.0
37 74 1.0 75 1.0
38 76 1.0 77 1.0
39
40
41
42
43
44
45
46
47
48
49
50
51
52
53
54
55
56
57
58
59
60
61
62
63
64
65
66
67
68
69
70
71
72
73
74
75
76
77
78
79 80 1.5 84 1.0
80 81 1.0
81 82 1.0
82 83 1.0 85 1.0 88 1.0
83 84 2.0 86 1.0
84 87 1.0
85
86

87
88

0 imaginary frequency

DFT optimized structure of *syn-propoxy n*BuNH$_3^+$@6

C	-1.47020000	-1.87690000	-0.45930000
C	-1.69020000	-2.53760000	0.76370000
C	-3.00200000	-2.63730000	1.23060000
C	-2.51660000	-1.30490000	-1.18640000
C	-0.36370000	-1.51480000	-3.79970000
C	0.97000000	-1.48060000	-4.20080000
C	1.79570000	-0.44700000	-3.77120000
C	1.30830000	0.58850000	-2.96230000
C	-0.05460000	0.56070000	-2.62630000
C	-0.89990000	-0.49870000	-3.00280000
C	5.33920000	-0.23740000	-1.22430000
C	5.03150000	-0.44320000	0.11840000
C	3.89740000	0.13550000	0.70120000
C	3.09030000	0.94730000	-0.11120000
C	3.29990000	1.06040000	-1.49710000
C	4.46490000	0.49020000	-2.02690000
C	0.26370000	-2.08450000	2.32600000
C	1.52460000	-1.63140000	1.88440000
C	2.22510000	-0.61950000	2.57650000
C	1.64290000	-0.09650000	3.73900000
C	0.41090000	-0.55260000	4.20820000
C	-0.27610000	-1.53340000	3.49640000
O	-0.59740000	1.61070000	-1.86720000
C	-1.10520000	2.70790000	-2.66770000
C	-1.66250000	3.78390000	-1.74970000
C	-2.20070000	4.98490000	-2.53560000
O	2.03370000	-2.15060000	0.71220000
C	2.92970000	-3.27290000	0.85840000
C	3.25130000	-3.80800000	-0.52780000
C	4.21660000	-4.99640000	-0.47700000
O	2.02480000	1.64190000	0.47950000
C	2.23350000	3.07040000	0.63700000
C	3.03000000	3.43870000	1.88330000
C	3.16810000	4.95830000	2.03300000
C	3.60660000	-0.11520000	2.17140000
C	2.25960000	1.66400000	-2.43480000
C	-2.33090000	-0.59280000	-2.52330000
C	-0.52550000	-3.12960000	1.54630000
C	-4.08490000	-2.09070000	0.53000000
C	-3.82660000	-1.41090000	-0.67080000
O	-4.80770000	-0.84350000	-1.43600000
C	-6.13340000	-0.77570000	-0.88290000
C	-6.50180000	-2.04730000	-0.13270000
C	-5.51080000	-2.26050000	1.01680000
H	-3.20370000	-3.18120000	2.15120000
H	-1.00390000	-2.33740000	-4.10550000
H	1.36850000	-2.26560000	-4.83560000
H	2.84170000	-0.44720000	-4.05850000
H	6.24370000	-0.66200000	-1.64800000
H	5.68880000	-1.04950000	0.73530000
H	4.68320000	0.61640000	-3.08330000
H	2.17760000	0.66960000	4.29450000
H	-0.00470000	-0.15030000	5.12740000
H	-1.23840000	-1.89050000	3.85370000
H	-1.87990000	2.32810000	-3.34520000
H	-0.28640000	3.10220000	-3.28190000
H	-0.87130000	4.11110000	-1.06260000
H	-2.46450000	3.35140000	-1.13730000
H	-2.59780000	5.74580000	-1.85870000
H	-1.41430000	5.45260000	-3.13660000
H	-3.00800000	4.68830000	-3.21280000
H	2.45270000	-4.04350000	1.47980000
H	3.84470000	-2.95070000	1.37320000

H	3.67750000	-2.99320000	-1.12310000
H	2.31480000	-4.10020000	-1.01770000
H	4.43090000	-5.36580000	-1.48350000
H	5.17120000	-4.71900000	-0.01680000
H	3.79990000	-5.82990000	0.09880000
H	1.22800000	3.50510000	0.68210000
H	2.72510000	3.46090000	-0.26020000
H	4.02350000	2.98110000	1.82040000
H	2.53280000	3.01500000	2.76390000
H	3.74150000	5.20860000	2.92920000
H	3.68680000	5.39900000	1.17530000
H	2.19060000	5.44560000	2.11910000
H	4.35390000	-0.83290000	2.53100000
H	3.79290000	0.80090000	2.74180000
H	2.77490000	2.13410000	-3.27940000
H	1.68070000	2.43910000	-1.93530000
H	-2.92700000	-1.12190000	-3.27590000
H	-2.78920000	0.39830000	-2.44940000
H	0.14170000	-3.65150000	0.85840000
H	-0.91240000	-3.87410000	2.24990000
H	-0.46020000	-1.83250000	-0.85820000
H	-6.19060000	0.09530000	-0.21380000
H	-7.52770000	-1.96820000	0.24050000
H	-6.78840000	-0.59440000	-1.73760000
H	-5.63470000	-3.25590000	1.45610000
H	-5.72310000	-1.54230000	1.82120000
H	-6.46940000	-2.89510000	-0.82610000
N	-0.65320000	0.97070000	0.86070000
C	-1.37450000	1.80380000	1.87910000
C	-2.88070000	1.55520000	1.84070000
C	-3.64720000	2.35140000	2.91110000
C	-3.61100000	3.87280000	2.72650000
H	-0.87190000	-0.02760000	0.97300000
H	-0.89430000	1.23120000	-0.11660000
H	0.38140000	1.08370000	0.91340000
H	-1.12710000	2.84590000	1.66500000
H	-0.95410000	1.54030000	2.85210000
H	-3.26850000	1.80880000	0.84490000
H	-3.06310000	0.48300000	1.98390000
H	-4.68920000	2.01340000	2.89600000
H	-3.26020000	2.08770000	3.90400000
H	-4.23400000	4.36730000	3.47680000
H	-3.99080000	4.16200000	1.74010000
H	-2.60030000	4.28220000	2.82970000

1 2 1.5 4 1.5 84 1.0
2 3 1.5 38 1.0
3 39 1.5 45 1.0
4 37 1.0 40 1.5
5 6 1.5 10 1.5 46 1.0
6 7 1.5 47 1.0
7 8 1.5 48 1.0
8 9 1.5 36 1.0
9 10 1.5 23 1.0
10 37 1.0
11 12 1.5 16 1.5 49 1.0
12 13 1.5 50 1.0
13 14 1.5 35 1.0
14 15 1.5 31 1.0
15 16 1.5 36 1.0
16 51 1.0
17 18 1.5 22 1.5 38 1.0
18 19 1.5 27 1.0
19 20 1.5 35 1.0
20 21 1.5 52 1.0
21 22 1.5 53 1.0
22 54 1.0
23 24 1.0
24 25 1.0 55 1.0 56 1.0
25 26 1.0 57 1.0 58 1.0
26 59 1.0 60 1.0 61 1.0
27 28 1.0

28 29 1.0 62 1.0 63 1.0
29 30 1.0 64 1.0 65 1.0
30 66 1.0 67 1.0 68 1.0
31 32 1.0
32 33 1.0 69 1.0 70 1.0
33 34 1.0 71 1.0 72 1.0
34 73 1.0 74 1.0 75 1.0
35 76 1.0 77 1.0
36 78 1.0 79 1.0
37 80 1.0 81 1.0
38 82 1.0 83 1.0
39 40 1.5 44 1.0
40 41 1.0
41 42 1.0
42 43 1.0 85 1.0 87 1.0
43 44 1.0 86 1.0 90 1.0
44 88 1.0 89 1.0
45
46
47
48
49
50
51
52
53
54
55
56
57
58
59
60
61
62
63
64
65
66
67
68
69
70
71
72
73
74
75
76
77
78
79
80
81
82
83
84
85
86
87
88
89
90
91 92 1.0 96 1.0 97 1.0 98 1.0
92 93 1.0 99 1.0 100 1.0
93 94 1.0 101 1.0 102 1.0
94 95 1.0 103 1.0 104 1.0
95 105 1.0 106 1.0 107 1.0
96
97
98

99
100
101
102
103
104
105
106
107

0 imaginary frequency

DFT optimized structure of *syn-phenol n*BuNH$_3^+$@6

C	-1.45890000	-0.63540000	-1.83100000
C	-2.02570000	0.58350000	-2.23180000
C	-3.40490000	0.74600000	-2.06420000
C	-2.23100000	-1.66500000	-1.26960000
C	-1.77700000	-3.26480000	1.66680000
C	-1.23270000	-3.22420000	2.95040000
C	0.09880000	-2.84680000	3.13190000
C	0.91040000	-2.51530000	2.03940000
C	0.35230000	-2.60090000	0.74780000
C	-0.99390000	-2.96000000	0.54510000
C	1.70800000	1.63160000	3.24700000
C	2.12950000	2.31300000	2.10400000
C	2.72720000	1.63620000	1.03530000
C	2.93620000	0.24430000	1.15400000
C	2.38440000	-0.48080000	2.23370000
C	1.80030000	0.24120000	3.28730000
C	0.28910000	1.60830000	-2.75840000
C	0.98120000	2.09260000	-1.63470000
C	2.36980000	1.93650000	-1.48190000
C	3.07150000	1.32480000	-2.52730000
C	2.41840000	0.87220000	-3.67060000
C	1.03570000	1.00090000	-3.77530000
O	1.12180000	-2.25350000	-0.34780000
C	1.87650000	-3.33760000	-0.93510000
C	2.53890000	-2.83220000	-2.20610000
C	3.41740000	-3.90290000	-2.86130000
O	0.24490000	2.72690000	-0.62140000
C	0.15170000	4.16660000	-0.76530000
C	-0.75710000	4.72290000	0.31880000
C	-0.86390000	6.25040000	0.24870000
O	3.64420000	-0.39660000	0.16540000
C	4.96170000	-0.87440000	0.53190000
C	6.03900000	0.20290000	0.45190000
C	7.42060000	-0.35250000	0.81660000
C	3.11680000	2.38490000	-0.23010000
C	2.33290000	-2.00700000	2.24830000
C	-1.59380000	-2.98480000	-0.85470000
C	-1.21800000	1.70690000	-2.88440000
H	-3.87310000	1.68010000	-2.37000000
H	-2.81640000	-3.54180000	1.51940000
H	-1.84240000	-3.49070000	3.80830000
H	0.51890000	-2.81660000	4.13370000
H	1.29340000	2.17760000	4.08910000
H	2.01280000	3.39140000	2.04330000
H	1.41340000	-0.30140000	4.14540000
H	4.14560000	1.20080000	-2.43440000
H	2.98480000	0.41270000	-4.47430000
H	0.52110000	0.63360000	-4.65890000
H	1.20310000	-4.17970000	-1.14560000
H	2.62750000	-3.69420000	-0.21700000
H	3.12640000	-1.94340000	-1.95500000
H	1.76000000	-2.50440000	-2.90470000
H	3.88000000	-3.52070000	-3.77550000
H	4.22340000	-4.22650000	-2.19340000
H	2.83660000	-4.79110000	-3.13270000
H	-0.24000000	4.39830000	-1.76310000

H	1.15660000	4.60020000	-0.69430000
H	-0.36920000	4.41830000	1.29900000
H	-1.75240000	4.27360000	0.21190000
H	-1.52470000	6.62950000	1.03250000
H	0.11360000	6.72550000	0.38000000
H	-1.26860000	6.57870000	-0.71420000
H	5.17380000	-1.68250000	-0.17490000
H	4.93500000	-1.30450000	1.54060000
H	5.77940000	1.02590000	1.12880000
H	6.05750000	0.61390000	-0.56440000
H	8.18370000	0.42720000	0.74790000
H	7.43740000	-0.74300000	1.83970000
H	7.71590000	-1.16490000	0.14400000
H	2.95850000	3.45360000	-0.05300000
H	4.18610000	2.26110000	-0.41970000
H	2.70040000	-2.37690000	3.21110000
H	2.98740000	-2.40440000	1.47540000
H	-2.35260000	-3.76840000	-0.89790000
H	-0.81930000	-3.23360000	-1.58210000
H	-1.57280000	2.66030000	-2.48010000
H	-1.46600000	1.72820000	-3.95280000
H	-0.39500000	-0.80810000	-1.96410000
C	-4.21810000	-0.25660000	-1.52740000
C	-3.61720000	-1.46360000	-1.12140000
O	-4.32900000	-2.48680000	-0.55390000
C	-5.75790000	-2.46040000	-0.72120000
C	-6.32490000	-1.08060000	-0.41920000
C	-5.71590000	-0.06040000	-1.38800000
H	-5.99910000	-2.76320000	-1.74940000
H	-7.41560000	-1.10140000	-0.50720000
H	-5.93070000	0.96330000	-1.06200000
H	-6.13750000	-3.22340000	-0.03900000
H	-6.08660000	-0.81850000	0.61860000
H	-6.18620000	-0.16920000	-2.37450000
C	-1.66200000	0.75360000	2.19380000
N	-0.71420000	0.63510000	1.03070000
H	0.15870000	0.17950000	1.32700000
H	-1.11670000	0.06520000	0.26920000
H	-0.45430000	1.54840000	0.60480000
C	-2.96280000	1.44800000	1.80890000
H	-1.83720000	-0.26430000	2.55060000
H	-1.12480000	1.30190000	2.97000000
C	-3.92630000	1.53980000	3.00200000
H	-2.74450000	2.45720000	1.43660000
H	-3.44070000	0.90330000	0.98550000
C	-5.23820000	2.24560000	2.64830000
H	-4.14000000	0.52910000	3.37280000
H	-3.43500000	2.07190000	3.82690000
H	-5.90250000	2.29570000	3.51530000
H	-5.05970000	3.27090000	2.30680000
H	-5.77210000	1.71690000	1.85090000

1 2 1.5 4 1.5 78 1.0
2 3 1.5 38 1.0
3 39 1.0 79 1.5
4 37 1.0 80 1.5
5 6 1.5 10 1.5 40 1.0
6 7 1.5 41 1.0
7 8 1.5 42 1.0
8 9 1.5 36 1.0
9 10 1.5 23 1.0
10 37 1.0
11 12 1.5 16 1.5 43 1.0
12 13 1.5 44 1.0
13 14 1.5 35 1.0
14 15 1.5 31 1.0
15 16 1.5 36 1.0
16 45 1.0
17 18 1.5 22 1.5 38 1.0
18 19 1.5 27 1.0
19 20 1.5 35 1.0
20 21 1.5 46 1.0

21 22 1.5 47 1.0
22 48 1.0
23 24 1.0
24 25 1.0 49 1.0 50 1.0
25 26 1.0 51 1.0 52 1.0
26 53 1.0 54 1.0 55 1.0
27 28 1.0
28 29 1.0 56 1.0 57 1.0
29 30 1.0 58 1.0 59 1.0
30 60 1.0 61 1.0 62 1.0
31 32 1.0
32 33 1.0 63 1.0 64 1.0
33 34 1.0 65 1.0 66 1.0
34 67 1.0 68 1.0 69 1.0
35 70 1.0 71 1.0
36 72 1.0 73 1.0
37 74 1.0 75 1.0
38 76 1.0 77 1.0
39
40
41
42
43
44
45
46
47
48
49
50
51
52
53
54
55
56
57
58
59
60
61
62
63
64
65
66
67
68
69
70
71
72
73
74
75
76
77
78
79 80 1.5 84 1.0
80 81 1.0
81 82 1.0
82 83 1.0 85 1.0 88 1.0
83 84 1.0 86 1.0 89 1.0
84 87 1.0 90 1.0
85
86
87
88
89
90
91 92 1.0 96 1.0 97 1.0 98 1.0

92 93 1.0 94 1.0 95 1.0
93
94
95
96 99 1.0 100 1.0 101 1.0
97
98
99 102 1.0 103 1.0 104 1.0
100
101
102 105 1.0 106 1.0 107 1.0
103
104
105
106
107

0 imaginary frequency

Complexation Studies

A 1:1 mixture of calix[3]arene[1]chromane derivative **6** and *n*-butylammonium•[B(Ar^F)$_4$]$^-$ was dissolved in 0.5 mL of $CDCl_3$ and stirred for 15 min at room temperature. Then, the solution was transferred in an NMR tube for 1D and 2D NMR spectra acquisition.

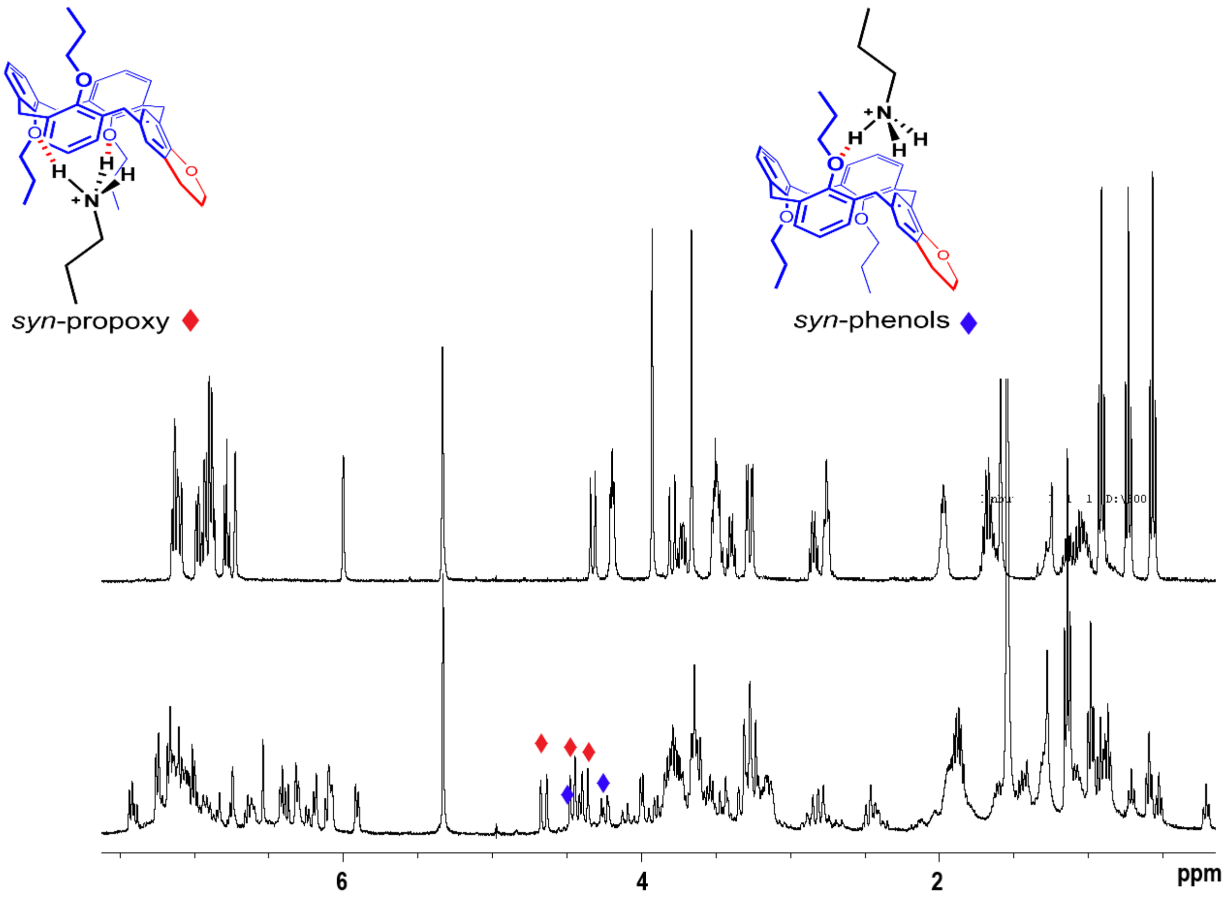

Figure S24. ^{1}H NMR spectra (CD_2Cl_2, 298 K, 600 MHz) of: (top) calix[3]arene[1]chromane **6**; (bottom) 1:1 mixture (2×10^{-3} M) of *n*-butylammonium •[B(Ar^F)$_4$]$^-$ and **6.**

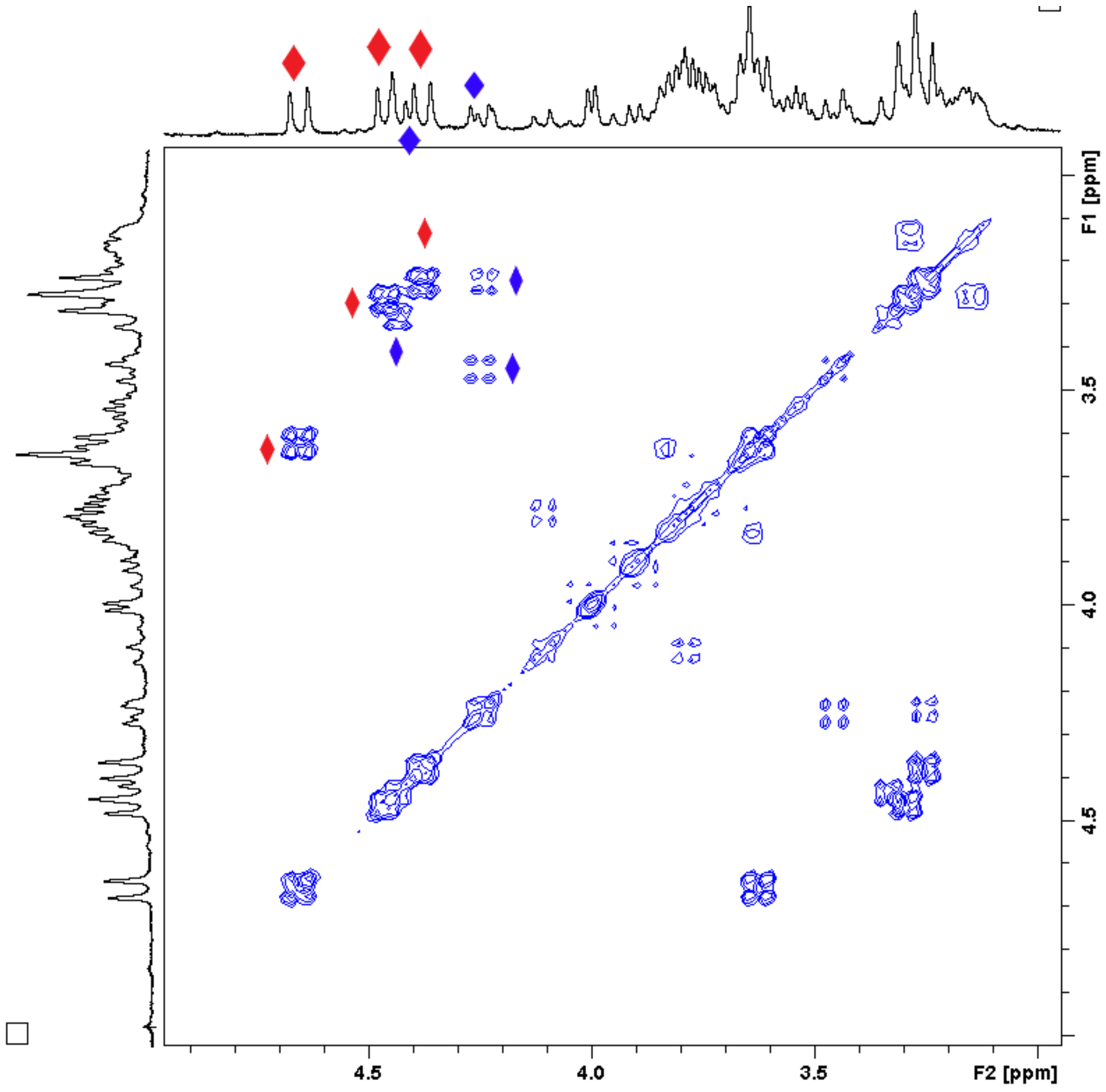

Figure S25. 2D COSY spectrum of the 1:1 mixture (2×10^{-3} M) of *n*-butylammonium•$[B(Ar^F)_4]^-$ and **6.**

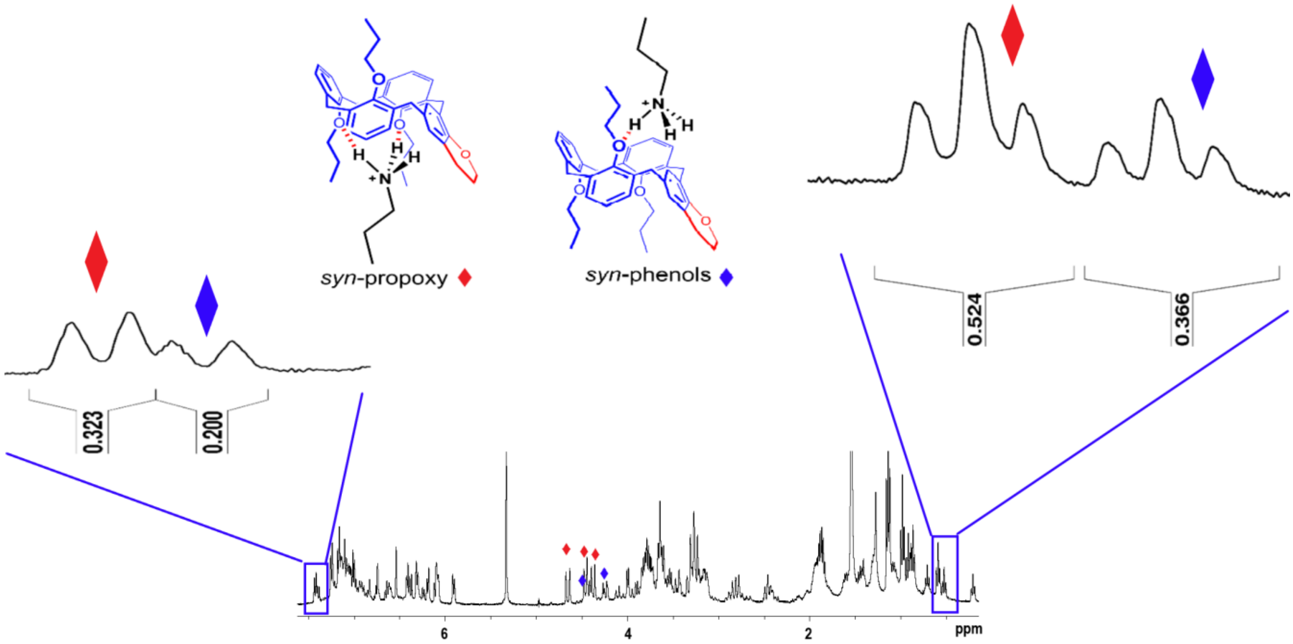

Figure S26. ^{1}H NMR spectrum of the 1:1 mixture (2×10^{-3} M) of *n*-butylammonium•[B(Ar^F)$_4$]$^-$ and **6.** Marked in red and blue the signals of two stereoisomeric *syn*-propoxy and *syn*-phenols complexes ***n*****BuNH$_3^+$@6**,.

From the integrals values: *syn*-propoxy/*syn*-phenols = 60/40

References:

1) Fulmer, G. R.; Miller, A. J. M.; Sherden, N. H.; Gottlieb, H. E.; Nudelman, A.; Stoltz, B.M.; Bercaw, J.E.; Goldberg, K.I. Chemical Shifts of Trace Impurities: Common Laboratory Solvents, Organics, and Gases in Deuterated Solvents Relevant to the Organometallic Chemist. *Organometallics* **2010**, *29*, 2176–2179.

2) Lhoták, P.; Bílá, A.; Budka, J.; Pojarová, M.; Stibor, I. Simple synthesis of calix[4]arenes in a 1,2-alternate conformation. *Chem. Commun.* **2008**, 1662-1664.

3) Gaussian 16, Revision C.01, Frisch, M. J.; Trucks, G. W.; Schlegel, H. B.; Scuseria, G. E.; Robb, M. A.; Cheeseman, J. R.; Scalmani, G.; Barone, V.; Petersson, G. A.; Nakatsuji, H.; Li, M.; Caricato, M.; Marenich, A. V.; Bloino, J.; Janesko, B. G.; Gomperts, R.; Menucci, B.; Hratchian, H. P.; Ortiz, J. V.; Izmaylov, A. F.; Sonnenberg, J. L.; Williams-Young, D.; Ding, F.;Lipparini, F.; Egidi, F.; Goings, J.; Peng, B.; Petrone, A.; Henderson, T.; Ranasinghe, D.; Zakrzewski, V. G.; Gao, J.; Rega, N.; Zheng, G.; Liang, W.; Hada, M.; Ehara, M.; Toyota, K.; Fukuda, R.; Hasegawa, J.; Ishida, M.; Nakajima, T.; Honda, Y.; Kitao, O.; Nakaj, H.; Vreven, T.; Throssell, K.; Montgomery, J. A.; Peralta, Jr. J. E.; Ogliaro, F.; Bearpark, M. J.; Heyd, J. J.; Brothers, E. N.; Kudin, K. N.; Staroveroy, V. N.; Keith, T. A.; Kobayashi, R.; Normand, J.; Raghavachari, K.; Rendell, A. P.; Burant, J. C.; Iyengar, S. S.; Tomasi, J.; Cossi, M.; Millam, J. M.; Klene, M.; Adamo, C.; Cammi, R.; Ochterski, J. W.; Martin, R. L.; Morokuma, K.; Farkas, O.; Foresman, J. B.; Fox, D. J. Gaussian, Inc., Wallingford CT, 2019.

4) Kabsch, W. XDS. *Acta Crystallogr.* **2010**, *D66*, 125-132.

5) Kabsch, W. Integration, scaling, space-group assignment and post-refinement, *Acta Crystallogr.* **2010**, *D66*, 133-144.

6) Sheldrick, G. M. SHELXT-integrated space-group and crystal-structure determination, *Acta Crystallogr*. **2015**, *A71*, 3-8.

7) Sheldrick, G. M. A short history of SHELX, *Acta Crystallogr*. **2008**, *A64*, 112-122.

8) Farrugia, L.J. WinGX and ORTEP for Windows: an update, *J. Appl. Cryst.* **2012**, *45*, 849−85.
